# Algorithmic antibiotic decision-making in urinary tract infection using prescriber-informed prediction of treatment utility

**DOI:** 10.1038/s41746-026-02369-z

**Published:** 2026-01-26

**Authors:** Alex Howard, Peter L. Green, Yinzheng Zhong, David M. Hughes, Alessandro Gerada, Simon Maskell, Anoop Velluva, Iain E. Buchan, William Hope

**Affiliations:** 1 Department of Clinical Pharmacology and Therapeutics, Institute of Systems, Molecular and Integrative Biology, William Henry Duncan Building, University of Liverpool, Liverpool, UK; 2 NHS University Hospitals of Liverpool Group, Mount Vernon Street, Liverpool, UK; 3 Civic Health Innovation Labs, University of Liverpool, Liverpool Science Park, Liverpool, UK; 4 Department of Mechanical and Aerospace Engineering, School of Engineering, University of Liverpool, The Quadrangle, Brownlow Hill, Liverpool, UK; 5 Department of Health Data Science, Institute of Population Health, University of Liverpool, Waterhouse Building Block B, Liverpool, UK; 6 Department of Electrical Engineering and Electronics, School of Electrical Engineering, Electronics, and Computer Science, University of Liverpool, The Quadrangle, Brownlow Hill, Liverpool, UK; 7 Department of Public Health, Policy & Systems, Institute of Population Health, University of Liverpool, Waterhouse Building Block B, Liverpool, UK

**Keywords:** Antibiotics, Antimicrobial resistance, Clinical microbiology, Machine learning

## Abstract

Predicting antibiotic treatment outcomes could help tackle antibiotic resistance by guiding prescribing decisions. Existing approaches do not quantitatively incorporate the judgment of clinician users. Our antibiotic decision-making algorithm predicted treatment outcomes for 13 antibiotics using clinical prediction models trained on prescribing and urine culture data from 93,906 patients, then weighted outcomes using treatment decisions made by 49 clinicians in an antibiotic choice ranking exercise. In a simulation using Emergency Department data, the algorithm chose more correctly-targeted World Health Organization Access category antibiotics (75.6% of cases versus 11.9%, 95% confidence interval of difference 57.6% to 69.7%, *p* < 0.001) and oral antibiotics (69% versus 22.6%, 95% confidence interval of difference 39.5% to 53.4%, *p* < 0.001) than human prescribers, and fewer intravenous antibiotics (31.2% versus 65.8%, 95% confidence interval of difference −41.9% to −27.1%, *p* < 0.001). These results show that our algorithm could improve antibiotic prescribing decisions by combining human judgment with data-driven probability predictions.

## Introduction

Antimicrobial resistance (AMR) threatens the delivery of effective healthcare^1^. The United Nations General Assembly (UNGA) recently committed to prioritizing global use of narrow-spectrum antibiotics that are potentially less likely to generate AMR (World Health Organization [WHO] Access Category)^2^. UNGA set a target for 70% of antibiotic use at a global level to be from antibiotics in the WHO Access category. Clinicians may be reluctant to use Access agents because of uncertainty about the risk of treatment failure, especially in the context of highly prevalent drug resistance. This uncertainty results in the use of broader spectrum agents classified within WHO Watch or Reserve categories^3^.

Algorithms based on probability predictions (for example, those from statistical models/machine learning algorithms) can inform human antibiotic prescribing decisions by using drug, patient, and pathogen characteristics to estimate the probability of various antibiotic treatment outcomes more accurately than clinicians^4^. However, many existing algorithms that have been used for this purpose are reductionist, in that they make treatment recommendations based on relatively narrow considerations (e.g., probability of clinical response alone). To better-inform antibiotic prescribing decisions, algorithms need a way to emulate helpful aspects of human decision making—they need to predict the probability of multiple other treatment outcomes (e.g., drug toxicity), incorporate other drug characteristics (e.g., route of administration, cost), and weigh these factors against each other^5,6^. Algorithms also need to detect when it is likely to be safe to use an oral, narrower-spectrum Access agent (e.g., in uncomplicated urinary tract infection [UTI]), and when an intravenous, broader-spectrum Watch or Reserve agent is required to maximize probability of efficacy (e.g., in urinary sepsis). An algorithm that could perform these functions would be a powerful antibiotic stewardship tool because it could combine the predictive power of statistical modeling techniques with the ability to weigh the risk-versus-benefit of treatment decisions.

Here, we describe the development of an antibiotic decision-making algorithm that directs antibiotic treatment and testing decisions for UTI by predicting antibiotic treatment outcomes using clinical prediction modeling, then weighting outcome importance using a value (utility) based model that leverages the knowledge and experience of clinicians. The algorithm also incorporates a safety mechanism to prioritize treatment efficacy patients with severe infection. We report the results of a simulation study that assesses the ability of our algorithm to make more targeted antibiotic treatment decisions than clinicians did for attendees to an Emergency department.

## Results

### Study population characteristics

Real-world MIMIC-IV^7,8,9^ (Boston, MA) electronic healthcare record urine culture and prescription datasets were used to train clinical prediction models to predict the probability of AMR and treatment outcomes respectively—the baseline characteristics of these populations, and the Emergency Department population used in the simulation study, are summarized in Table 1. Most urine specimens were obtained from females (71.8%, *n* = 17,343), and older patients (most represented decile 70–79 [19.3%, *n* = 4655]). Most prescriptions were for older patients (most represented decile 60–69 [20.7%, *n* = 17,926]). The three most prescribed antibiotics for all indications (summarized in Supplementary Table 1) were vancomycin (15.9%, *n* = 59,363), cefazolin (11.9%, *n* = 44,351), and ciprofloxacin (11.2%, *n* = 41,930). The commonest organism grown was *Escherichia coli*, accounting for more than half of urinary isolates. In the simulation study dataset, most patients had intermediate severity illness at Emergency Department triage — 2.7% (*n* = 9) scored four (lowest severity) and 10.4% (*n* = 35) scored one (highest severity). Antibiograms of the datasets used to train/validate the clinical prediction models and to perform the simulation study are displayed in Supplementary Fig. 1. Overall susceptibility rates were highest in *E. coli* and *Klebsiella pneumoniae*, which exhibited 9 and 10 percent rates of ceftriaxone resistance respectively. Overall susceptibility rates were lowest in *Enterococcus faecium* and non-speciated Enterococci, which were the only isolates with high rates of phenotypic resistance to piperacillin-tazobactam. Phenotypic meropenem resistance was observed in some *Pseudomonas* isolates, but was not observed in Enterobacterales. The commonest potential UTI symptoms were non-specific systemic symptoms, and where coded UTI diagnoses were made, the site was usually not specified.

**Table 1:** Baseline characteristics of the study population

Characteristic	Subtype	Prescription model n (%)	Urine model n (%)	Urine simulation n (%)
Gender	F	43,383 (50)	17,114 (71.9)	229 (68.2)
··	M	43,298 (50)	6697 (28.1)	107 (31.8)
Race	White	60,607 (69.9)	14,236 (59.8)	197 (58.6)
··	Black	9417 (10.9)	3174 (13.3)	55 (16.4)
··	Other	5028 (5.8)	1079 (4.5)	15 (4.5)
··	Unknown	4696 (5.4)	3264 (13.7)	49 (14.6)
··	Hispanic	3974 (4.6)	1239 (5.2)	12 (3.6)
··	Asian	2959 (3.4)	819 (3.4)	8 (2.4)
Age group	60–69	17,926 (20.7)	4368 (18.3)	56 (16.7)
··	50–59	15,302 (17.7)	3178 (13.3)	40 (11.9)
··	70–79	15,167 (17.5)	4580 (19.2)	75 (22.3)
··	80–89	11,355 (13.1)	4058 (17)	71 (21.1)
··	40–49	9510 (11)	1991 (8.4)	20 (6)
··	30–39	7147 (8.2)	1754 (7.4)	17 (5.1)
··	18–29	6718 (7.8)	2491 (10.5)	36 (10.7)
··	≥90	3556 (4.1)	1391 (5.8)	21 (6.2)
Marital status	Unmarried/unknown	47,065 (54.3)	15,221 (63.9)	223 (66.4)
··	Married	39,616 (45.7)	8590 (36.1)	113 (33.6)
Language spoken	English	77,942 (89.9)	18,457 (77.5)	254 (75.6)
··	Other/unknown	8739 (10.1)	5354 (22.5)	82 (24.4)
Insurance	Other/unknown	47,151 (54.4)	12,106 (50.8)	164 (48.8)
··	Medicare	33,185 (38.3)	10,051 (42.2)	152 (45.2)
··	Medicaid	6345 (7.3)	1654 (6.9)	20 (6)
Year group	2008–2010	31,426 (36.3)	10,348 (43.5)	87 (25.9)
··	2011–2013	20,678 (23.9)	5147 (21.6)	107 (31.8)
··	2014–2016	18,921 (21.8)	4465 (18.8)	71 (21.1)
··	2017–2019	15,656 (18.1)	3851 (16.2)	71 (21.1)
Antibiotic outcome	CDI	474 (0.5)	NA	NA
··	Toxicity	17,253 (19.9)	NA	NA
Ampicillin result	R	NA	12,618 (53)	183 (54.5)
··	S	NA	11,057 (46.4)	150 (44.6)
··	I	NA	136 (0.6)	3 (0.9)
Ampicillin/sulbactam result	S	NA	14,907 (62.6)	212 (63.1)
··	R	NA	6504 (27.3)	94 (28)
··	I	NA	2400 (10.1)	30 (8.9)
Piperacillin/tazobactam result	S	NA	22,468 (94.4)	318 (94.6)
··	R	NA	1195 (5)	17 (5.1)
··	I	NA	148 (0.6)	1 (0.3)
Cefazolin result	S	NA	13,789 (57.9)	210 (62.5)
··	R	NA	9885 (41.5)	126 (37.5)
··	I	NA	137 (0.6)	NA
Ceftriaxone result	S	NA	15,583 (65.4)	241 (71.7)
··	R	NA	8190 (34.4)	93 (27.7)
··	I	NA	38 (0.2)	2 (0.6)
Ceftazidime result	S	NA	16,943 (71.2)	267 (79.5)
··	R	NA	6663 (28)	64 (19)
··	I	NA	205 (0.9)	5 (1.5)
Cefepime result	S	NA	18,399 (77.3)	283 (84.2)
··	R	NA	5232 (22)	51 (15.2)
··	I	NA	180 (0.8)	2 (0.6)
Meropenem result	S	NA	19,409 (81.5)	295 (87.8)
··	NT	NA	4123 (17.3)	37 (11)
··	R	NA	220 (0.9)	2 (0.6)
··	I	NA	59 (0.2)	2 (0.6)
Ciprofloxacin result	S	NA	18,388 (77.2)	261 (77.7)
··	R	NA	5221 (21.9)	73 (21.7)
··	I	NA	202 (0.8)	2 (0.6)
Gentamicin result	S	NA	17,803 (74.8)	273 (81.2)
··	R	NA	5734 (24.1)	58 (17.3)
··	I	NA	274 (1.2)	5 (1.5)
Trimethoprim/sulfamethoxazole result	S	NA	14,346 (60.2)	216 (64.3)
··	R	NA	5226 (21.9)	82 (24.4)
··	NT	NA	4239 (17.8)	38 (11.3)
Nitrofurantoin result	S	NA	16,930 (71.1)	245 (72.9)
··	R	NA	4289 (18)	55 (16.4)
··	I	NA	2592 (10.9)	36 (10.7)
Vancomycin result	R	NA	20,490 (86.1)	304 (90.5)
··	S	NA	3321 (13.9)	32 (9.5)
Genus grown	*Escherichia*	NA	12,425 (52.2)	195 (58)
··	*Enterococcus*	NA	4239 (17.8)	38 (11.3)
··	*Klebsiella*	NA	3319 (13.9)	53 (15.8)
··	*Proteus*	NA	1541 (6.5)	17 (5.1)
··	*Pseudomonas*	NA	1020 (4.3)	20 (6)
··	*Enterobacter*	NA	584 (2.5)	7 (2.1)
··	*Citrobacter*	NA	287 (1.2)	4 (1.2)
··	*Morganella*	NA	183 (0.8)	2 (0.6)
··	*Serratia*	NA	172 (0.7)	0 (0)
··	*Providencia*	NA	30 (0.1)	0 (0)
··	Other	NA	12 (0.1)	0 (0)
Illness severity score	1	NA	NA	35 (10.4)
··	2	NA	NA	129 (38.4)
··	3	NA	NA	163 (48.5)
··	4	NA	NA	9 (2.7)
Potential UTI symptoms	Non-specific^a^	NA	NA	113 (33.6)
··	None	NA	NA	111 (33)
··	Abdominal pain	NA	NA	44 (13.1)
··	Dysuria	NA	NA	26 (7.7)
··	Flank or back pain	NA	NA	23 (6.8)
··	Haematuria	NA	NA	12 (3.6)
··	Frequency	NA	NA	5 (1.5)
··	Urinary retention	NA	NA	2 (0.6)
Coded UTI diagnoses	None	NA	NA	162 (48.2)
··	UTI (unspecified site)	NA	NA	149 (44.3)
··	Pyelonephritis	NA	NA	22 (6.5)
··	Cystitis	NA	NA	3 (0.9)
Antibiotic allergies	None	NA	NA	135 (40.2)
··	Unknown	NA	NA	127 (37.8)
··	Beta lactams	NA	NA	42 (12.5)
··	Sulfonamides	NA	NA	35 (10.4)
··	Macrolides	NA	NA	8 (2.4)
··	Vancomycin	NA	NA	6 (1.8)
··	Quinolones	NA	NA	5 (1.5)
··	Tetracyclines	NA	NA	3 (0.9)
··	Nitroimidazoles	NA	NA	1 (0.3)
··	Polymyxin B	NA	NA	1 (0.3)
Total	Patients	86,682 (100)	23,812 (100)	336 (100)

*CDI* *Clostridioides difficile* infection, *R* Resistant, *S* Susceptible, *I* Intermediate, *NT* Not tested. In MIMIC-IV, gender categories F and M are assumed to be female and male respectively.

^a^Fever, delirium, lethargy/weakness, fall, syncope/presyncope, hypotension, tachycardia, nausea, and/or vomiting.

Forty-nine UK-based prescribers (15 infectious diseases/medical microbiology [ID/MM], 11 haemato-oncology/respiratory medicine/acute medicine, nine urology/general surgery, nine intensive care [ICU], five General Practice [GP]) completed an online antibiotic choice ranking exercise in which they ranked the appropriateness of fictional antibiotics based on their characteristics (e.g., toxicity, Access/Watch category, availability of oral preparations—see Supplementary Fig. 2). Mean completion time was nine minutes, 12 s.

### Antibiotic decision-making algorithm structure

**Fig. 1:**
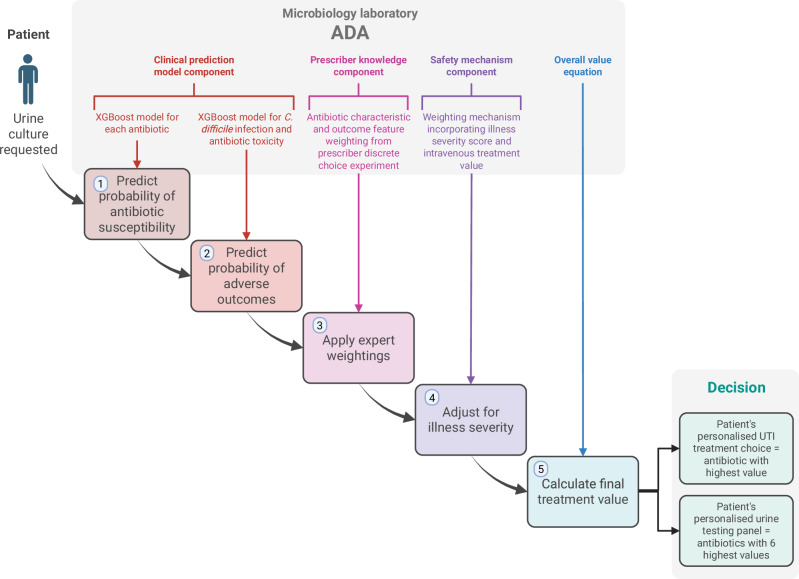
Structure of the antibiotic decision-making algorithm. Structure of the antibiotic decision-making algorithm (ADA)—the algorithm calculates probabilities of antibiotic treatment outcomes, weights them by importance to expert clinicians, then applies a safety mechanism for patients with severe illness. Created in BioRender. Howard, A. (2025) https://BioRender.com/pztvhdk.

We developed an antibiotic decision-making algorithm to encode a mathematical expression that weighted model predictions of urine pathogen antibiotic susceptibility and antibiotic treatment outcomes using the results of the prescriber antibiotic choice ranking exercise, as shown in Fig. 1. When a urine culture test is ordered for each patient, the algorithm makes personalized antibiotic treatment and testing decisions for that patient by calculating the relative treatment value (utility) of 13 antibiotic options, then choosing the antibiotic with highest value as treatment and the antibiotics with the top six highest values as an antibiotic susceptibility testing panel. Broadly, the algorithm determines treatment value by using a patient’s medical data to calculate the predicted probabilities of various antibiotic treatment outcomes with clinical prediction modeling, then weighting these probabilities according to their relative importance to expert clinicians.

### Clinical prediction model specification and performance

**Fig. 2:**
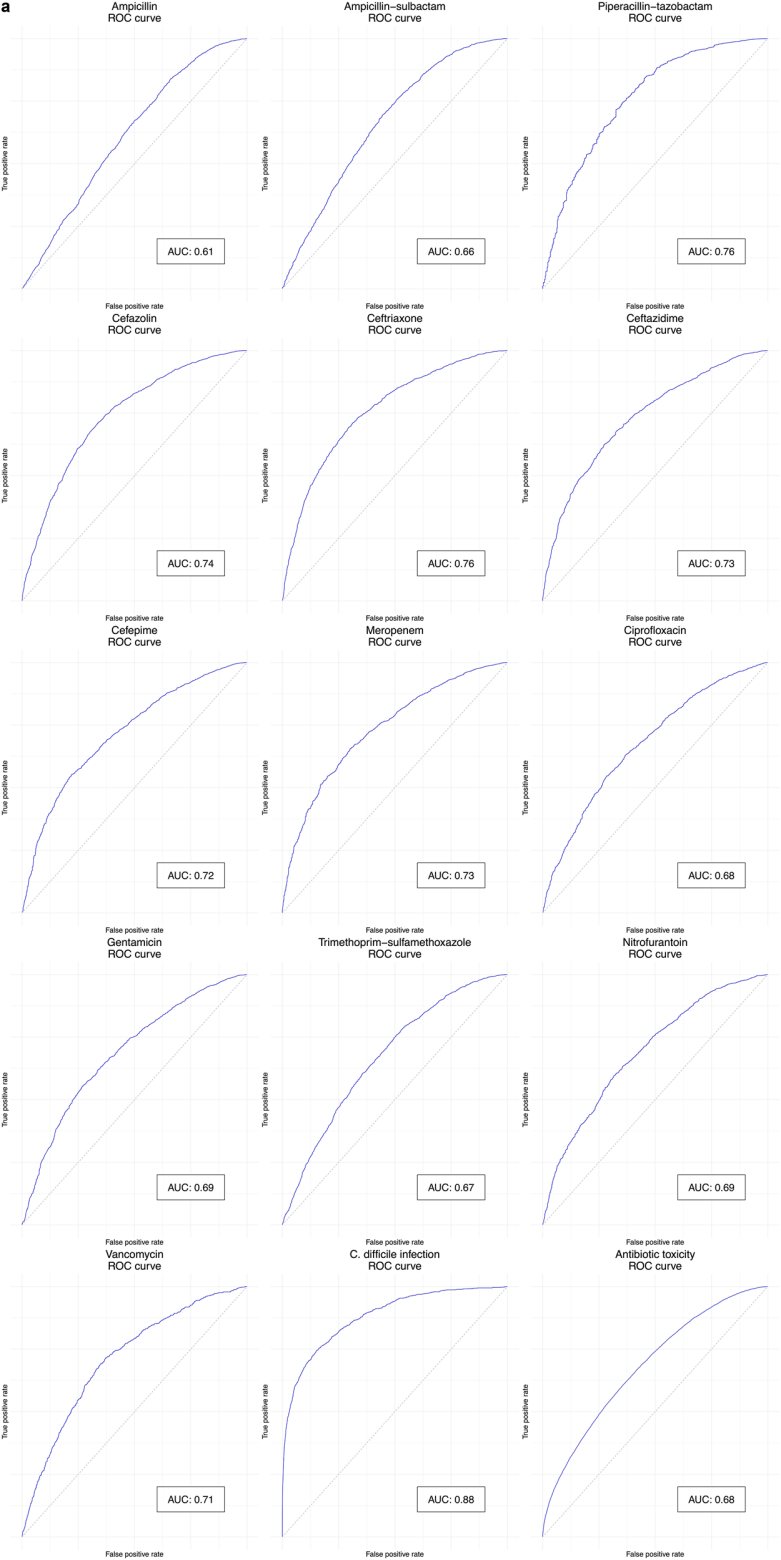

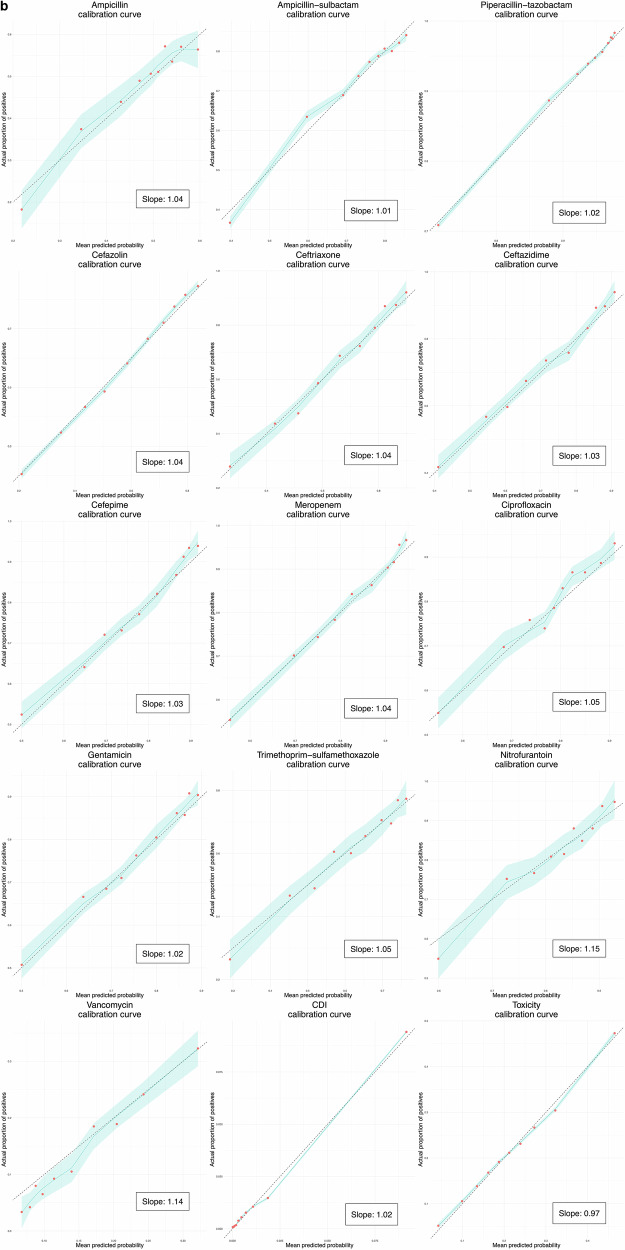
Receiver operating characteristic and calibration curves. Receiver operating characteristic curves (panel **a**) and calibration curves (panel **b**) for the 15 clinical prediction models. For receiver operating characteristic curves, blue lines represent the curve itself, the diagonal gray dashed line chance level (performance level if the model had no predictive value) and inset boxes the area under the curve. Calibration curves are represented by green lines, with green shaded area representing 95% confidence intervals, red dots representing means of probability groups, the slope coefficient of a linear model fitted to the points in inset boxes, and ideal calibration represented by gray dashed lines. CDI *Clostridioides difficile* infection.

In this study, the antibiotic decision-making algorithm incorporated probability predictions from 15 clinical prediction models—13 to predict the probability of urine pathogen susceptibility to the 13 antibiotics, one to predict the probability of *Clostridioides difficile* infection (CDI), and one to predict the probability of antibiotic toxicity (Fig. 1, steps 1 and 2). The training hyperparameters and predictor variable contributions of the gradient-boosted decision tree (XGBoost) models that were used are summarized in Supplementary Table 2 and Supplementary Fig. 3 respectively. Gender, age, previous hospital admission, presenting from the community, and previous antimicrobial treatment were most frequently among the top 10 contributors to predicting the antibiotic susceptibility of urine pathogens. Prior treatment with ceftriaxone, vancomycin, or the agent in question were particularly common strong predictors. Prior resistance to the agent in question often had predictive value, but was only in the top 10 predictors for ampicillin and ampicillin-sulbactam resistance. Prior urinary disease (International Classification of Diseases [ICD] 10 category N) contributed to prediction of ceftriaxone and trimethoprim-sulfamethoxazole susceptibility. Age and previous hospital admission were large contributors to predicting CDI and antibiotic toxicity. Receiver operating characteristic (ROC) curves/calibration curves and validation performance metrics (with a default decision threshold of 0.5 for classification metrics) for the 15 clinical prediction models are summarized in Fig. 2 and Supplementary Table 3 respectively. Seven models produced areas under ROC (AUROCs) between 0.7 and 0.8, six between 0.65 and 0.7. The CDI model had the highest AUROC, at 0.88 (95% confidence interval [CI] 0.869–0.891), and the model predicting the ampicillin susceptibility of urine pathogens had the lowest, at 0.613 (CI 0.597–0.63). The slope coefficients of all model calibration plots were between 0.9 and 1.1, except for those of nitrofurantoin (1.15) and vancomycin (1.14).

When the stability of clinical prediction models was assessed by progressively decreasing training dataset size (Supplementary Fig. 4), the *C. difficile* infection model had the largest difference in mean AUROC across six random train-test splits (0.772 with 14% training:86% testing, versus 0.661 with 2% training:98% testing). The vancomycin susceptibility prediction model had the largest AUROC standard deviation (0.023 with 2% training:98% testing). When clinical prediction models were tested out-of-sample by training on data from one time period and testing on data from other time periods across six random train-test splits (Supplementary Fig. 6), the *C. difficile* infection prediction model had the largest difference in mean AUROC between training and testing time groups (0.893 with 2008–2010 training:2017–2019 testing versus 0.607 with 2017–2019 training:2014–2016 testing) and the *C. difficile* infection prediction model also had the largest AUROC standard deviation (0.084 with 2011–2013 training:2011–2013 testing). When the fairness of clincial predictions models was assessed by measuring predictive performance in different demographic groups across six train-test splits (Supplementary Fig. 5), the largest differences in mean AUROC were observed when predicting the antibiotic susceptibility of urine culture isolates for different age groups (0.823 for piperacillin-tazobactam in 40–49 s versus 0.669 in 30–39 s), races (0.719 for ciprofloxacin in Hispanic patients versus 0.633 in the aggregate least-represented racial groups), and sexes (0.727 for meropenem in females versus 0.609 in males). The differences observed in gender and race may have been due to less data being available for the minority class (males) and heterogeneity in the aggregate least-represented racial groups respectively. The relatively poor performance in the 30–39 age group may be due to a paucity of positive features for model training due to relatively little healthcare exposure compared to older age groups, and a higher resistance rate than the 18–29 group. The largest AUROC standard deviations were observed when making predictions for ≥90 s (0.089 for piperacillin-tazobactam), Asian patients (0.078 for piperacillin-tazobactam), and males (0.022 for piperacillin-tazobactam).

### Expert weightings from the antibiotic choice ranking exercise

**Fig. 3:**
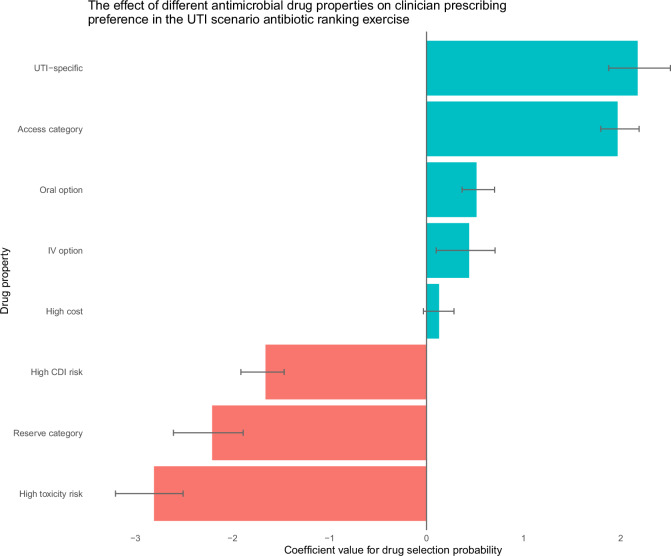
Expert weightings from the antibiotic choice ranking exercise. Expert weightings extracted from the antibiotic choice ranking exercise that was undertaken by clinicians. Bar color represents whether the characteristic increased (green) or decreased (red) the probability of antibiotic selection, and therefore how that characteristic was subsequently weighted in the antibiotic decison-making algorithm. Error bars represent 95% confidence intervals. CDI *Clostridioides difficile* infection.

The algorithm applies expert weightings (Fig. 1, step 3) to both model-predicted probabilities and known characteristics of the antibiotic (Access/Watch/Reserve [AWaRe] category, UTI-specificity, availability of oral preparation[s], availability of IV preparation[s], and cost)—in this study, these expert weightings were extracted from the overall results of the antibiotic choice ranking exercise undertaken by clinicians (i.e., across all specialties). The relative influences of different antibiotic characteristics on clinician antibiotic choices in the antibiotic choice ranking exercise are displayed in Fig. 3. UTI-specificity and toxicity had the largest positive and negative weight respectively when clinician participants selected antibiotics—they were therefore weighted most positively and negatively respectively by the algorithm when calculating antibiotic value. High antibiotic cost had the least influence on the probability of antibiotic selection, and therefore contributed little to antibiotic decisions made by the algorithm.

To demonstrate how the algorithm expert weightings can be adapted to different clinical settings, characteristic importances from the antibiotic choice ranking exercise are displayed stratified by clinician specialty in Supplementary Fig. 7. GPs and ID/MM specialists were more likely to select antibiotics with oral preparations than those with intravenous preparations. ICU and medical specialists were more likely to select antibiotics with intravenous preparations than those with oral preparations and most likely to select Access antibiotics. ICU specialists were least likely to select Reserve antibiotics. Wide confidence intervals in the GP sub-analysis (see Supplementary Fig. 7, bottom plot) reflect low respondent numbers (*n* = 5).

### Antibiotic treatment values calculated by the algorithm

**Fig. 4:**
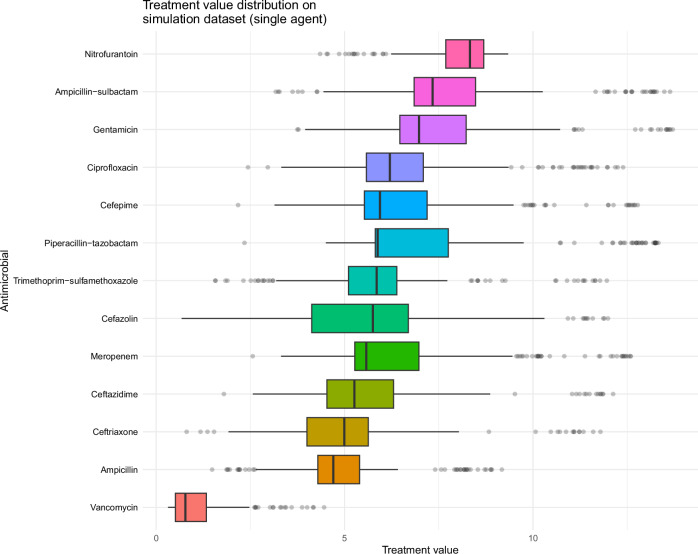
Antibiotic treatment value calculated by the algorithm in the simulation study population. Distributions of antibiotic treatment value calculated by the antibiotic decision-making algorithm for Emergency Department attendees in the simulation study dataset. The central line represents the median, the box the interquartile range, the whiskers 1.5 times the interquartile range beyond quartiles one and three, and the dots outliers.

The algorithm applies a safety mechanism adjustment (Fig. 1, step 4) to ensure that treatment efficacy is prioritized over other factors in patients with more severe infection—in this study, this was achieved by weighting probability of organism susceptibility to the antibiotic and IV administrability more heavily as patients became more severely unwell (measured using an illness severity score taken at Emergency Department triage). This produced a final treatment value (Fig. 1, step 5) for each antibiotic, that the algorithm used to rank the 13 antibiotic options and choose the patient’s personalized antibiotic treatment and susceptibility testing panel. The distributions of antibiotic treatment values calculated by the algorithm for patients across the simulation study dataset are displayed in Fig. 4. Nitrofurantoin had the highest median treatment value across the population (8.328, interquartile range [IQR] 1.007), and vancomycin the lowest (0.778, IQR 0.821). Cefazolin had the widest spread in treatment value (IQR 2.563), reflecting variation in probability of urine isolate susceptibility to cefazolin in the dataset (IQR 0.28).

The results of a subset analysis where treatment values were produced using the expert weights from each specialty in turn (see above and Supplementary Fig. 6) are displayed in Supplementary Fig. 8. Nitrofurantoin had the highest median treatment value across the population for all specialties apart from ICU and surgery, for which gentamicin had the highest median treatment value. The GP-weighted algorithm favored antibotics with oral preparations more than other specialties did, while the ICU-weighted algorithm favored intravenous antibiotics more than other specialties did.

### Simulation study in emergency department attendees

When the algorithm was used to choose an empirical antibiotic treatment for Emergency Department attendees at the point when they had urine cultures sent (in an assumed scenario where a decision to treat for UTI had already been made) the algorithm chose an agent that correctly targeted the urinary pathogen in as many cases as human prescribers did (83.6% of cases versus 79.5%, 95% confidence interval of difference −2% to 10.3%, *p* = 0.196). The algorithm chose more correctly-targeted antibiotics that were in the WHO Access category (75.6% of cases versus 11.9%, 95% confidence interval of difference 57.6% to 69.7%, *p* < 0.001) and had oral preparations (69% versus 22.6%, 95% confidence interval of difference 39.5 to 53.4%, *p* < 0.001) than human prescribers did, and fewer intravenously-administrable correctly-targeted antibiotics (31.2% versus 65.8%, 95% confidence interval of difference −41.9% to −27.1%, *p* < 0.001).

**Fig. 5:**
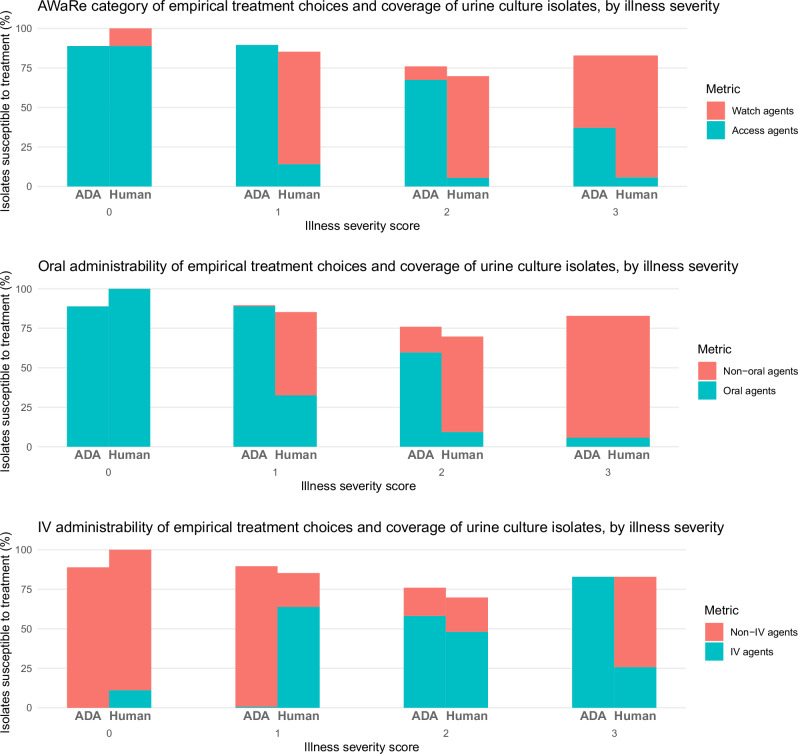
Proportion of correctly-targeted treatments stratified by illness severity. Plots comparing how increasing illness severity affected the success of empirical treatment choices made by the antibiotic decision-making algorithm (ADA) and human prescribers in correctly targeting patients’ urinary pathogens, by WHO Access/Watch/Reserve (AWaRe) category (top), availability of an oral option (middle), and availability of an IV option (bottom). The height of each colored bar section represents the number of cases in which an antibiotic choice from that category (e.g., Access agent) correctly targeted the urinary pathogen, as a proportion of the total number of choices made. Here, zero corresponds to the lowest illness severity and three the highest.

**Fig. 6:**
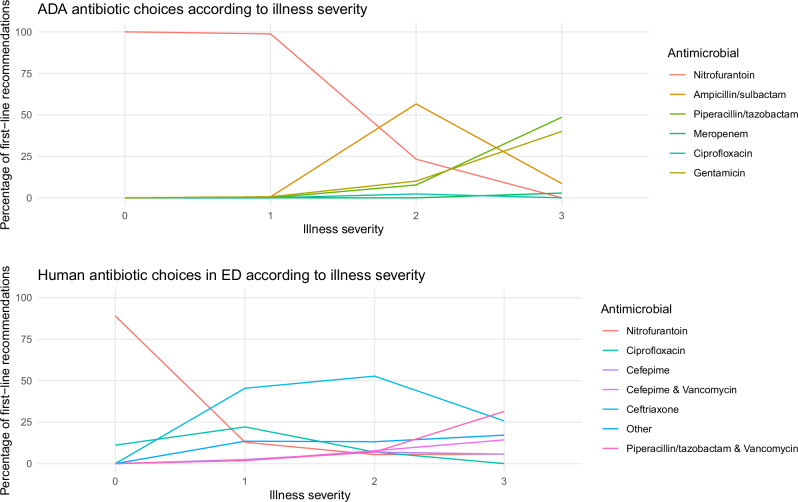
First-line treatment choices by antibiotic. The make-up of antibiotic decision-making algorithm first-line treatment recommendations by antibiotic agent depending on illness severity (with zero here corresponding to the lowest severity and three the highest). The top plot is for algorithmic recommendations, the bottom plot for antibiotics that were actually prescribed.

Figures 5 and 6 display how illness severity influenced the appropriateness and types of antibiotics chosen by the algorithm and human prescribers in the Emergency Department. The algorithm behaved in a similar way to human prescribers, in that it predominantly chose orally-administrable WHO Access antibiotics for the most systemically well patients (mainly nitrofurantoin) and intravenously-administrable WHO Watch antibiotics for the most systemically unwell patients (mainly piperacillin-tazobactam for the algorithm and piperacillin-tazobactam plus vancomycin for human prescribers). The algorithm, however, detected more opportunities to use correctly-targeted orally-administrable and Access category antibiotics in patients with intermediate illness severity while achieving higher rates of urinary pathogen coverage than human prescribers — it did this by making more use of nitrofurantoin and ampicillin-sulbactam. The algorithm also detected more opportunities to use correctly-targeted intravenous Access antibiotics in patients with high illness severity—it did this by making more use of gentamicin and avoiding the use of ceftriaxone. As shown in Supplementary Fig. 9, incorrectly-targeted treatments chosen by the algorithm were predominantly Access agents, and were predominantly orally-administrable except at the highest illness severity. Incorrectly-targeted human treatment choices were predominantly Watch agents and not orally-administrable. The proportions of incorrectly-targeted treatments that were intravenously-administrable were similar between algorithmic and human treatment choices.

An individualized antibiotic susceptibility testing panel composed of the top six algorithm choices (Supplementary Fig. 10) provided more susceptible results per specimen than a standard panel based on international treatment guidelines (median 6, 95% confidence interval of difference 0.5–1, *p* < 0.001, effect size [ES] 0.281), including more Access (median 3, 95% confidence interval of difference 1-1, *p* < 0.001, ES 0.456) and intravenous antibiotics (median 5, 95% confidence interval of difference 1-1, *p* < 0.001, ES 0.354) but similar numbers of oral antibiotics (median 2, 95% confidence interval of difference 0-0, *p* = 0.103, ES −0.077).

When the simulation study was repeated on the subset of patients with a coded UTI diagnosis and allergy information for that Emergency Department admission (*n* = 112) and the algorithm was adapted to avoid agents to which the patient was allergic, the main findings were the same as the main analysis in terms of their statistical significance, with the exception of the adaptive antimicrobial susceptibility testing panel providing slightly fewer orally-administrable options (*p* = 0.048). In this sub-population, the allergy-adaptive treatment algorithm improved upon its targeted management of the most severely ill patients, increasing pathogen coverage to 100% and utilizing a higher proportion of WHO Access agents in that cohort. The results of this subset analysis are displayed in Supplementary Fig. 11.

## Discussion

This study suggests that calculation of antibiotic treatment value using our algorithmic approach could contribute to antimicrobial stewardship programs that address UNGA AMR targets by improving the appropriateness of empirical antibiotic prescribing and susceptibility testing decisions for UTI. Human prescribers understand the importance of different antibiotic outcomes, but are less good at estimating the probability of these outcomes — our algorithmic approach addresses this problem by combining human judgment (expert weightings extracted from the antibiotic choice ranking exercise) with data-driven probability predictions (clinical prediction models)^10^. The algorithm also manages the tension between the need for individual patient treatment efficacy and the risk of patient harm/further AMR generation by incorporating a measure of illness severity—as the severity score increases, the algorithm recommends more intravenous and Watch category agents to maximize the probability of delivering a microbiologically active drug. Our results suggest that an algorithmic approach could help healthcare organizations increase the appropriate use of WHO Access category agents. Implementing decision support systems based on such an algorithm could therefore help countries meet the UN General Assembly target of 70% Access category antibiotic use by 2030.

Our value-based approach contrasts with existing work in antibiotic decision support, which have mainly either provided guideline-based recommendations, probability predictions without a recommendation nor an indication of the relative value of different options, or a treatment recommendation based on probabilistic prediction of a single factor (e.g., resistance to the chosen treatment)^11–13^. Where a value-based approach has been used in previous work, calculations of cost and benefit were made based on either probability prediction or literature evidence of mechanistically-intuitive factors such as 30-day mortality and ecological impact^14^. The drawback of this approach is that the relative importance of these factors are still hard-coded within the algorithm itself, while only their probabilities change at a local level. This limits the ability of such an approach to leverage the expertise and judgment of clinicians that will ultimately be using the tool. When stratified by specialty, the results of our prescriber antibiotic choice ranking exercise demonstrate that different clinicians have varying priorities that reflect the needs of their respective patient cohorts. Heterogeneity is also likely to arise from differences between healthcare settings (e.g., high versus low- and middle-income). The algorithm is therefore constructed to allow for different health systems and specialist groups (see Supplementary Figs. 6 and 7) to have their own locally-adapted algorithm via two mechanisms: firstly, the antibiotic choice ranking exercise can be undertaken by clinicians in the area it is to be used, ensuring expert weightings are appropriate to the local setting; and secondly, the safety mechanism can be calibrated to the local illness severity score of choice (e.g., APACHE-2 in ICU and National Early Warning Score [NEWS] in other inpatient wards). The recommendations could also account for antibiotic contraindications, as demonstrated by the sub-analysis where the algorithm adapted to prior allergy history. Available options could also be stratified by clinical diagnosis (e.g., not including nitrofurantoin if upper UTI is suspected). Digital infrastructure may be a barrier to using the algorithm for personalized (i.e., individual-level) recommendations in low- and middle-income countries, but not to population and policy-level decisions (e.g., antibiotic formularies and supply chains).

Robust, scalable implementation of decision support based on algorithmic approaches will require three key enablers: (1) local dataflows will need to be complete and timely enough to inform probability predictions at individual (if available) or population level, depending on the application; (2) a local hub will be required for the delivery, maintenance, and performance monitoring of decision support to clinicians—for individual decisions, we propose that diagnostic microbiology laboratories could form these hubs (see Fig. 1), given that they usually contain infection domain experts and the infrastructure to communicate results to clinicians; and (3) regulations concerning clinical decision support tools will need to allow algorithms to adapt to local populations—one of the main benefits of our algorithm is its ability to adapt to the priorities and needs of local clinicians and patients.

Our study has several limitations. Firstly, the urine dataset was limited to secondary care patients, the prescription dataset to inpatients, and the simulation study dataset to Emergency Department patients—the approach needs to be validated in primary care where most antibiotic prescriptions occur, and in patient cohorts that better represent those that were relatively under-represented in this dataset (e.g., some racial groups). The approach also requires validation in settings where the same data parameters used as predictors may not be available (for example, insurance status)—the feature importance analysis (Supplementary Fig. 3), however, suggests that the most important features were generalizable to a range of healthcare settings (e.g., previous antibiotic treatment). Secondly, in the main analysis we have not examined potential outcomes where organisms are not grown in urinary specimens, accounted for the patient’s clinical syndrome beyond illness severity score (i.e., asymptomatic bacteriuria, cystitis, or pyelonephritis) due to the unreliability of clinical coding data, and there were insufficient antibiotic contraindication data to detect barriers to recommendations. We have, however, demonstrated that the conclusions of the main analysis also applied when we adjusted the patient cohort to patients with coded UTIs and adjusted the algorithm to account for allergy history. Thirdly, for simplicity and to maximize the number of participants, overall antibiotic choice ranking exercise results across several specialties were used to inform expert weightings for the main simulation study analysis, meaning that weightings may been influenced by how well-represented each specialty was in the antibiotic choice ranking exercise (for example, there were few GP respondents)—further work is required to understand how best to engage busy clinicians in generating these vital qualitative and quantitative data for expert weightings. Lastly, the algorithm cannot detect scenarios in which risks of treatment outweigh benefits (i.e., negative value). This approach was chosen because there was insufficient data to reliably inform decisions to decide whether or not to treat—use of the algorithm therefore makes the assumption that treatment is indicated, and facilitates the choice of agent in that event. However, it is possible for an agent to have zero utility, and the universally low vancomycin treatment value rankings in this study (see Fig. 4) show that it can agnostically inform decisions to avoid less suitable antibiotics where plausible alternatives are available. Vancomycin was, however, the most commonly used agent in the prescription dataset, most likely due to its use in indications other than UTI. This is unlikely to have significantly biased utility calculations because clinical covariates included in prediction models help to mitigate bias resulting from confounders related to the nature of the drug’s use (e.g., comorbidities related both to infection diagnosis and probability of CDI/toxicity). A UTI-specific prescription dataset could help provide further mitigation for such confounders, but this would be at the expense of training data volume due to the likely paucity of agents seldom used for UTI in that dataset.

Despite these limitations, our study demonstrates how an algorithm that quantifies the key considerations of antibiotic prescribing decisions (namely the probability and importance of different outcomes, and the consequences of ineffective treatment) could help to improve the appropriateness of antibiotic prescribing through appropriate prioritization of Access and orally-administrable antibiotics. Most importantly, it could do this in a way that preserves clinical efficacy where it matters most—in severely ill patients at high risk of deterioration from sepsis. Further studies of this approach are needed across a diversity of populations and care settings to better understand the impact of utility-based antibiotic decision support on individual and population outcomes.

## Methods

### Data sources and processing

The study used four datasets:

1. Urine culture data to train clinical prediction models to predict the probability of antibiotic susceptibility of patients’ urine culture isolates using their prior healthcare data.
2. Antibiotic prescription data to train clinical prediction models to predict probability of *Clostridioides difficile* infection (CDI) and drug toxicity following antibiotic treatment.
3. Another set of urine culture data from Emergency Department patients to perform a simulation study comparing algorithm antibiotic recommendations against antibiotics prescribed by human clinicians in the retrospective data.
4. Antibiotic ranking data from clinicians who participated in a UTI scenario-based antibiotic choice ranking exercise.

The clinical prediction models and the simulation study used data from PhysioNet MIMIC-IV/MIMIC-IV-ED/MIMIC-IV-Note version 2.2, which are open-source, pseudonymized electronic healthcare record datasets for inpatients and outpatients over the age of 18 admitted to Beth Israel Deaconess Medical Center (Boston, MA) intensive care (ICU) or Emergency Department between 2008 and 2019 (https://physionet.org/content/mimiciv/2.2/)^9^. Data preprocessing and quality checking (Supplementary Fig. 12) were performed using R v4.3.2 (2023-10-31) in a similar way to our recent work in personalized antimicrobial susceptibility testing^15^.

Following preprocessing, prescribing and urine culture susceptibility data were available for 13 antibiotics—six WHO Access category agents (ampicillin, ampicillin-sulbactam, cefazolin, gentamicin, trimethoprim-sulfamethoxazole, and nitrofurantoin) and seven Watch category agents (piperacillin-tazobactam, ceftriaxone, ceftazidime, cefepime, meropenem, ciprofloxacin, and vancomycin). Clinical prediction models were developed for both these individual agents and common two-agent combinations (e.g., piperacillin-tazobactam and vancomycin administered together), but for simplicity and to limit computation time, the final results reported in this paper are only for single-agent treatments. The size of the dataset was deemed to have sufficient case-variable ratio to minimize the risk of overfitting^16^. Data for the simulation study consisted of positive urine cultures from Emergency Department patients that were taken on the same day that they were triaged and received at least one of the 13 antibiotics listed above. Illness severity was an ‘acuity’ variable ranging from four (mild) to one (severe) - this was converted to a zero (mild) to three (severe) scale to facilitate the algorithm’s safety mechanism (see below).

Antibiotic cost was determined using each drug’s lowest U.S. Department of Veterans Affairs National Acquisition Center procurement price (November 2024)—U.S. dollar values were divided by the highest value (including two-antibiotic combinations) to produce normalized numbers between zero and one^17^.

The antibiotic choice ranking exercise was a UTI scenario-based task (Supplementary Fig. 2) that was conducted online (https://www.surveymonkey.com) between 1st June and October 31st 2024 by UK-based clinicians in general practice (GP), medicine, surgery, ICU, and infectious diseases/medical microbiology (ID/MM), recruited via organizational and departmental email single points of contact. There was no formal sample size calculation—the primary aim was to have at least one representative of each specialty. Participants ranked a set of fictional antibiotics in order of their suitability for managing UTI in their usual clinical setting based on drug characteristics. The set of options was limited to 13 antibiotics with six characteristics (WHO Access/Watch/Reserve [AWaRe] class, CDI risk, toxicity, UTI-specificity, availability of oral and IV preparations) to minimize the time taken for participants to complete the exercise^18^. The antibiotics and their characteristics were fictional (rather than using the 13 antibiotics used in the study) to provide the widest variation in characteristics, and ensure that participants based their ranking purely on the antibiotic characteristics provided.

### Antibiotic decision-making algorithm

The algorithm calculates value (otherwise known as utility, $U$), for each antibiotic option in each patient by incorporating the characteristics and consequences of antibiotic treatments, the importance of those characteristics/consequences, and a safety mechanism for patients with severe infection. The characteristics and consequences are chosen to reflect individual (e.g., drug toxicity) and population (e.g., AWaRe class) considerations.

The predicted consequences of antibiotic treatment were expressed as probabilistic predictions made, in this case, using gradient-boosted decision tree (XGBoost) clinical prediction models. Full tuning, training, and validation methodologies for these models are reported in the next section in accordance with the TRIPOD + AI reporting framework^19^.

By design, the minimum possible value of $U$ is zero because the algorithm chooses between antibiotics rather than deciding whether to treat (in which case a negative value would mean that the risks of treatment outweigh the benefits). We propose that $U$, for antibiotic $x$ in patient $n$, is

1 $${U}^{\left(x,n\right)}={p}_{s}^{\left(x,n\right)}\left({O}^{\left(x,n\right)}+{C}^{\left(x\right)}+{S}^{\left(x,n\right)}\right)$$

where ${p}_{s}^{\left(x,n\right)}$ is the probability of the *n*th patient’s urinary pathogen being susceptible to antibiotic $x$, and where ${O}^{\left(x,n\right)}$, ${C}^{\left(x\right)}$, and ${S}^{\left(x,n\right)}$ relate to the predicted outcomes of antibiotic treatment, antibiotic characteristics, and the safety mechanism respectively. We note that $C$ does not depend on $n$ (patient characteristics) because it is defined solely by antibiotic characteristics. If ${p}_{s}^{\left(x,n\right)}=0$, then ${U}^{\left(x,n\right)}=0$, i.e., the value of antibiotic $x$ is zero if the probability of the patient’s urinary pathogen being susceptible to that antibiotic is zero. In the other extreme case, i.e., ${p}_{s}^{\left(x,n\right)}=1$, variation in ${U}^{\left(x,n\right)}$ is determined entirely by variation in ${O}^{\left(x,n\right)}$, ${C}^{\left(x\right)}$, and/or ${S}^{\left(x,n\right)}$. Before defining ${O}^{\left(x,n\right)}$, ${C}^{\left(x,n\right)}$, and ${S}^{\left(x,n\right)}$, we must define the function

2 $$u\left(w,k\right)=\left\{\begin{array}{lc}{wk} & {\mathrm{if}}\,w\ge 0\\ \left|w\right|\left(1-k\right) & \mathrm{otherwise}\end{array}\right.$$

where $k$ are either known antibiotic characteristics or outcome probabilities (estimated using clinical prediction models—see below). Expert weightings, $w$, represent the importance of antibiotic characteristics and outcomes to prescribers. These expert weightings were calculated by constructing a ranked logit model on data from the results of the antibiotic choice ranking exercise undertaken by clinicians—the full methodology for the ranked logit model is summarized below.

**Fig. 7:**
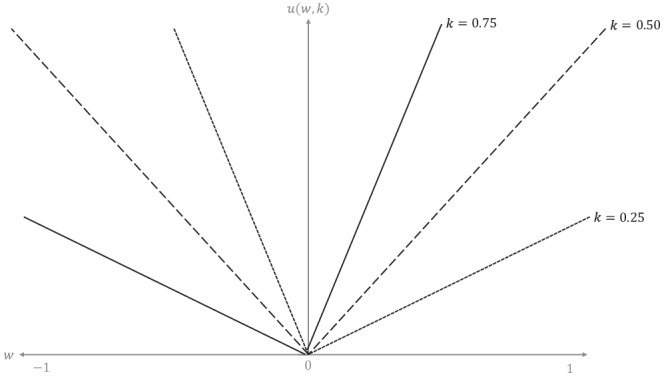
Function for extracting value from positive and negative expert weightings. The effect of variation in *w* on the value of *u(w,k)* for three different values of *k* represented by the solid, dotted, and dashed lines.

If $w$ is negative, (i.e., an outcome or characteristic that was undesirable to prescribers in the antibiotic choice ranking exercise), an increase in $k$ reduces the value of $u\left(w,k\right)$, and vice versa (see Fig. 7).

The value of the predicted antibiotic outcomes, ${O}^{\left(x,n\right)}$, is defined as

3 $${O}^{\left(x,n\right)}=u\left({w}_{c},{p}_{c}^{\left(x,n\right)}\right)+u\left({w}_{t},{p}_{t}^{\left(x,n\right)}\right)$$

where ${p}_{c}^{\left(x,n\right)}$ and ${p}_{t}^{\left(x,n\right)}$ are the patient’s estimated probability of CDI and toxicity following antibiotic treatment respectively, and ${w}_{c}$ and ${w}_{t}$ are the respective expert weightings from the prescriber antibiotic choice ranking exercise.

The value of known antibiotic characteristics, ${C}^{\left(x\right)}$, is

4 $${C}^{\left(x\right)}=u\left({w}_{u},{v}_{u}^{\left(x\right)}\right)+u\left({w}_{a},{v}_{a}^{\left(x\right)}\right)+u\left({w}_{o},{v}_{o}^{\left(x\right)}\right)+u\left({w}_{r},{v}_{r}^{\left(x\right)}\right)+u\left({w}_{h},{v}_{h}^{\left(x\right)}\right)$$

where ${v}_{u}^{\left(x\right)}$, ${v}_{a}^{\left(x\right)}$, ${v}_{o}^{\left(x\right)}$, and ${v}_{r}^{\left(x\right)}$ are one or zero to represent the presence or absence of UTI-specificity, Access category, oral administration option, and Reserve category respectively, ${v}_{h}^{\left(x\right)}$ is normalized financial cost, and ${w}_{u}$, ${w}_{a}$, ${w}_{o}$, ${w}_{r}$, and ${w}_{h}$ are the respective expert weightings from the prescriber antibiotic choice ranking exercise.

The safety mechanism for patients with severe infection, ${S}^{\left(x,n\right)}$, is

5 $${S}^{\left(x,n\right)}=u\left({w}_{i},{v}_{i}^{\left(x\right)}\right)\exp \left({a}^{\left(n\right)}b\right)$$

where ${v}_{i}^{\left(x\right)}$ is zero or one to represent the presence or absence of an intravenous administration option, ${w}_{i}$ is the respective expert weighting from the antibiotic choice ranking exercise, ${a}^{\left(n\right)}$ is an illness severity score chosen by the user (e.g., National Early Warning Score [NEWS]), and $b$ enables the user to calibrate to the scale of the chosen score (for example, NEWS ranges from 0-20 while APACHE-2 ranges from 0 to 71). In this study, the default $b=1$ was used, and $a$ was an Emergency Department illness acuity score measured on a scale of zero (least severe) to three (most severe). The minimum illness severity score observed in a population must be zero (in which case exp$\left({a}^{\left(n\right)}b\right)=1$). As illness severity score increases, patients are more likely to have severe infection (e.g., sepsis), in which case efficacy should be prioritized—increasing $a$ therefore exponentially increases the relative value of intravenous treatment $u({w}_{i},{v}_{i}^{\left(x\right)})$ and the antibiotic being active against the urinary pathogen ${p}_{s}^{\left(x,n\right)}$ (reflecting an exponential increase in the importance of consequences between full recovery and death)^20^.

### Clinical prediction model training and validation

A total of 15 individual models were required for the primary analysis. 13 of these models were individual antibiotic susceptibility prediction models that predicted probability of antibiotic activity against the urinary pathogen (i.e., pathogen susceptibility to the antibiotic), and were trained on the MIMIC-IV urine culture dataset. A *Clostridioides difficile* infection (CDI) prediction model and an antibiotic toxicity prediction model were trained on the MIMIC-IV prescriptions dataset.

The outcome for all antibiotic susceptibility prediction models was the probability of an ‘S’ result indicating susceptibility of the organism grown in that urine specimen to that antibiotic. The outcome for the CDI prediction model was the probability of a positive *C. difficile* stool result within three months following the start date/time of an antibiotic. The outcome for the antibiotic toxicity prediction model was the probability of a composite outcome of either stage three acute kidney injury (a new increase in serum creatinine to at least three times baseline or at least 3.54 µmol/dL in the absence of co-administration of nephrotoxic drugs as defined by the British National Formulary or intravenous contrast during the associated hospital admission), deranged liver function tests (a result newly above the upper end of the normal range for alanine aminotransferase, aspartate aminotransferase, or alkaline phosphatase in the absence of previous coded chronic liver disease or biliary instrumentation in the associated hospital admission), marrow suppression (new anaemia, leukopenia, or thrombocytopenia in the absence of co-administration of cytotoxic drugs as defined by the British National Formulary or coded bleeding diagnosis in the associated hospital admission) in the seven days following the start date of an antibiotic, or a coded antibiotic adverse event for the associated hospital admission^21,22,23^.

Choices of predictor variables and their time horizons were based on indirect or direct causal plausibility and/or association with outcome variables—this process was undertaken by the lead author (Consultant in Medical Microbiology, male, 30s, white British) and reviewed by co-authors (all male in the age range 20–60 with a racial mix of white British, White Australian, Maltese, Indian, and Chinese). Outcome and predictor variables were selected consistently across sociodemographic groups. No blinding to allocation or predictor/outcome assessment was implemented at any stage. Allocation to training and validation datasets was performed individually for each model by random 80:20 split without replacement, stratified to maintain similar proportions of the outcome in the training and validation datasets.

We have described clinical prediction models of resistance for 12 of the 13 antimicrobial agents using logistic regression in our previous work^24^. In an attempt to capture more non-linear relationships in the data, XGBoost (an ensemble method that improves predictive accuracy by sequentially fitting decision trees in so-called boosting rounds and combining their predictions, implemented via the ‘xgboost’ package https://cran.r-project.org/web/packages/xgboost/index.html), was used for this study^25,26^. XGBoost models underwent hyperparameter tuning and training on the training datasets using L2 regularization (ridge penalty) to control overfitting. Area under the receiver operating characteristic curve (AUROC) was used as the model evaluation metric. Class imbalance methods (e.g., class weighting) were not used.

Hyperparameter tuning was performed sequentially using AUROC for each of the 15 models across hyperparameters in three stages to reduce the number of hyperparameter combinations, and therefore computational time:

1. Ten maximum tree depth and minimum child weight values in the range two to nine and one to 10 respectively were selected using Latin hypercube sampling (LHS) with ‘randomLHS’ from the ‘lhs’ package (https://cran.r-project.org/web/packages/lhs/index.html) and tested using 5-fold cross validation across 50 boosting rounds (learning rate 0.05, subsample row ratio 0.8, subsample columns ratio 0.8).
2. Ten subsample row and column ratio values in the range 0.5–1 were selected and tested using the same method as above.
3. To balance computational time with performance, learning rates were tested by starting at a value of 0.1 then halving them or adding 0.1 as required (up to a maximum of 0.3) until the number of boosting rounds at which AUROC had not improved for 50 rounds was between 300 and 1000. If models did not converge within 1000 boosting rounds at a learning rate of 0.3, the number of required rounds required without improvement was reduced to 10, and maximum tree depth lowered sequentially from 6 by one until convergence occurred within 2000 rounds.

Predictor variable contributions to predictive value (feature importances) in model training were measured using Shapley additive explanation (SHAP) values with ‘predict’ from the ‘stats’ package^27^. Predictor variables with SHAP values of zero across the training dataset were then excluded when training the final model.

A single validation run was performed with the final trained model on the validation dataset, measuring AUROC, accuracy, precision, recall, and F1 score (decision threshold 0.5), with 95% confidence intervals approximated using bootstrapping with 1000 iterations. Calibration curves were plotted by separating probability predictions into ten groups of equal size and plotting group means against the actual prevalence of the outcome, calculating the slope coefficient of a linear model fitted to the points, and then plotting the curve using locally estimated scatterplot smoothing (LOESS) with shaded 95% confidence intervals.

A model stability analysis was performed to assess the performance of each model when trained on a smaller training dataset. Training and validation of the final model was performed in six random train-test splits without replacement (stratified by outcome) for each of four smaller train-test dataset size ratios (2:98%, 6:94%, 10:90%, and 14:86%)—AUROC, accuracy, precision, recall, and F1 score (decision threshold 0.5) were measured for each of these validations, and metric distributions were plotted using dot plots to assess heterogeneity in performance.

A model fairness analysis was performed where each of the same 15 trained models was validated separately across a range of protected characteristics (race, age, marital status, first language, and gender), with six random-train-test splits per characteristic—AUROC, accuracy, precision, recall, and F1 score (decision threshold 0.5) were again measured for each of these validation experiments, and metric distributions plotted using dot plots. Threshold recalibration for fairness was not performed because the output of the models were class probabilities rather than classifications.

A time cluster analysis was performed to assess out-of-sample performance, where the 15 models were trained on one of four time periods (2008–2010, 2011–2013, 2014–16, and 2017–2019), then validated on holdout datasets from that period and the other three time periods, measuring AUROC, accuracy, precision, recall, and F1 score (decision threshold 0.5)—this was repeated with six random train-test splits for each pair of time periods, and metric distributions were plotted using dot plots.

### Extracting expert weightings from the antibiotic choice ranking exercise

For the antibiotic ranking experiment to produce expert weightings ($w$), the ranking of each fictional antibiotic (1–13) by participants was recorded and converted to a long format where each participant’s chosen rank order of antibiotics was treated as a series of choices using the ‘mlogit.data’ method from the ‘mlogit’ package^28^. A multinomial ranked logit model was trained on the data to determine the relative importance of each of the six characteristics in influencing participant antibiotic rankings (again using ‘mlogit’). The model was trained to estimate maximum log-likelihood using the Broyden–Fletcher–Goldfarb–Shanno (BFGS) method^29^. 95% confidence intervals for characteristic importances were approximated using a bootstrap method with 1000 iterations. To demonstrate how the algorithm could be adapted to the needs of different clinical specialties, a subset analysis was performed where the approach was repeated on subsets of the data to build separate models for ID/MM, medicine, surgery, ICU, and GP to compare the importance of the six antimicrobial characteristics to different specialties.

### Simulation study

An individual-level simulation study evaluated the appropriateness of empirical algorithm treatment and testing decisions using retrospective real-world data^30^. Each patient who had a urine specimen sent from the Emergency Department had a treatment value calculated for all 13 antibiotics, which were then used to rank the antibiotics—the highest-ranked antibiotic became that patient’s simulated UTI empirical treatment recommendation, and the top six antibiotics became their simulated antibiotic susceptibility testing panel. For every patient, the algorithm therefore assumed that a decision to treat for UTI had already been made, and therefore only directed the choice of antibiotic agent. The two outcomes of interest were:

1. The appropriateness of algorithm treatment decisions, compared to human prescriber decisions—this was measured by the proportion of instances in which a urine culture isolate was susceptible to the chosen empirical antibiotic treatment (i.e., instances in which treatment was correctly-targeted), and the proportion of instances in which these were WHO Access category, orally-administrable, and intravenously-administrable (differences assessed by chi-squared test with 5% significance threshold). The way recommendations changed in response to increasing illness severity was assessed by bar plots.
2. The appropriateness of algorithm antibiotic susceptibility testing decisions, compared to an international guideline-based standard panel (nitrofurantoin, trimethoprim-sulfamethoxazole, gentamicin, piperacillin-tazobactam, ceftriaxone, and ciprofloxacin) used in our previous work in personalized antibiotic susceptibility testing^31^ — this was measured by the number of susceptible results per testing panel, and the number of these results that were for WHO Access category, orally-administrable, and intravenously-administrable antibiotics (differences assessed using a Wilcoxon signed ranks test with 5% significance threshold and effect size [test Z statistic divided by the square root of the total number of specimens]).

A sub-analysis was performed where the simulation study was repeated only on patients with UTI diagnoses coded for that Emergency Department admission, and who had previous allergy data available (in discharge summaries). The algorithm was also adapted in this sub-analysis to avoid agents to which the patient had a documented allergy, by working down the utility rankings until it found an agent to which the patient was not allergic.

### Ethics

The study complied with the PhysioNet MIMIC-IV (Medical Information Mart for Intensive Care) dataset Data Use Agreement 1.5.0. A UK Research Ethics Committee (IRAS 330186) application process determined that ethics committee review was not required.

## Supplementary information

Supplementary Information

## Data Availability

The MIMIC-IV version 2.2 data set is publicly accessible as a credentialed PhysioNet user at https://physionet.org/content/mimiciv/2.2/ once mandated training is completed, and the data use agreement is signed. Additional aggregate-level data can be provided by the authors if requests to do so are in line with legal and ethical data use regulations. Open-source code to reproduce the study using the above dataset is available at https://github.com/amh312/Antimicrobial_utility.
