# Supplementary material for: Algorithmic antibiotic decision-making in urinary tract infection using prescriber-informed prediction of treatment utility
Source: NPJ Digit Med. 2026 Jan 26;9:136. doi: 10.1038/s41746-026-02369-z (PMC12882935; doi:10.1038/s41746-026-02369-z)
Supplement: Supplementary file 1 — Supplementary Information [file 41746_2026_2369_MOESM1_ESM.pdf]

Supplementary information

Supplementary Table 1: Antibiotic courses in the prescription dataset

Antibiotic(s)	n (%)
Vancomycin	59363 (15.9)
Cefazolin	44351 (11.9)
Ciprofloxacin	41930 (11.2)
Ceftriaxone	31496 (8.4)
Cefepime & Vancomycin	29304 (7.8)
Piperacillin/tazobactam & Vancomycin	20124 (5.4)
Cefepime	20010 (5.4)
Trimethoprim/sulfamethoxazole	19452 (5.2)
Piperacillin/tazobactam	18643 (5)
Ciprofloxacin & Vancomycin	13300 (3.6)
Ampicillin/sulbactam	9849 (2.6)
Ceftriaxone & Vancomycin	9819 (2.6)
Meropenem	8917 (2.4)
Meropenem & Vancomycin	7337 (2)
Cefazolin & Vancomycin	6276 (1.7)
Other	4692 (1.3)
Ceftazidime & Vancomycin	4680 (1.3)
Ceftazidime	3253 (0.9)
Ampicillin/sulbactam & Vancomycin	2944 (0.8)
Trimethoprim/sulfamethoxazole & Vancomycin	2635 (0.7)
Ampicillin	2488 (0.7)
Nitrofurantoin	2283 (0.6)
Gentamicin	2207 (0.6)
Ciprofloxacin & Trimethoprim/sulfamethoxazole	1524 (0.4)
Ampicillin & Gentamicin	1387 (0.4)
Gentamicin & Vancomycin	1360 (0.4)
Cefepime & Trimethoprim/sulfamethoxazole	1251 (0.3)
Ceftriaxone & Trimethoprim/sulfamethoxazole	1086 (0.3)
Cefazolin & Ciprofloxacin	955 (0.3)
Cefazolin & Trimethoprim/sulfamethoxazole	945 (0.3)

Supplementary Fig. 1: Dataset urinary antibiograms

Antibiograms of organisms in the clinical prediction model training/validation dataset (top) and the simulation study dataset. Colour represents percentage of isolates susceptible to the relevant antibiotic (%S), ranging from zero (red) to 100 (green). In each cell, percentage susceptibility is listed followed by 95% confidence interval (calculated by Clopper-Pearson method using the antibiogram() function within the R 'AMR' package) and denominator N.

Organisms isolated on fewer than 10 occasions are not included. NOS = Not otherwise specified.

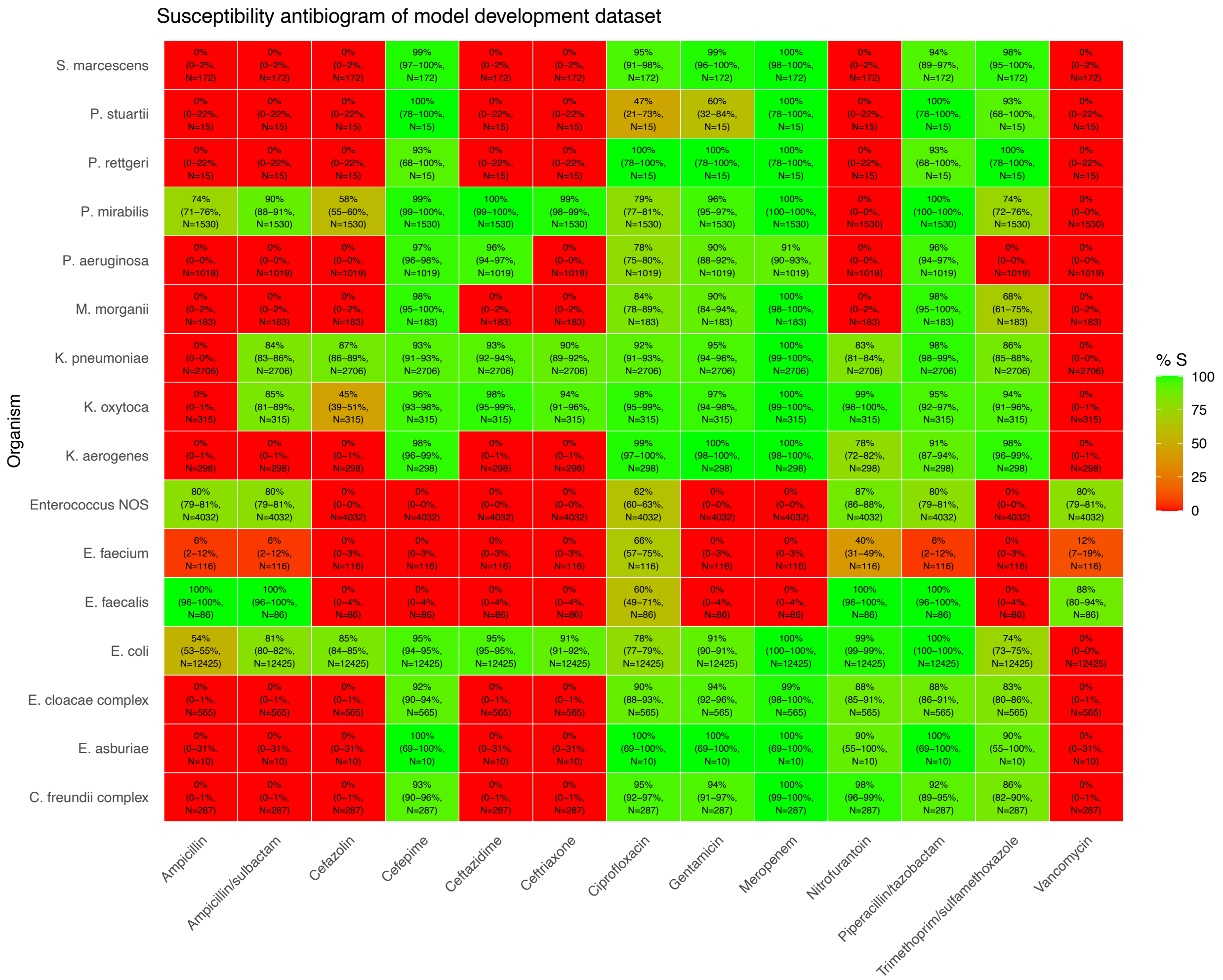

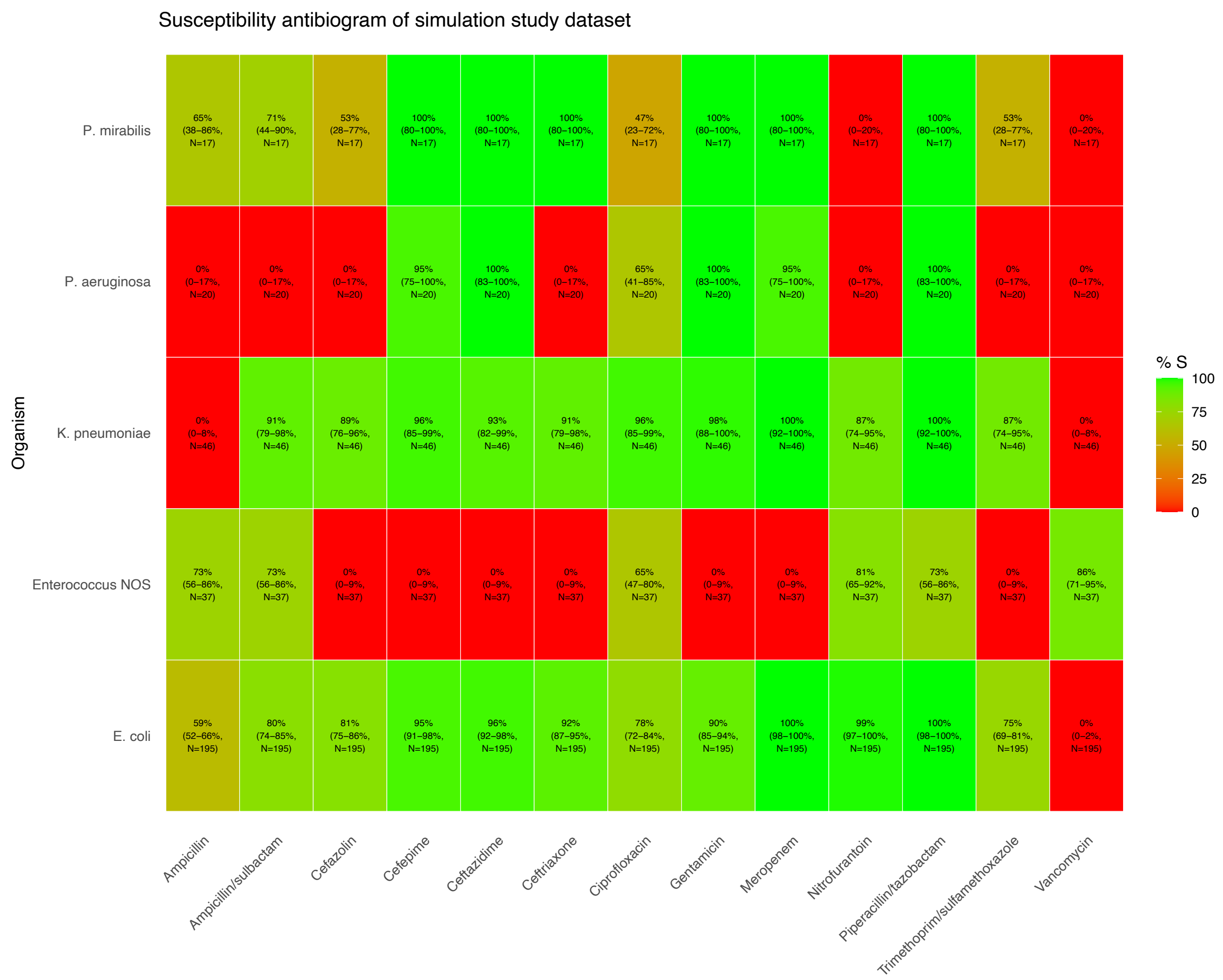

Supplementary Fig. 2: Antibiotic choice ranking exercise

This survey is designed to ascertain the relative importance of different features of an antimicrobial to clinicians when prescribing. It should take 2-5 minutes to complete. Contextual information about the patient has intentionally been omitted - your decision should be made based on your experience of managing urinary tract infection in your usual clinical setting. The factors listed are AWaRe classification*, risk of Clostridioides difficile diarrhoea, toxicity risk (e.g., nephrotoxicity), UTI-specificity (whether the drug should only be used for UTI), oral option, intravenous option, and financial cost.

- The WHO AWaRe classification assigns antibiotics ratings of Access, Watch and Reserve based on their risk of generating antimicrobial resistance. Those in the Access category are the lowest risk, those in Reserve are the highest.

1. A patient has urinary tract infection. You have no other details about the patient at this stage (including disease severity). Please rank the following fictional antibiotic treatments from 1 to 13 in order of most to least preferred in this scenario by

dragging and dropping options or using the arrows on the right. When finished, just click the ‘Done’ button at the bottom of the page.

	AWaRe class*	*C difficile* risk	Toxicity risk	UTI specificity	Oral option	IV option	Cost
**Abelfenide**	Reserve	Low	Low	No	Yes	Yes	High
**Acetemran**	Watch	Low	Low	Yes	Yes	No	Low
**Adenomadin**	Access	High	Low	No	Yes	Yes	Low
**Adrevenac**	Watch	High	Low	No	No	Yes	High
**Amrodine**	Reserve	High	Low	No	No	Yes	High
**Choriotroban**	Access	Low	High	No	No	Yes	Low
**Cormide**	Watch	Low	Low	No	No	Yes	High
**Decloxone**	Watch	Low	High	No	No	Yes	High
**Dexaset**	Access	Low	Low	No	Yes	Yes	Low
**Endoxolol**	Access	Low	Low	Yes	Yes	No	Low
**Olanzasys**	Watch	High	Low	No	No	Yes	Low
**Pansolid**	Access	Low	Low	No	Yes	No	Low
**Protestryl**	Watch	High	Low	No	Yes	Yes	Low

Supplementary Table 2: Final training hyperparameters used for the clinical prediction models

Model	Learning rate	Maximum tree depth	Minimum child weight	Row subsample	Column subsample	N training rounds
Ampicillin	0.0125	7	9	0.55	0.70	285
Ampicillin/sulbactam	0.0125	5	1	0.55	0.70	426
Piperacillin/tazobactam	0.0125	6	2	0.82	0.89	429
Cefazolin	0.0125	5	1	0.82	0.89	655
Ceftriaxone	0.0250	5	1	0.82	0.89	319
Ceftazidime	0.0125	6	8	0.74	0.64	401
Cefepime	0.0125	6	2	0.80	0.92	282
Meropenem	0.0125	7	9	0.93	0.50	454
Ciprofloxacin	0.0125	7	9	0.66	0.77	377
Gentamicin	0.0125	6	8	0.86	0.68	382
Trimethoprim/sulfamethoxazole	0.0250	4	7	0.82	0.89	305
Nitrofurantoin	0.0125	5	1	0.74	0.64	364
Vancomycin	0.0125	7	9	0.55	0.70	266
CDI	0.3000	6	9	0.84	0.53	743
Toxicity	0.3000	6	9	0.91	0.83	620

Supplementary Fig. 3: Highest feature importances in the clinical prediction models

The ten most important features for predictions made by each of the 15 clinical prediction models, measured using Shapley additive explanation (SHAP) values. Dots correspond to groups of cases in the training dataset, with dot size indicating the size of the group. Dot colour indicates the actual value of that feature in the traning dataset, on a gradient from red representing low continuous values or the absence of a feature, to green representing high continuous values or the presence of a feature.

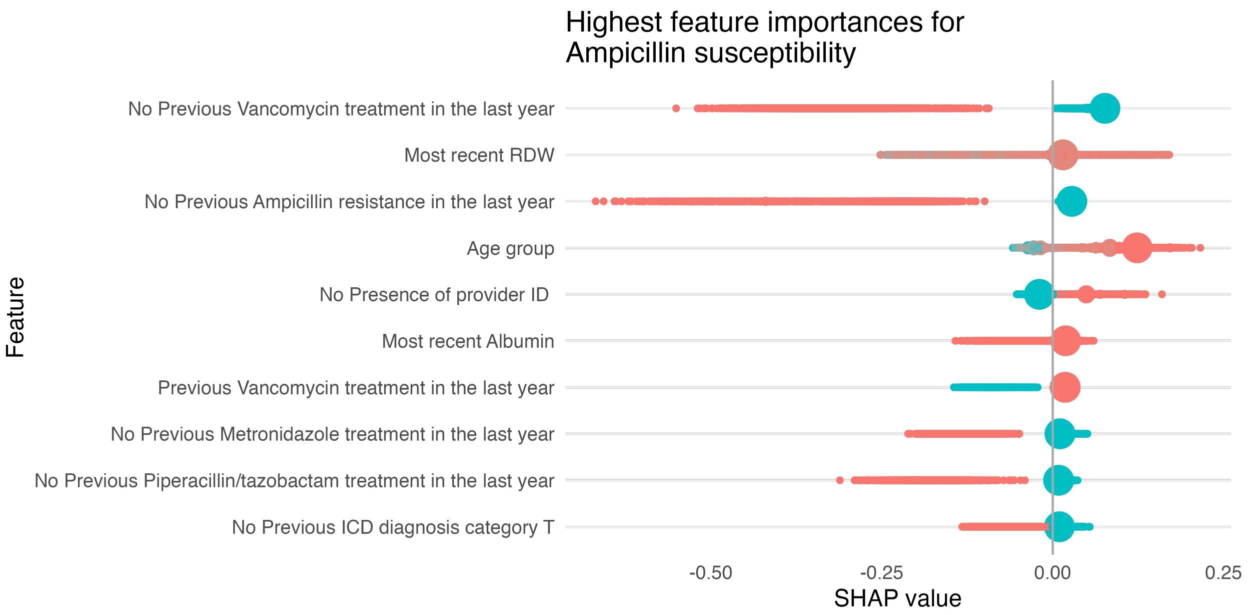
Highest feature importances for
Ampicillin susceptibility
No Previous Vancomycin treatment in the last year
Most recent RDW
No Previous Ampicillin resistance in the last year
Age group
No Presence of provider ID
Most recent Albumin
Previous Vancomycin treatment in the last year
No Previous Metronidazole treatment in the last year
No Previous Piperacillin/tazobactam treatment in the last year
No Previous ICD diagnosis category T
Feature
-0.50
-0.25
0.00
0.25
SHAP value

58

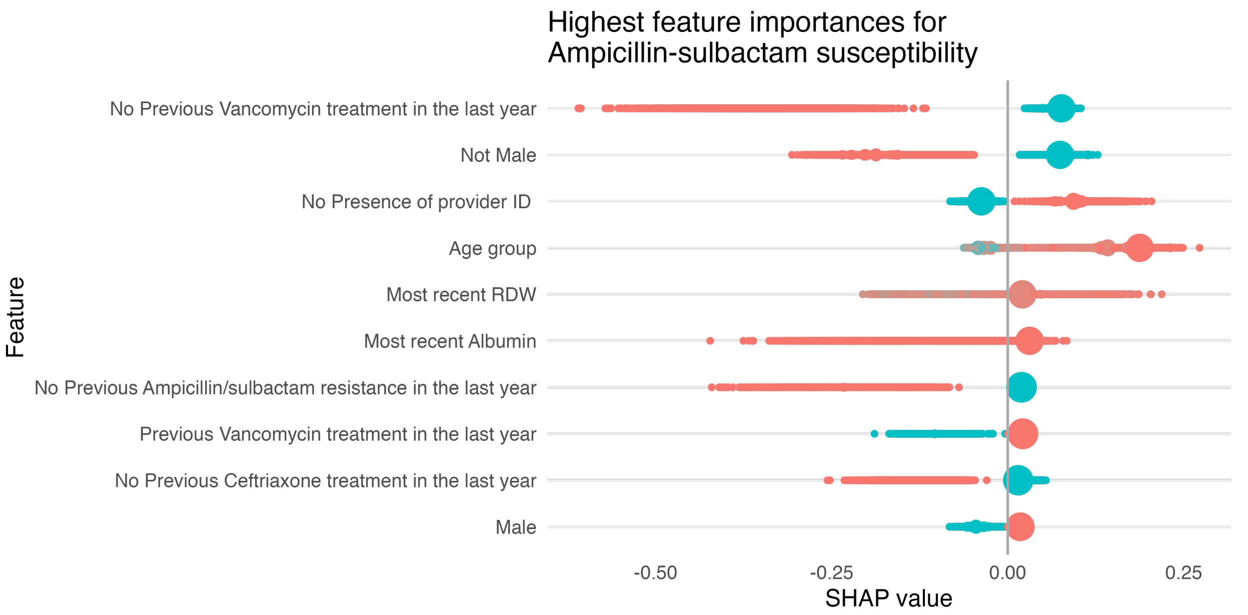
Highest feature importances for
Ampicillin-sulbactam susceptibility
No Previous Vancomycin treatment in the last year
Not Male
No Presence of provider ID
Age group
Most recent RDW
Most recent Albumin
No Previous Ampicillin/sulbactam resistance in the last year
Previous Vancomycin treatment in the last year
No Previous Ceftriaxone treatment in the last year
Male
Feature
-0.50
-0.25
0.00
0.25
SHAP value

59

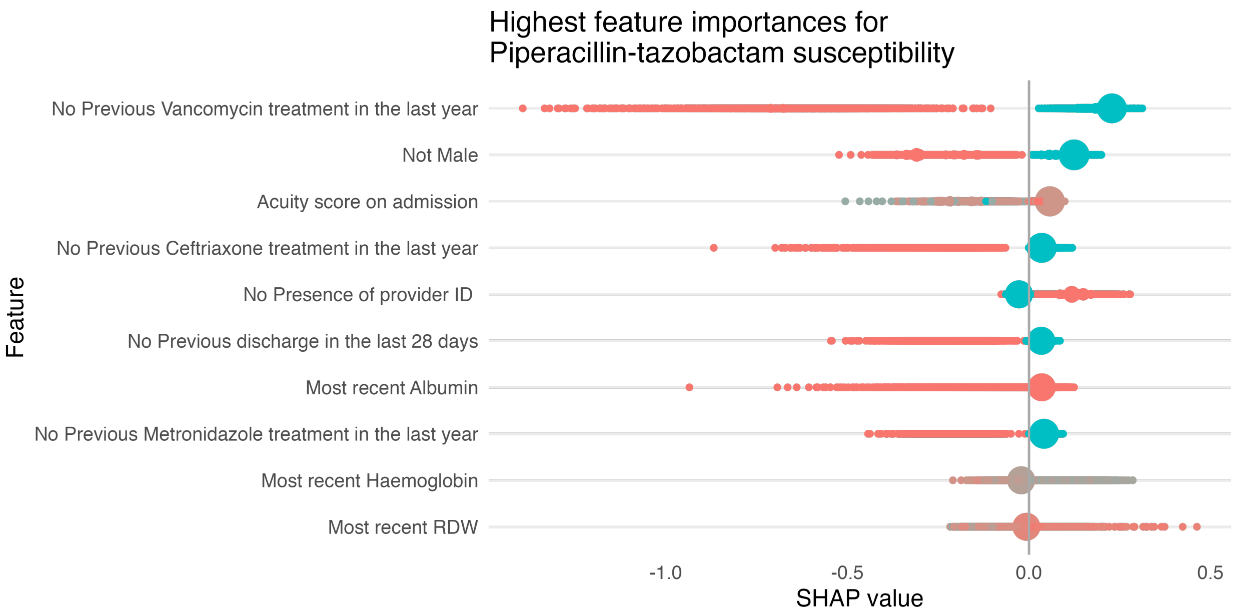

Highest feature importances for
Piperacillin-tazobactam susceptibility
No Previous Vancomycin treatment in the last year
Not Male
Acuity score on admission
No Previous Ceftriaxone treatment in the last year
No Presence of provider ID
No Previous discharge in the last 28 days
Most recent Albumin
No Previous Metronidazole treatment in the last year
Most recent Haemoglobin
Most recent RDW
Feature
-1.0
-0.5
0.0
0.5
SHAP value

60

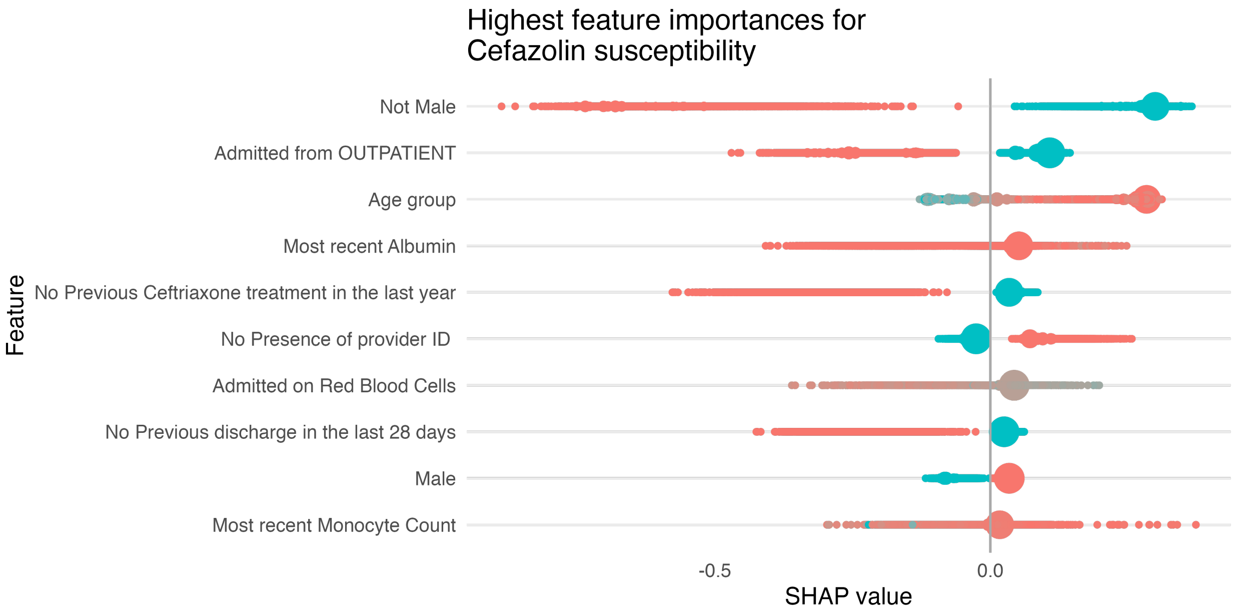

Highest feature importances for
Cefazolin susceptibility
Not Male
Admitted from OUTPATIENT
Age group
Most recent Albumin
No Previous Ceftriaxone treatment in the last year
No Presence of provider ID
Admitted on Red Blood Cells
No Previous discharge in the last 28 days
Male
Most recent Monocyte Count
Feature
-0.5
0.0
SHAP value

61

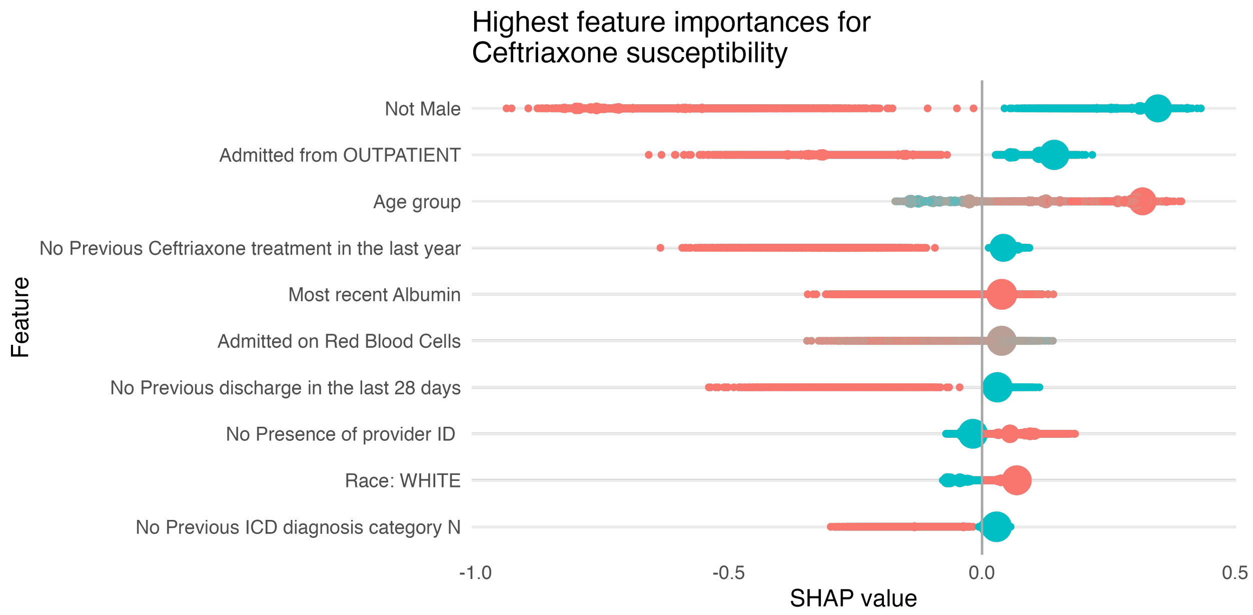
Highest feature importances for
Ceftriaxone susceptibility
Not Male
Admitted from OUTPATIENT
Age group
No Previous Ceftriaxone treatment in the last year
Most recent Albumin
Admitted on Red Blood Cells
No Previous discharge in the last 28 days
No Presence of provider ID
Race: WHITE
No Previous ICD diagnosis category N
Feature
-1.0
-0.5
0.0
0.5
SHAP value

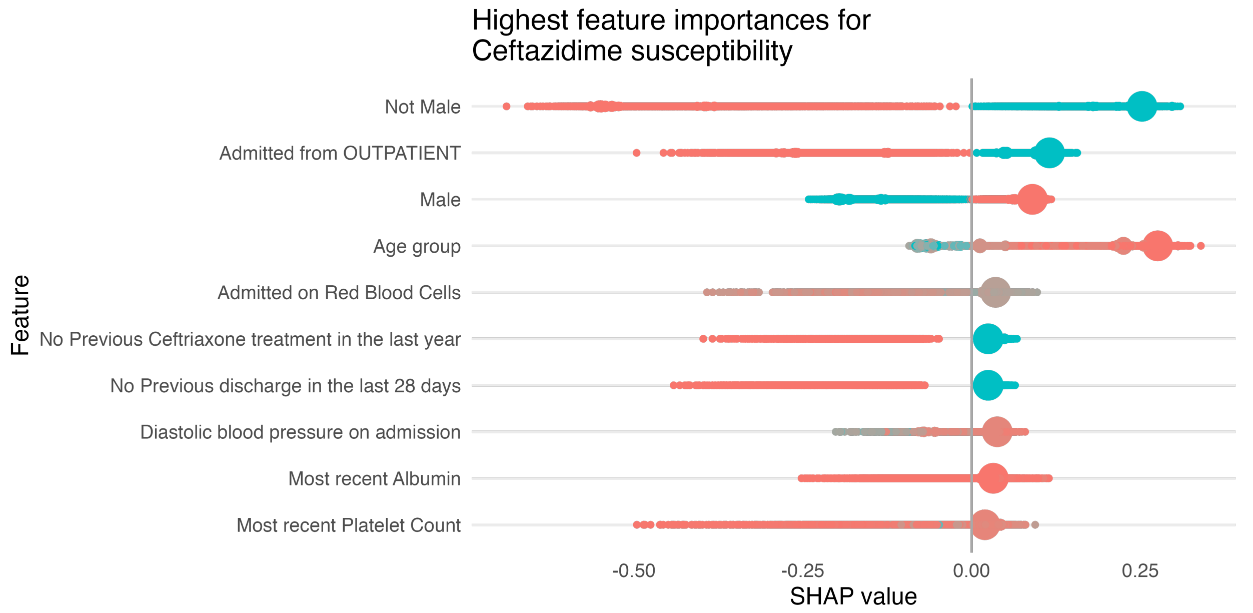
Highest feature importances for
Ceftazidime susceptibility
Not Male
Admitted from OUTPATIENT
Male
Age group
Admitted on Red Blood Cells
No Previous Ceftriaxone treatment in the last year
No Previous discharge in the last 28 days
Diastolic blood pressure on admission
Most recent Albumin
Most recent Platelet Count
Feature
-0.50
-0.25
0.00
0.25
SHAP value

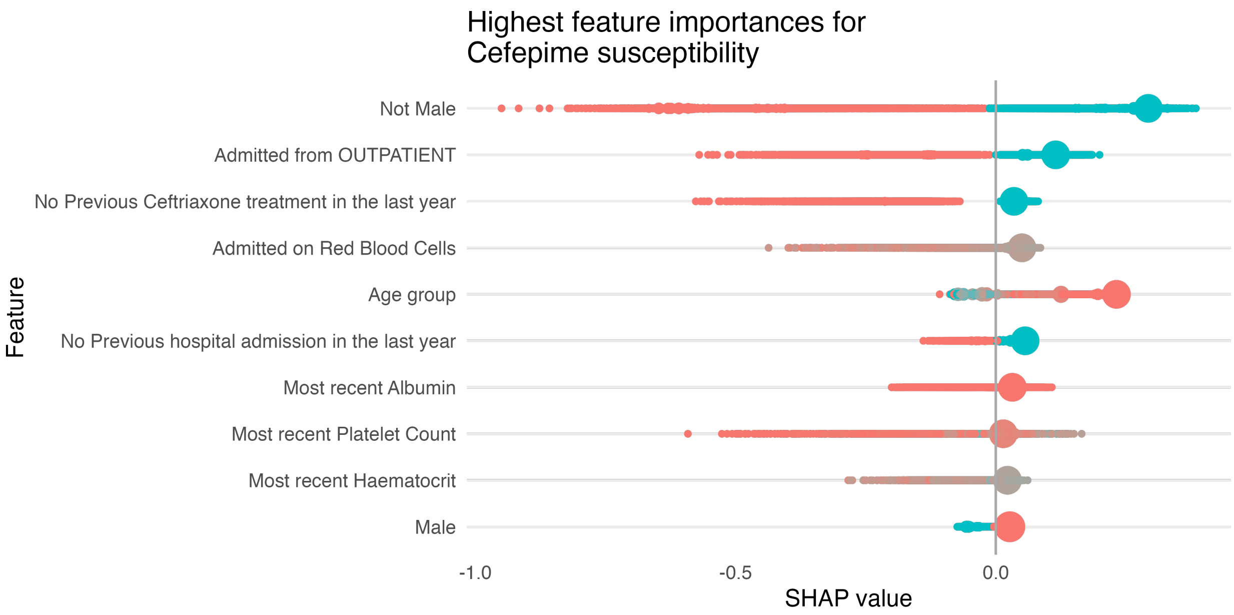
Highest feature importances for
Cefepime susceptibility
Not Male
Admitted from OUTPATIENT
No Previous Ceftriaxone treatment in the last year
Admitted on Red Blood Cells
Age group
No Previous hospital admission in the last year
Most recent Albumin
Most recent Platelet Count
Most recent Haematocrit
Male
Feature
-1.0
-0.5
0.0
SHAP value

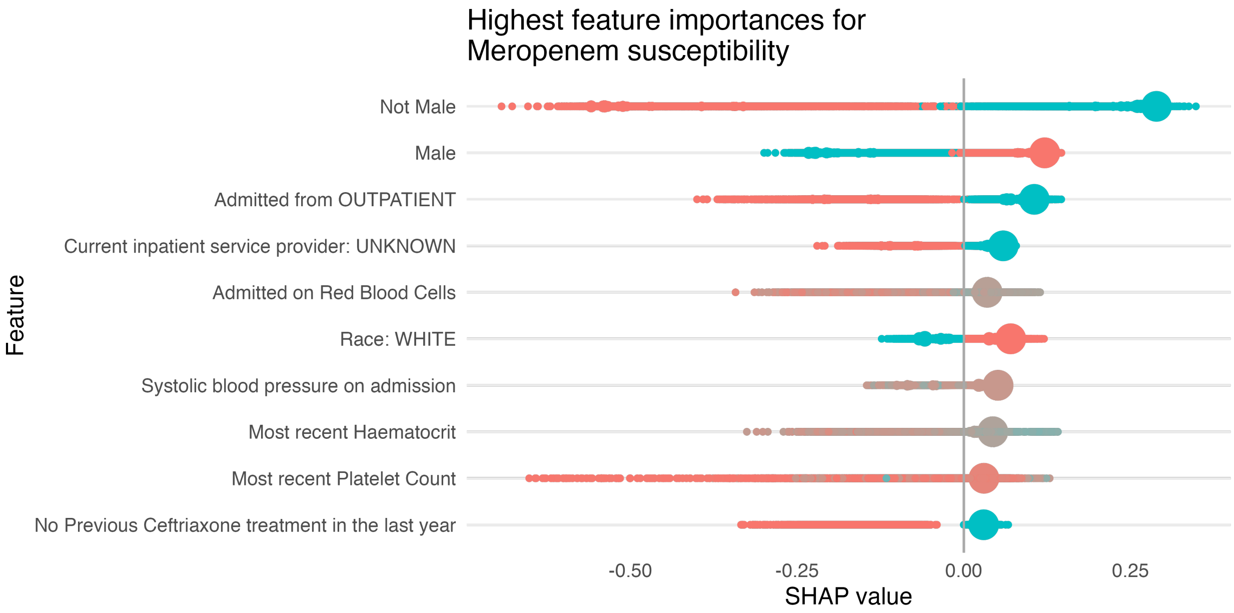
Highest feature importances for
Meropenem susceptibility
Not Male
Male
Admitted from OUTPATIENT
Current inpatient service provider: UNKNOWN
Admitted on Red Blood Cells
Race: WHITE
Systolic blood pressure on admission
Most recent Haematocrit
Most recent Platelet Count
No Previous Ceftriaxone treatment in the last year
Feature
-0.50
-0.25
0.00
0.25
SHAP value

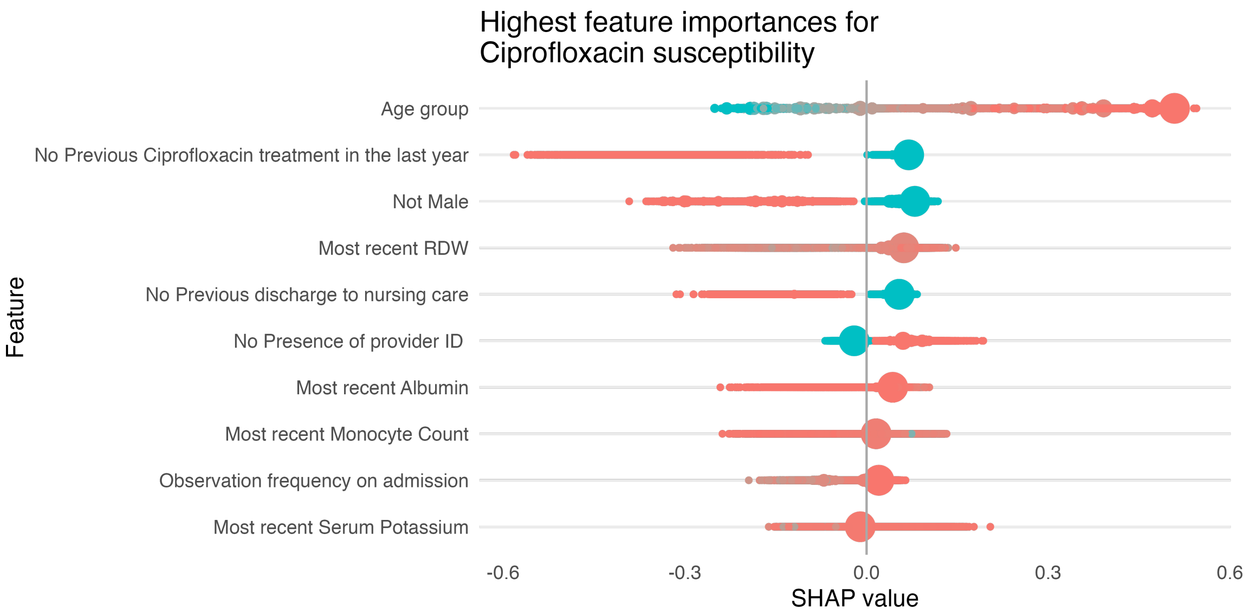
Highest feature importances for
Ciprofloxacin susceptibility
Age group
No Previous Ciprofloxacin treatment in the last year
Not Male
Most recent RDW
No Previous discharge to nursing care
No Presence of provider ID
Most recent Albumin
Most recent Monocyte Count
Observation frequency on admission
Most recent Serum Potassium
Feature
-0.6
-0.3
0.0
0.3
0.6
SHAP value

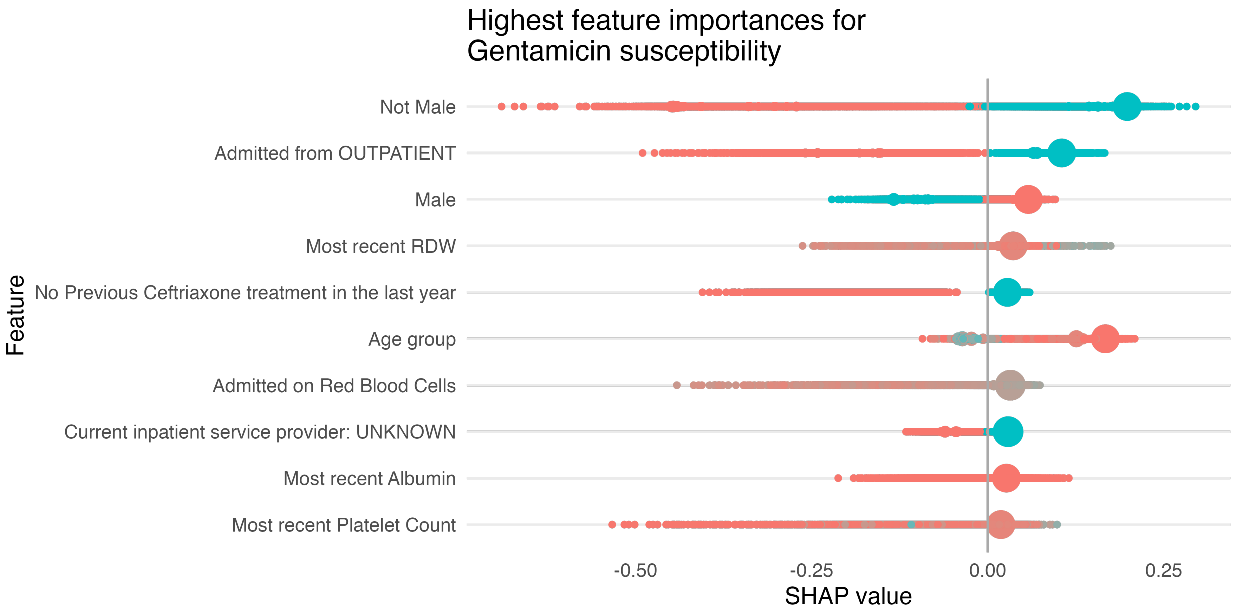
Highest feature importances for
Gentamicin susceptibility
Not Male
Admitted from OUTPATIENT
Male
Most recent RDW
No Previous Ceftriaxone treatment in the last year
Age group
Admitted on Red Blood Cells
Current inpatient service provider: UNKNOWN
Most recent Albumin
Most recent Platelet Count
Feature
-0.50
-0.25
0.00
0.25
SHAP value

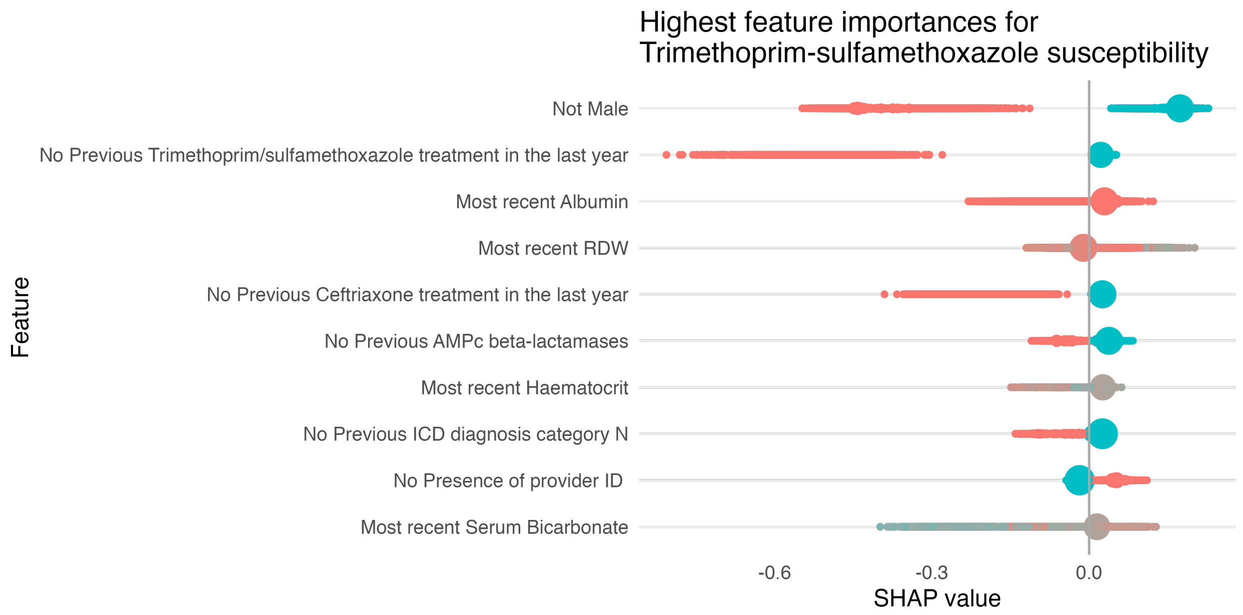

Highest feature importances for
Trimethoprim-sulfamethoxazole susceptibility
Not Male
No Previous Trimethoprim/sulfamethoxazole treatment in the last year
Most recent Albumin
Most recent RDW
No Previous Ceftriaxone treatment in the last year
No Previous AMPc beta-lactamases
Most recent Haematocrit
No Previous ICD diagnosis category N
No Presence of provider ID
Most recent Serum Bicarbonate
Feature
-0.6
-0.3
0.0
SHAP value

68

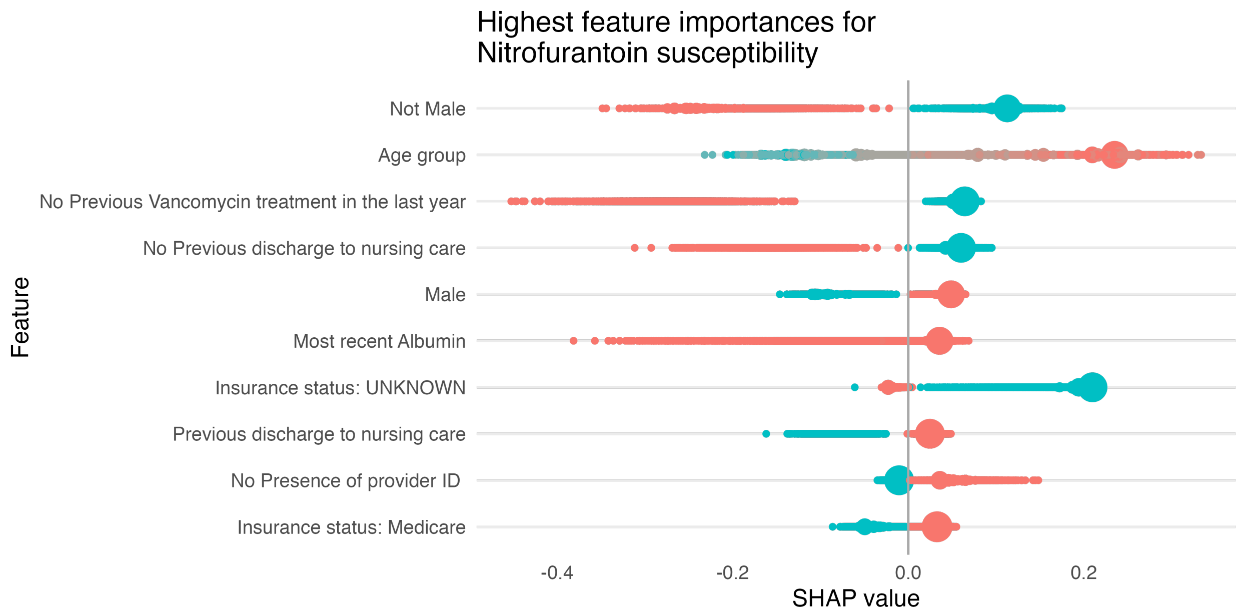

Highest feature importances for
Nitrofurantoin susceptibility
Not Male
Age group
No Previous Vancomycin treatment in the last year
No Previous discharge to nursing care
Male
Most recent Albumin
Insurance status: UNKNOWN
Previous discharge to nursing care
No Presence of provider ID
Insurance status: Medicare
Feature
-0.4
-0.2
0.0
0.2
SHAP value

69

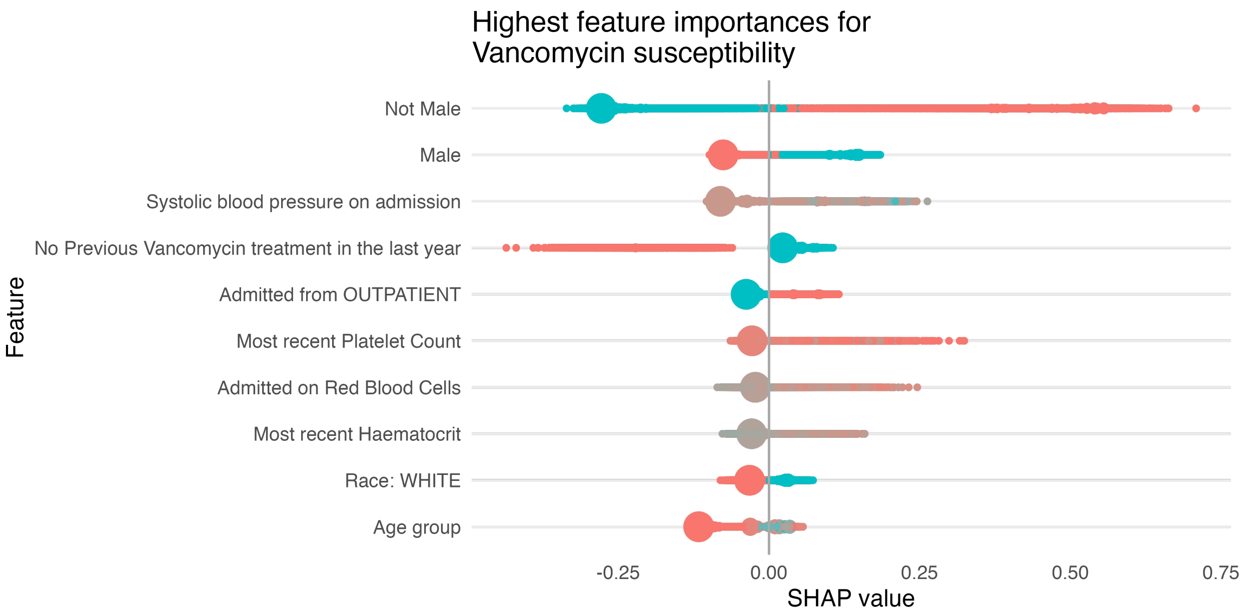
Highest feature importances for
Vancomycin susceptibility
Feature
Not Male
Male
Systolic blood pressure on admission
No Previous Vancomycin treatment in the last year
Admitted from OUTPATIENT
Most recent Platelet Count
Admitted on Red Blood Cells
Most recent Haematocrit
Race: WHITE
Age group
-0.25
0.00
0.25
0.50
0.75
SHAP value

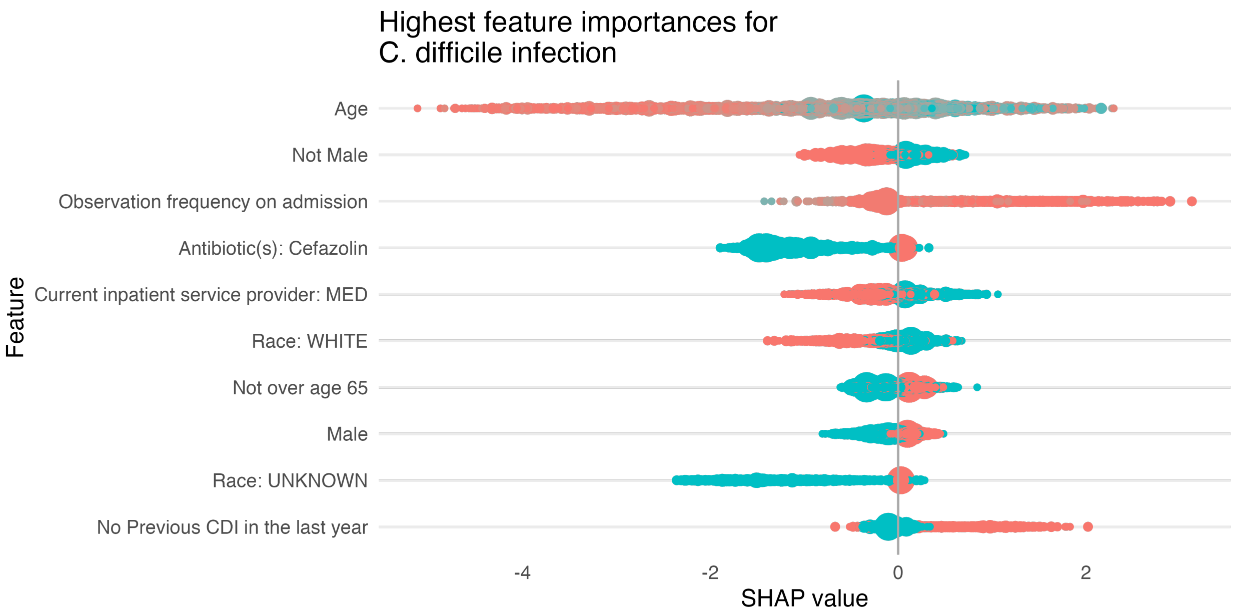
Highest feature importances for
C. difficile infection
Feature
Age
Not Male
Observation frequency on admission
Antibiotic(s): Cefazolin
Current inpatient service provider: MED
Race: WHITE
Not over age 65
Male
Race: UNKNOWN
No Previous CDI in the last year
-4
-2
0
2
SHAP value

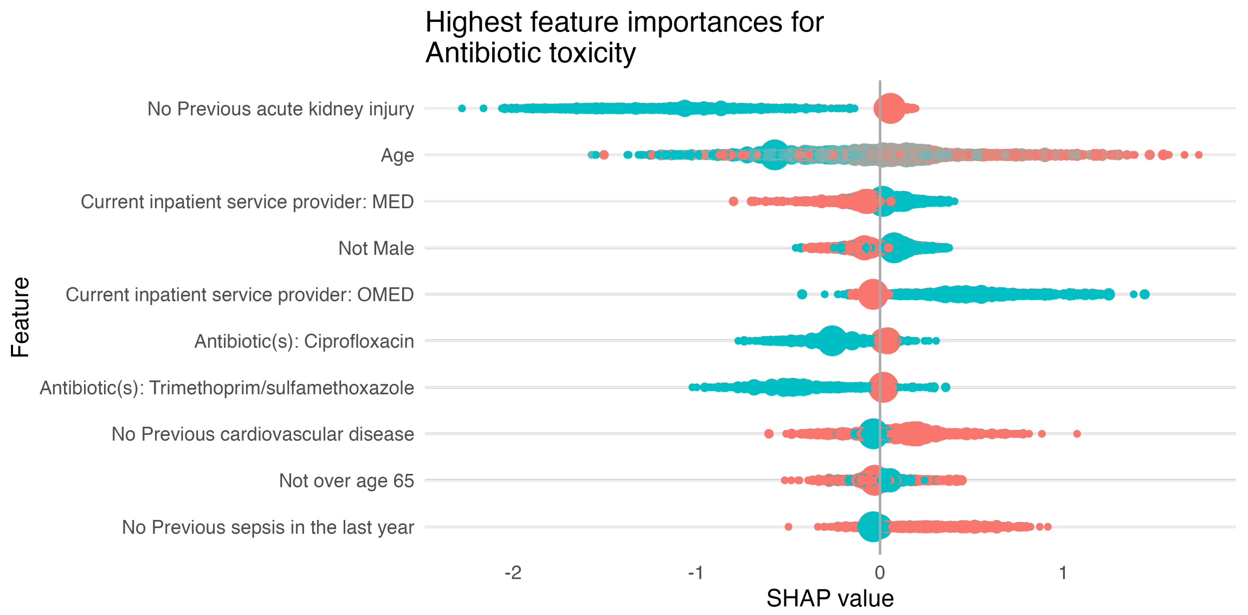

Supplementary Table 3: Model performance metrics

Model	AUROC	Precision	Recall	F1 score	Accuracy
Ampicillin	0.613 (0.597-0.63)	0.553 (0.533-0.571)	0.608 (0.587-0.628)	0.579 (0.562-0.596)	0.581 (0.566-0.595)
Ampicillin-sulbactam	0.663 (0.647-0.682)	0.761 (0.748-0.774)	0.957 (0.95-0.963)	0.848 (0.84-0.857)	0.751 (0.739-0.763)
Piperacillin-tazobactam	0.76 (0.733-0.79)	0.931 (0.924-0.939)	0.997 (0.995-0.998)	0.963 (0.959-0.967)	0.928 (0.921-0.936)
Cefazolin	0.742 (0.728-0.756)	0.716 (0.7-0.732)	0.801 (0.787-0.815)	0.756 (0.745-0.768)	0.696 (0.684-0.709)
Ceftriaxone	0.76 (0.746-0.774)	0.755 (0.742-0.77)	0.87 (0.858-0.882)	0.808 (0.799-0.819)	0.726 (0.714-0.74)
Ceftazidime	0.733 (0.718-0.749)	0.764 (0.752-0.778)	0.945 (0.937-0.953)	0.845 (0.837-0.854)	0.747 (0.735-0.76)
Cefepime	0.718 (0.702-0.736)	0.8 (0.788-0.812)	0.98 (0.975-0.984)	0.881 (0.873-0.888)	0.792 (0.78-0.803)
Meropenem	0.734 (0.717-0.753)	0.833 (0.822-0.844)	0.987 (0.983-0.991)	0.904 (0.897-0.91)	0.827 (0.816-0.838)
Ciprofloxacin	0.676 (0.658-0.695)	0.797 (0.786-0.809)	0.986 (0.983-0.99)	0.882 (0.875-0.889)	0.791 (0.779-0.802)
Gentamicin	0.688 (0.67-0.707)	0.78 (0.768-0.792)	0.979 (0.974-0.983)	0.868 (0.861-0.876)	0.772 (0.761-0.784)
Trimethoprim-sulfamethoxazole	0.671 (0.654-0.687)	0.666 (0.65-0.682)	0.865 (0.852-0.879)	0.752 (0.741-0.764)	0.657 (0.644-0.671)
Nitrofurantoin	0.691 (0.669-0.711)	0.824 (0.813-0.835)	0.995 (0.993-0.997)	0.902 (0.895-0.909)	0.823 (0.811-0.834)
Vancomycin	0.712 (0.691-0.733)	NA (NA)	0 (0-0)	NA (NA)	0.864 (0.854-0.874)
CDI	0.88 (0.869-0.891)	0.695 (0.627-0.759)	0.123 (0.105-0.143)	0.209 (0.18-0.239)	0.987 (0.986-0.988)

Model	AUROC	Precision	Recall	F1 score	Accuracy
Toxicity	0.683 (0.678-0.687)	0.646 (0.627-0.668)	0.08 (0.075-0.084)	0.142 (0.135-0.148)	0.794 (0.791-0.797)

Performance metrics for prediction of single-antibiotic susceptibility, CDI (*Clostridioides difficile* infection), and antibiotic toxicity in the single validation run on the validation dataset (decision threshold 0.5 where applicable). 95% confidence intervals are displayed in parentheses.

Supplementary Fig. 4: Small dataset stability metrics

Small dataset stability metrics for single antibiotic, CDI, and toxicity models in six random train-test splits stratified by outcome at a sequence of small dataset sizes across 50 training runs of XGBoost.

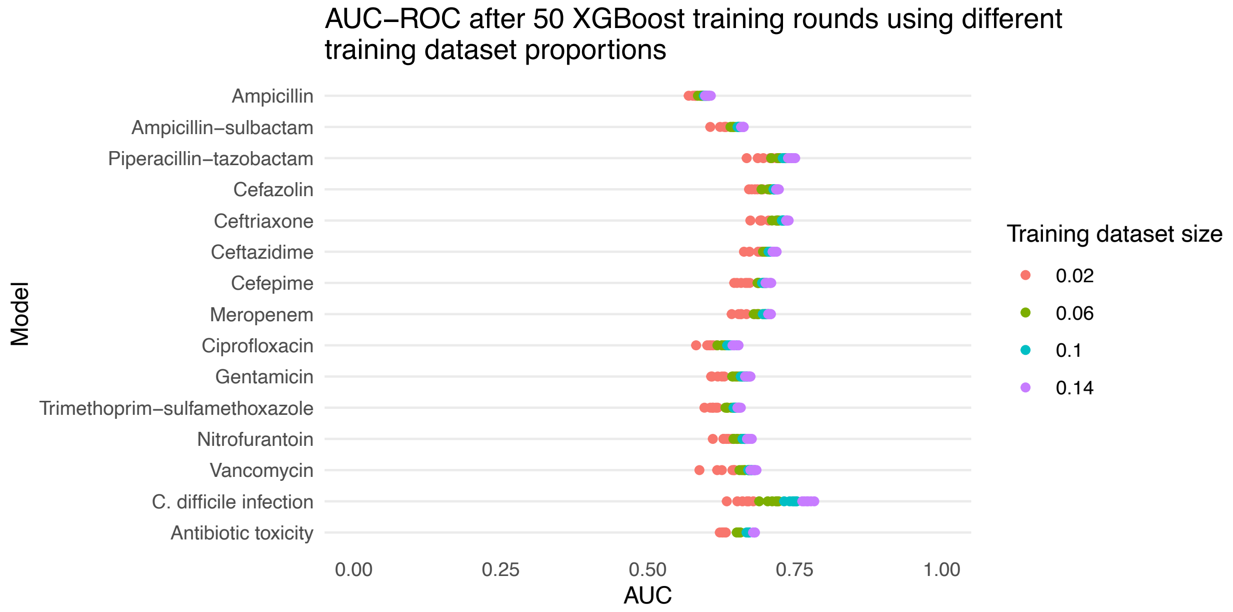

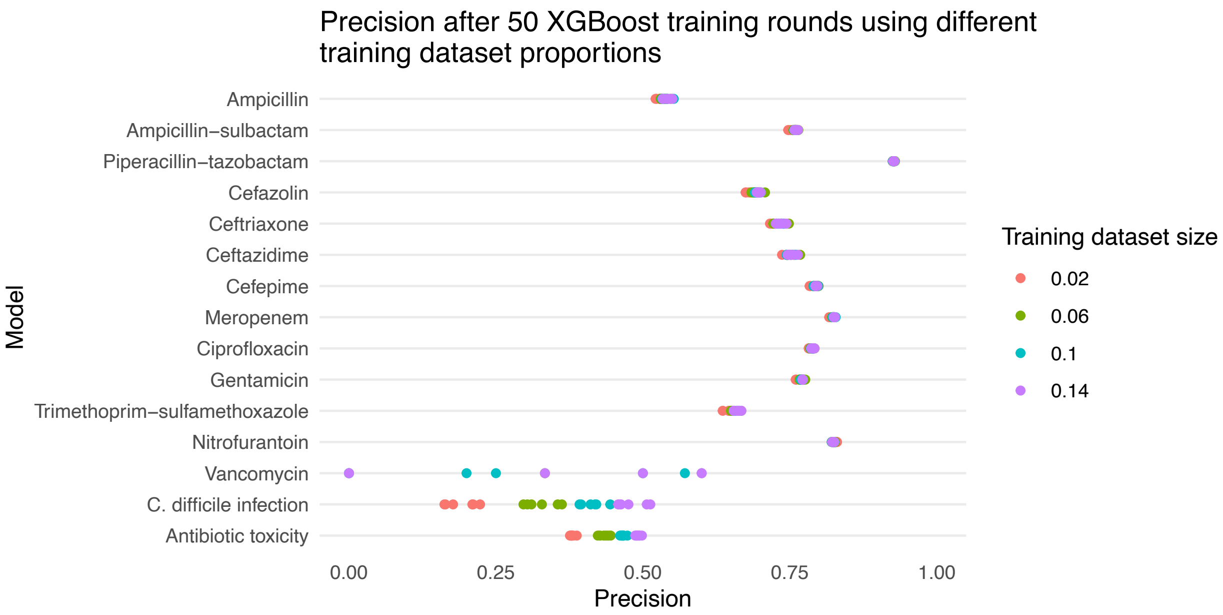
Precision after 50 XGBoost training rounds using different training dataset proportions
Ampicillin
Ampicillin−sulbactam
Piperacillin−tazobactam
Cefazolin
Ceftriaxone
Ceftazidime
Cefepime
Meropenem
Ciprofloxacin
Gentamicin
Trimethoprim−sulfamethoxazole
Nitrofurantoin
Vancomycin
C. difficile infection
Antibiotic toxicity
Model
0.00
0.25
0.50
0.75
1.00
Precision
Training dataset size
0.02
0.06
0.1
0.14

86

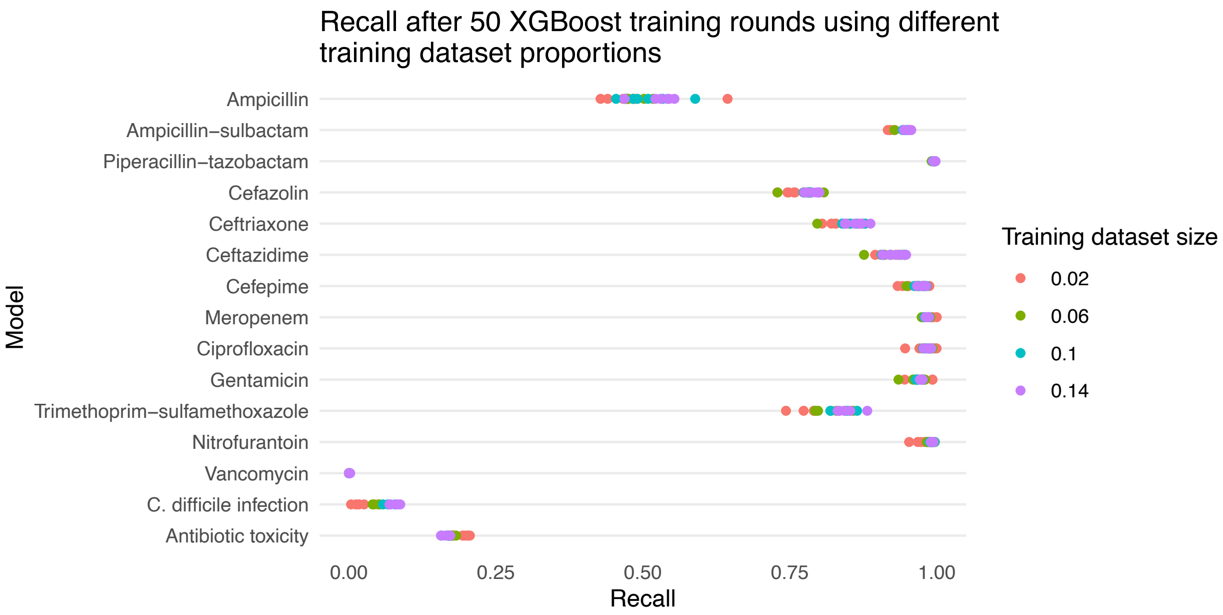
Recall after 50 XGBoost training rounds using different training dataset proportions
Ampicillin
Ampicillin−sulbactam
Piperacillin−tazobactam
Cefazolin
Ceftriaxone
Ceftazidime
Cefepime
Meropenem
Ciprofloxacin
Gentamicin
Trimethoprim−sulfamethoxazole
Nitrofurantoin
Vancomycin
C. difficile infection
Antibiotic toxicity
Model
0.00
0.25
0.50
0.75
1.00
Recall
Training dataset size
0.02
0.06
0.1
0.14

87

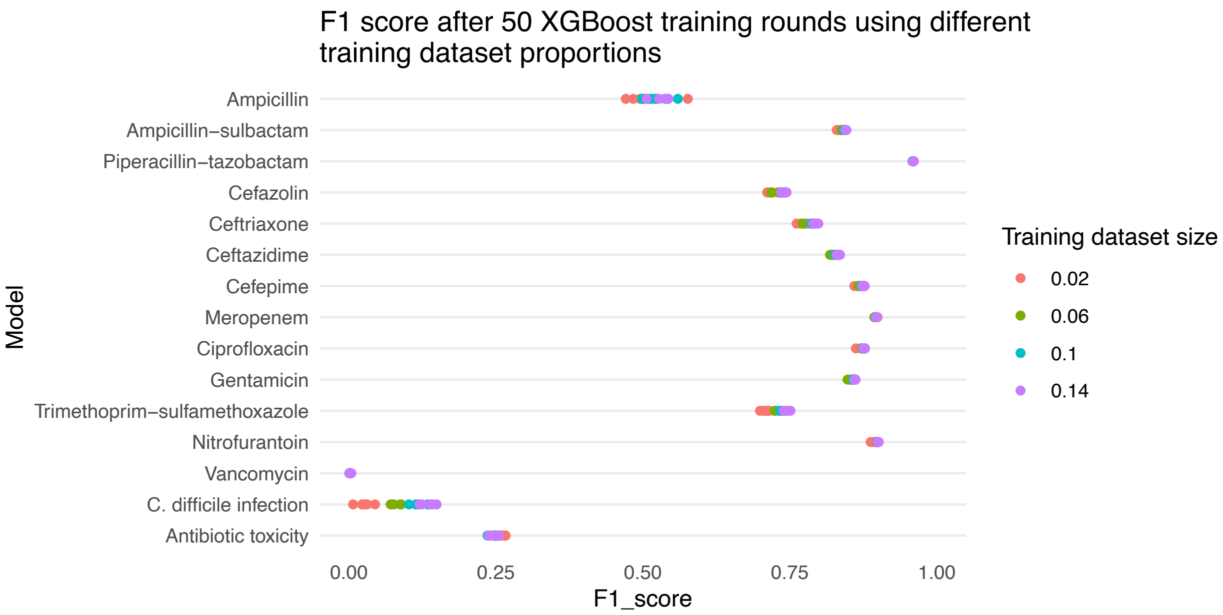

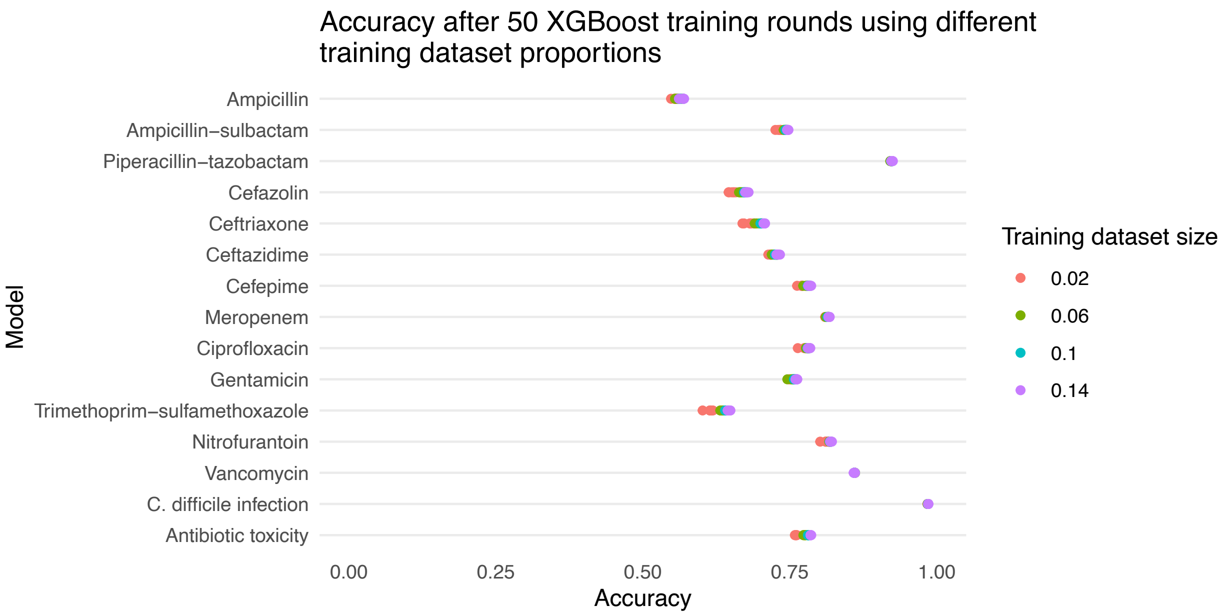

Supplementary Fig. 5: Year group cluster analysis

Year group cluster analysis of clinical prediction model performance in six random train-test splits stratified by outcome.

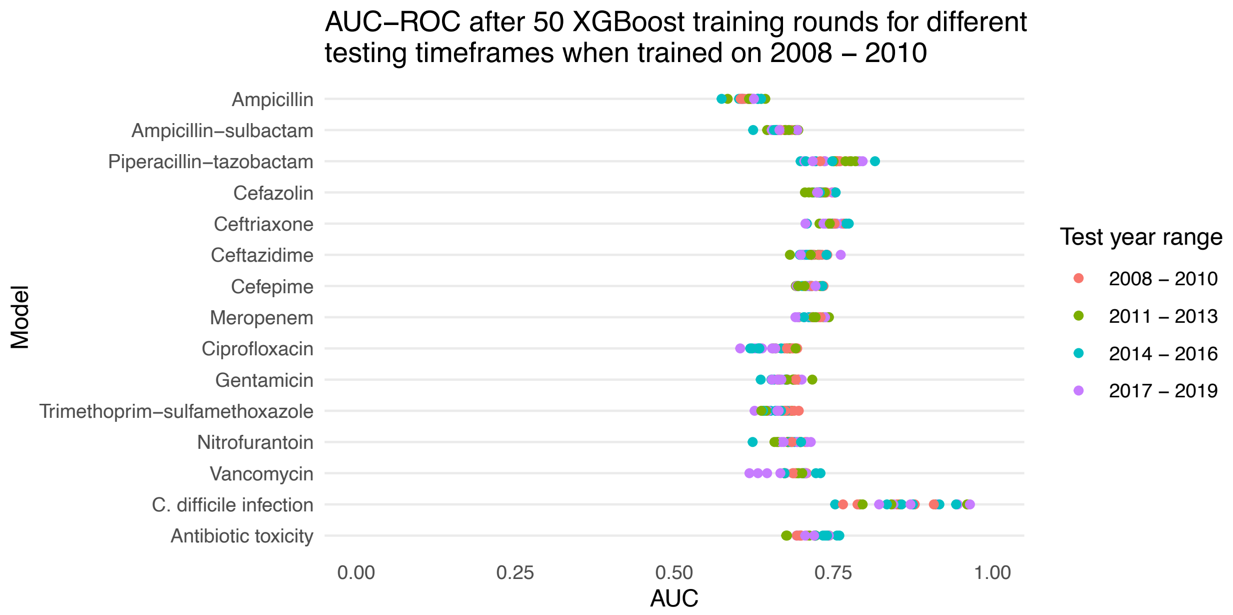
AUC–ROC after 50 XGBoost training rounds for different
testing timeframes when trained on 2008 – 2010
Model
Ampicillin
Ampicillin–sulbactam
Piperacillin–tazobactam
Cefazolin
Ceftriaxone
Ceftazidime
Cefepime
Meropenem
Ciprofloxacin
Gentamicin
Trimethoprim–sulfamethoxazole
Nitrofurantoin
Vancomycin
C. difficile infection
Antibiotic toxicity
0.00
0.25
0.50
0.75
1.00
AUC
Test year range
2008 – 2010
2011 – 2013
2014 – 2016
2017 – 2019

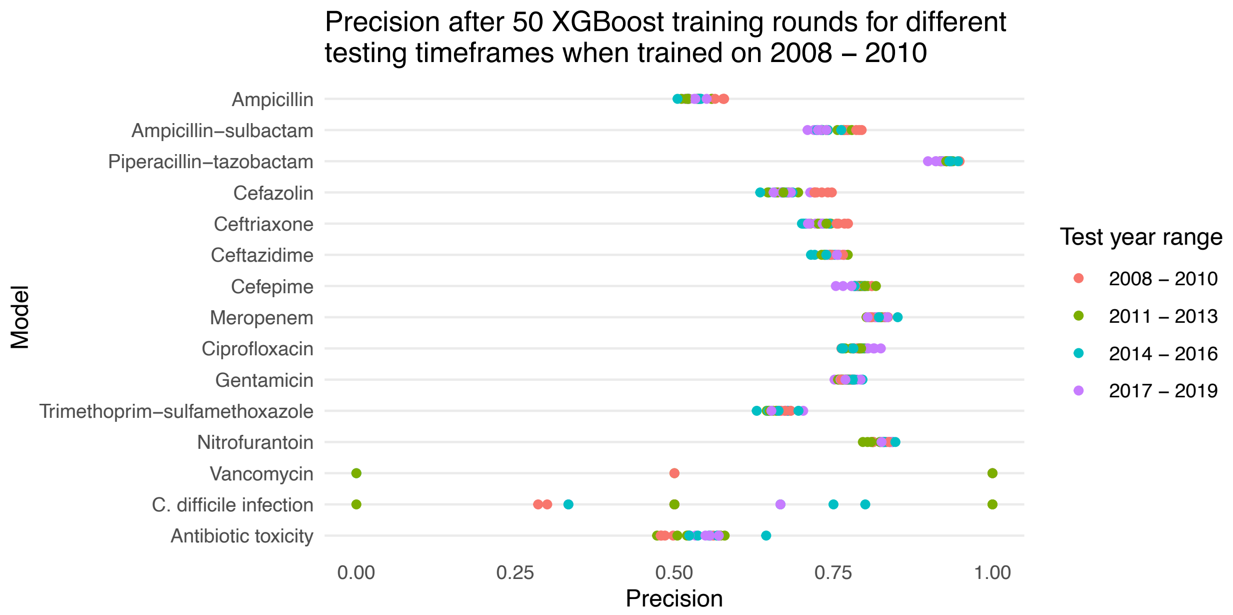
Precision after 50 XGBoost training rounds for different
testing timeframes when trained on 2008 – 2010
Model
Ampicillin
Ampicillin–sulbactam
Piperacillin–tazobactam
Cefazolin
Ceftriaxone
Ceftazidime
Cefepime
Meropenem
Ciprofloxacin
Gentamicin
Trimethoprim–sulfamethoxazole
Nitrofurantoin
Vancomycin
C. difficile infection
Antibiotic toxicity
0.00
0.25
0.50
0.75
1.00
Precision
Test year range
2008 – 2010
2011 – 2013
2014 – 2016
2017 – 2019

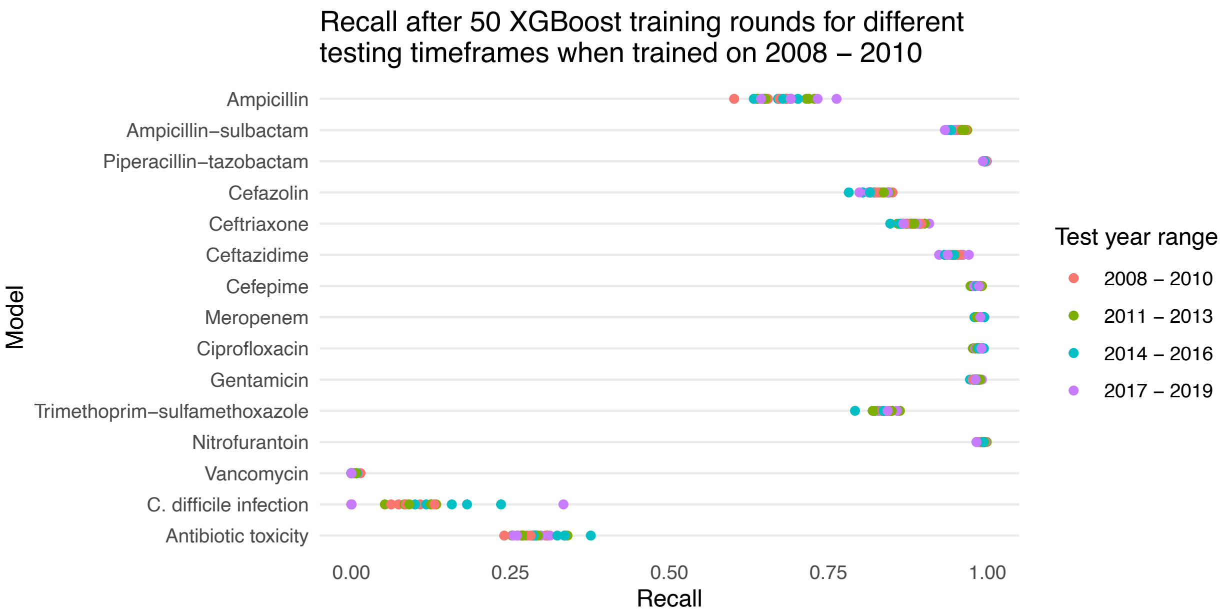
Recall after 50 XGBoost training rounds for different testing timeframes when trained on 2008 – 2010
Ampicillin
Ampicillin–sulbactam
Piperacillin–tazobactam
Cefazolin
Ceftriaxone
Ceftazidime
Cefepime
Meropenem
Ciprofloxacin
Gentamicin
Trimethoprim–sulfamethoxazole
Nitrofurantoin
Vancomycin
C. difficile infection
Antibiotic toxicity
Model
0.00
0.25
0.50
0.75
1.00
Recall
Test year range
2008 – 2010
2011 – 2013
2014 – 2016
2017 – 2019

97

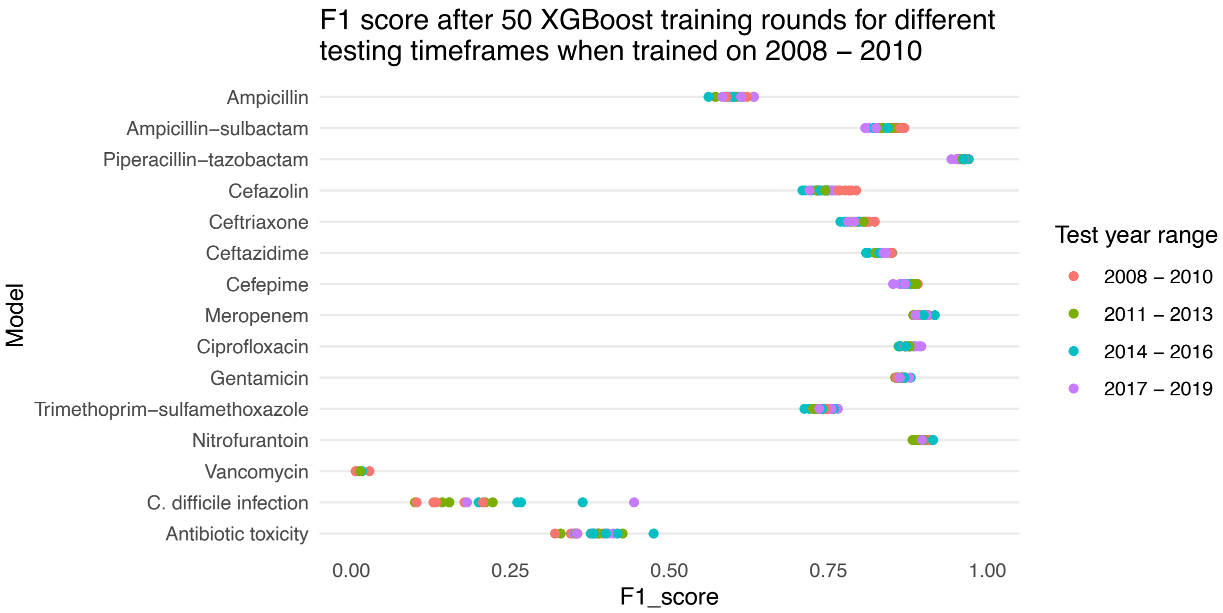
F1 score after 50 XGBoost training rounds for different testing timeframes when trained on 2008 – 2010
Ampicillin
Ampicillin–sulbactam
Piperacillin–tazobactam
Cefazolin
Ceftriaxone
Ceftazidime
Cefepime
Meropenem
Ciprofloxacin
Gentamicin
Trimethoprim–sulfamethoxazole
Nitrofurantoin
Vancomycin
C. difficile infection
Antibiotic toxicity
Model
0.00
0.25
0.50
0.75
1.00
F1_score
Test year range
2008 – 2010
2011 – 2013
2014 – 2016
2017 – 2019

98

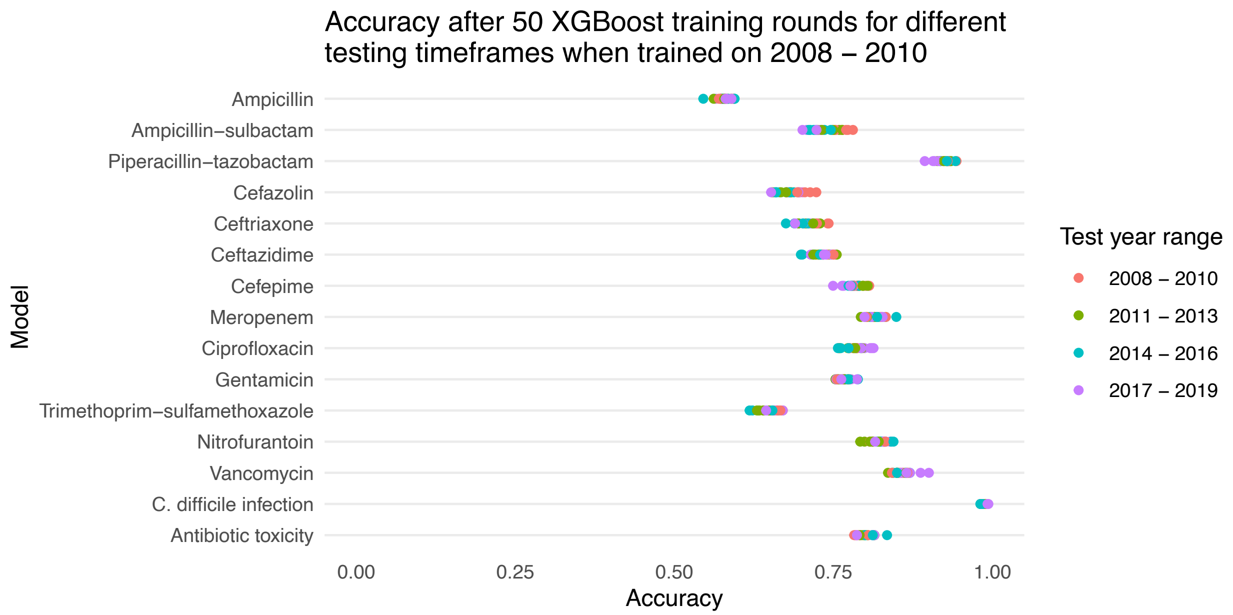

Accuracy after 50 XGBoost training rounds for different testing timeframes when trained on 2008 – 2010
Model
Ampicillin
Ampicillin–sulbactam
Piperacillin–tazobactam
Cefazolin
Ceftriaxone
Ceftazidime
Cefepime
Meropenem
Ciprofloxacin
Gentamicin
Trimethoprim–sulfamethoxazole
Nitrofurantoin
Vancomycin
C. difficile infection
Antibiotic toxicity
0.00
0.25
0.50
0.75
1.00
Accuracy
Test year range
2008 – 2010
2011 – 2013
2014 – 2016
2017 – 2019

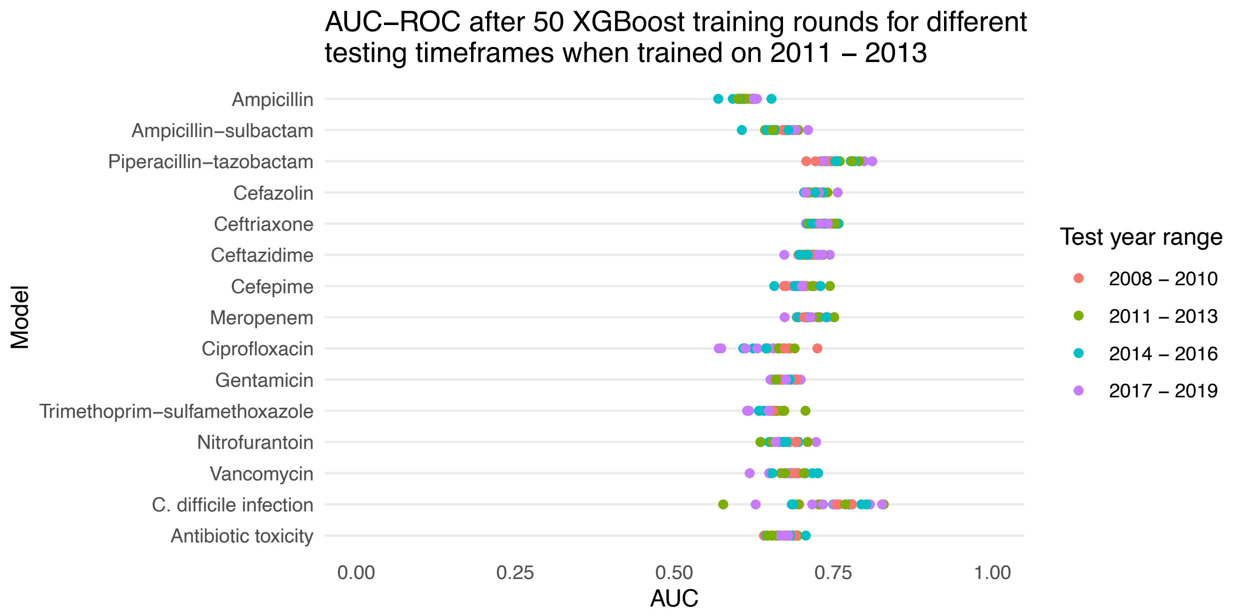

AUC–ROC after 50 XGBoost training rounds for different testing timeframes when trained on 2011 – 2013
Model
Ampicillin
Ampicillin–sulbactam
Piperacillin–tazobactam
Cefazolin
Ceftriaxone
Ceftazidime
Cefepime
Meropenem
Ciprofloxacin
Gentamicin
Trimethoprim–sulfamethoxazole
Nitrofurantoin
Vancomycin
C. difficile infection
Antibiotic toxicity
0.00
0.25
0.50
0.75
1.00
AUC
Test year range
2008 – 2010
2011 – 2013
2014 – 2016
2017 – 2019

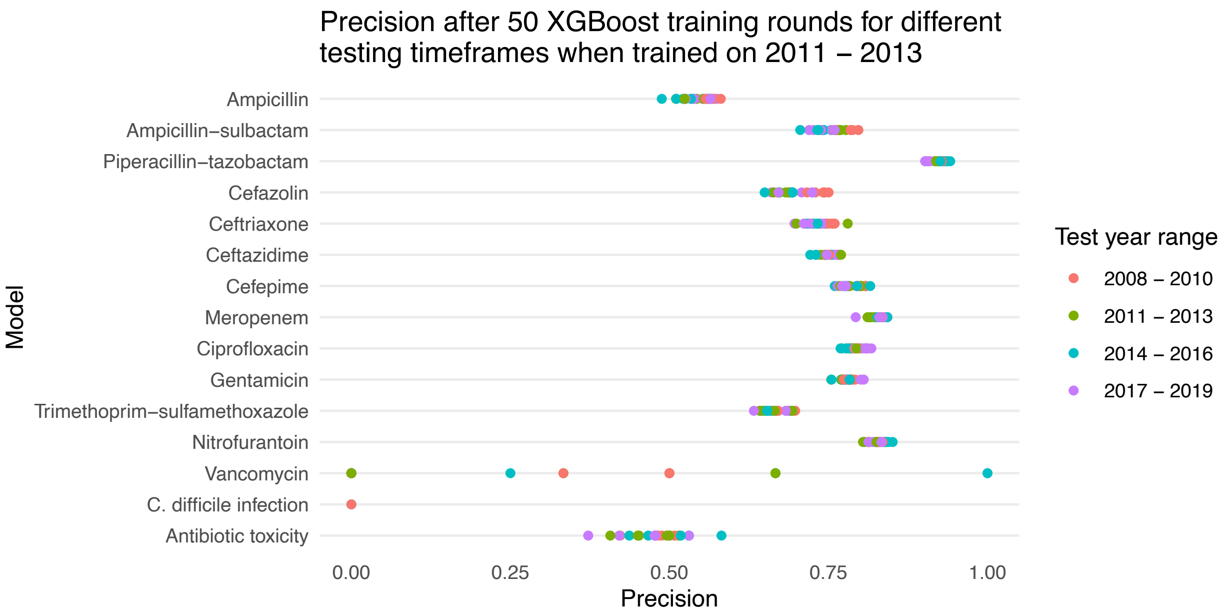

Precision after 50 XGBoost training rounds for different testing timeframes when trained on 2011 – 2013
Model
Ampicillin
Ampicillin−sulbactam
Piperacillin−tazobactam
Cefazolin
Ceftriaxone
Ceftazidime
Cefepime
Meropenem
Ciprofloxacin
Gentamicin
Trimethoprim−sulfamethoxazole
Nitrofurantoin
Vancomycin
C. difficile infection
Antibiotic toxicity
0.00
0.25
0.50
0.75
1.00
Precision
Test year range
2008 – 2010
2011 – 2013
2014 – 2016
2017 – 2019

101

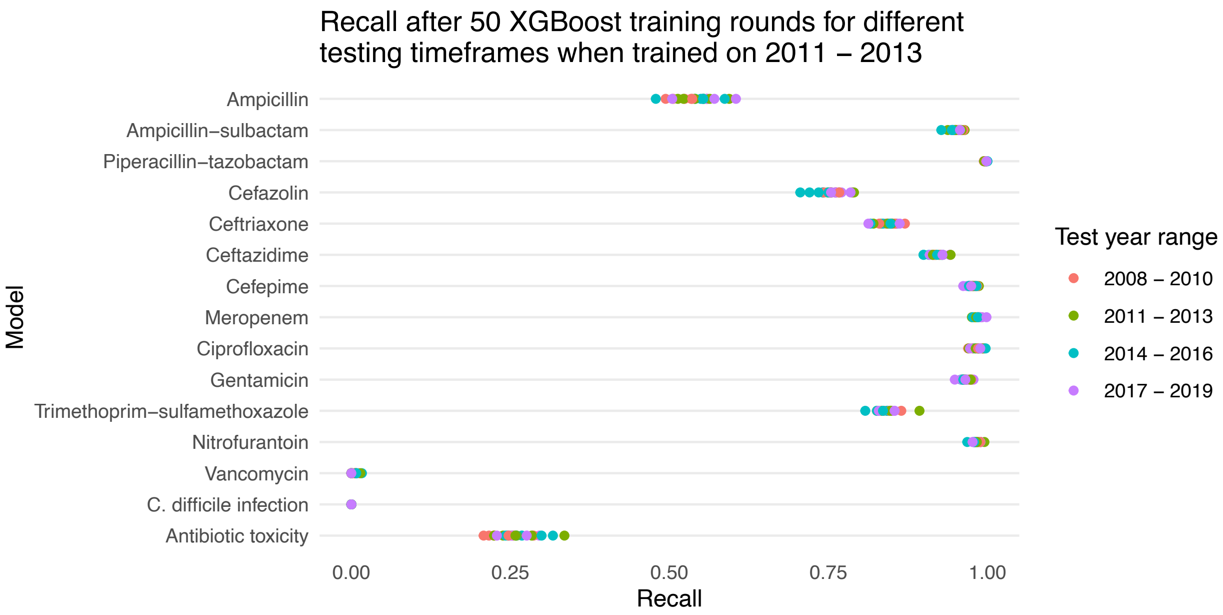

Recall after 50 XGBoost training rounds for different testing timeframes when trained on 2011 – 2013
Model
Ampicillin
Ampicillin−sulbactam
Piperacillin−tazobactam
Cefazolin
Ceftriaxone
Ceftazidime
Cefepime
Meropenem
Ciprofloxacin
Gentamicin
Trimethoprim−sulfamethoxazole
Nitrofurantoin
Vancomycin
C. difficile infection
Antibiotic toxicity
0.00
0.25
0.50
0.75
1.00
Recall
Test year range
2008 – 2010
2011 – 2013
2014 – 2016
2017 – 2019

102

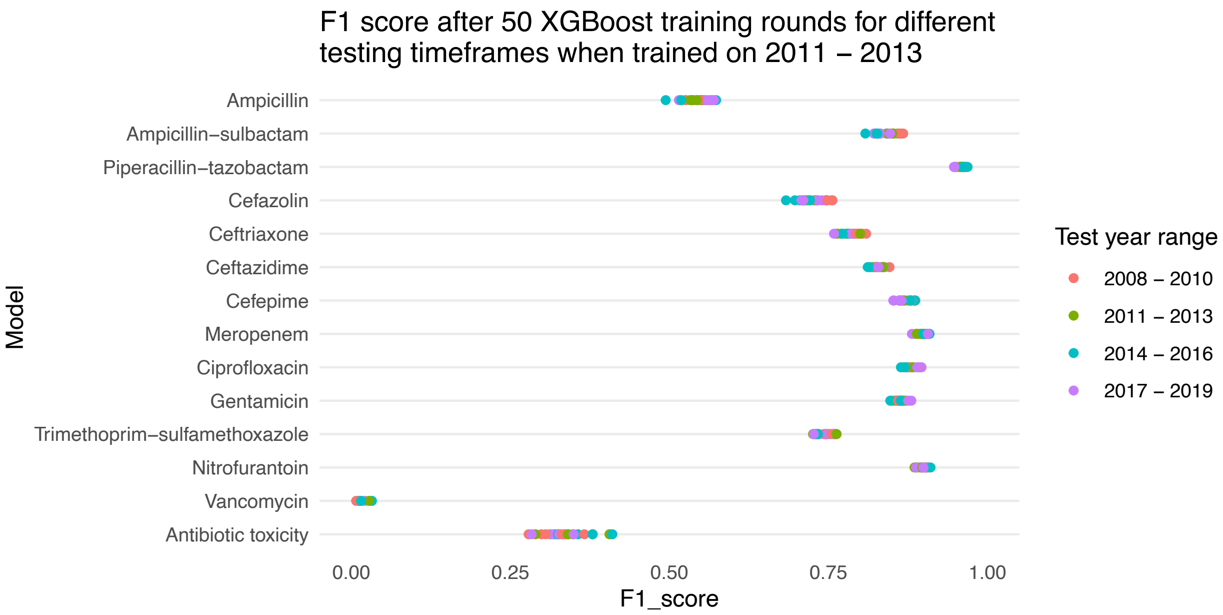
F1 score after 50 XGBoost training rounds for different testing timeframes when trained on 2011 – 2013
Model
Ampicillin
Ampicillin–sulbactam
Piperacillin–tazobactam
Cefazolin
Ceftriaxone
Ceftazidime
Cefepime
Meropenem
Ciprofloxacin
Gentamicin
Trimethoprim–sulfamethoxazole
Nitrofurantoin
Vancomycin
Antibiotic toxicity
0.00
0.25
0.50
0.75
1.00
F1_score
Test year range
2008 – 2010
2011 – 2013
2014 – 2016
2017 – 2019

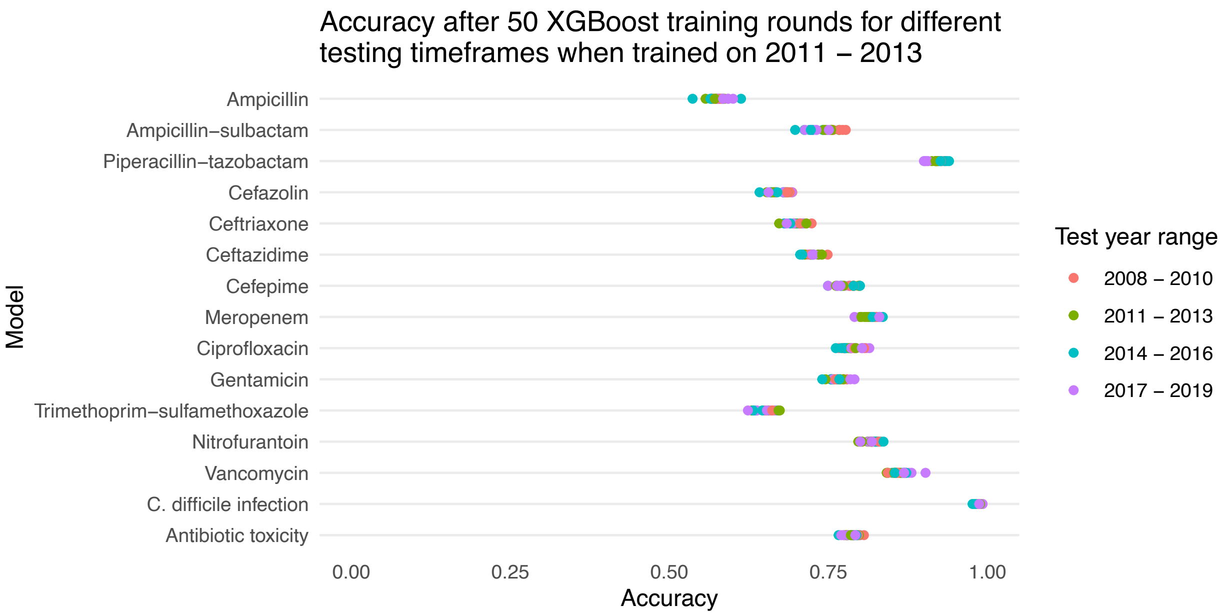
Accuracy after 50 XGBoost training rounds for different testing timeframes when trained on 2011 – 2013
Model
Ampicillin
Ampicillin–sulbactam
Piperacillin–tazobactam
Cefazolin
Ceftriaxone
Ceftazidime
Cefepime
Meropenem
Ciprofloxacin
Gentamicin
Trimethoprim–sulfamethoxazole
Nitrofurantoin
Vancomycin
C. difficile infection
Antibiotic toxicity
0.00
0.25
0.50
0.75
1.00
Accuracy
Test year range
2008 – 2010
2011 – 2013
2014 – 2016
2017 – 2019

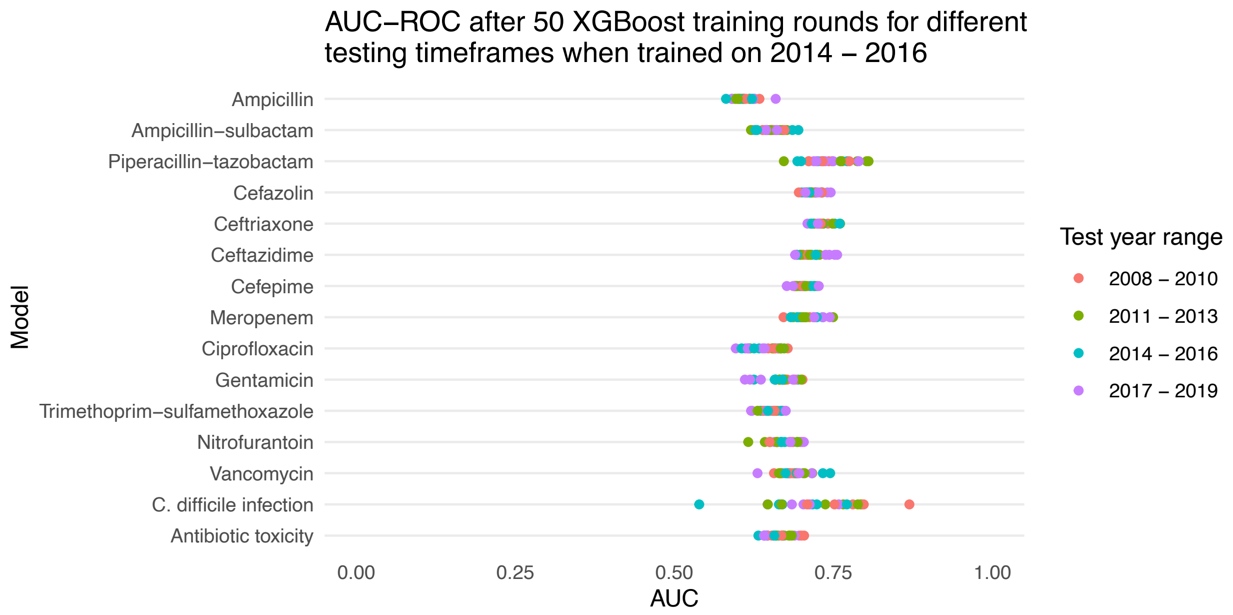
AUC−ROC after 50 XGBoost training rounds for different
testing timeframes when trained on 2014 – 2016
Model
Ampicillin
Ampicillin−sulbactam
Piperacillin−tazobactam
Cefazolin
Ceftriaxone
Ceftazidime
Cefepime
Meropenem
Ciprofloxacin
Gentamicin
Trimethoprim−sulfamethoxazole
Nitrofurantoin
Vancomycin
C. difficile infection
Antibiotic toxicity
0.00
0.25
0.50
0.75
1.00
AUC
Test year range
2008 – 2010
2011 – 2013
2014 – 2016
2017 – 2019

105

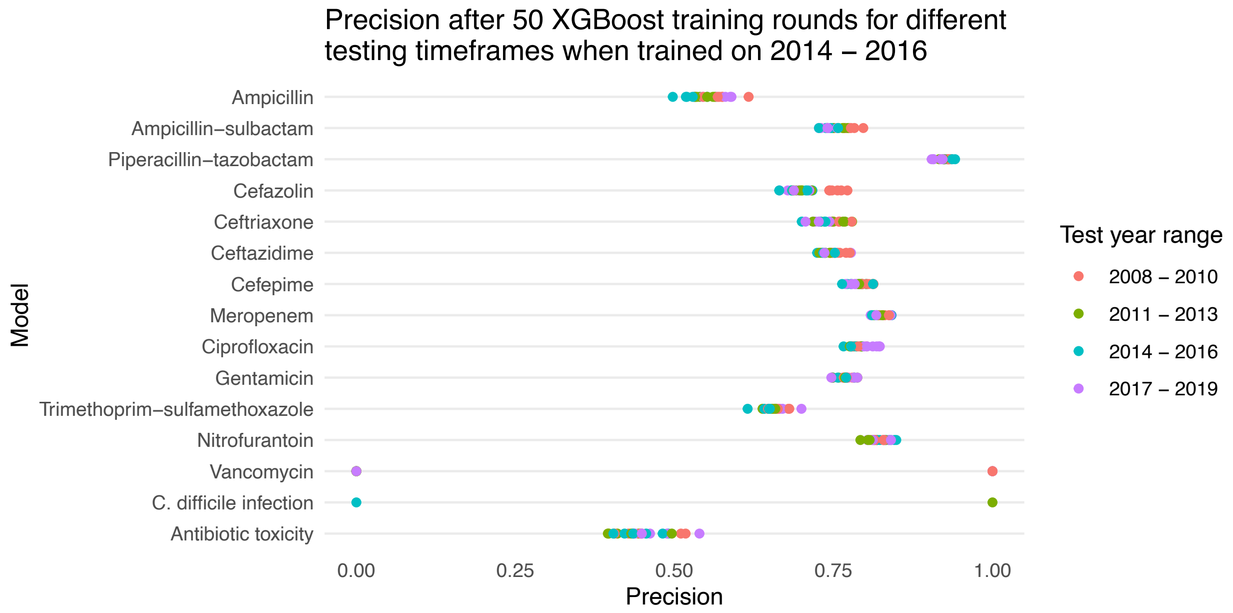
Precision after 50 XGBoost training rounds for different
testing timeframes when trained on 2014 – 2016
Model
Ampicillin
Ampicillin−sulbactam
Piperacillin−tazobactam
Cefazolin
Ceftriaxone
Ceftazidime
Cefepime
Meropenem
Ciprofloxacin
Gentamicin
Trimethoprim−sulfamethoxazole
Nitrofurantoin
Vancomycin
C. difficile infection
Antibiotic toxicity
0.00
0.25
0.50
0.75
1.00
Precision
Test year range
2008 – 2010
2011 – 2013
2014 – 2016
2017 – 2019

106

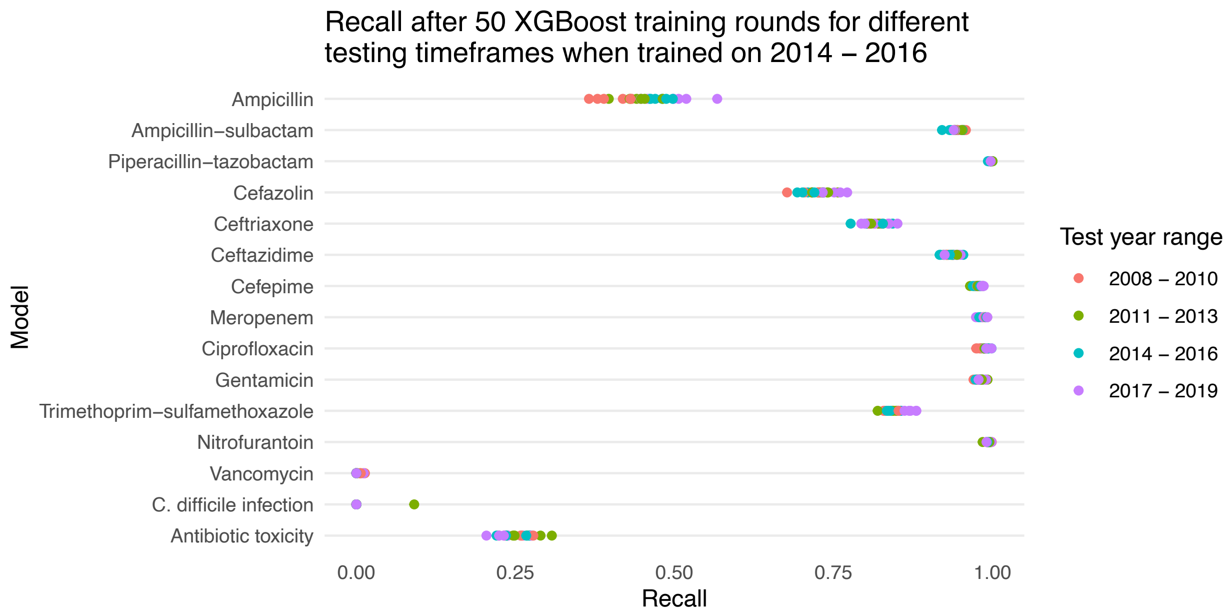
Recall after 50 XGBoost training rounds for different testing timeframes when trained on 2014 – 2016
Ampicillin
Ampicillin–sulbactam
Piperacillin–tazobactam
Cefazolin
Ceftriaxone
Ceftazidime
Cefepime
Meropenem
Ciprofloxacin
Gentamicin
Trimethoprim–sulfamethoxazole
Nitrofurantoin
Vancomycin
C. difficile infection
Antibiotic toxicity
Model
0.00
0.25
0.50
0.75
1.00
Recall
Test year range
2008 – 2010
2011 – 2013
2014 – 2016
2017 – 2019

107

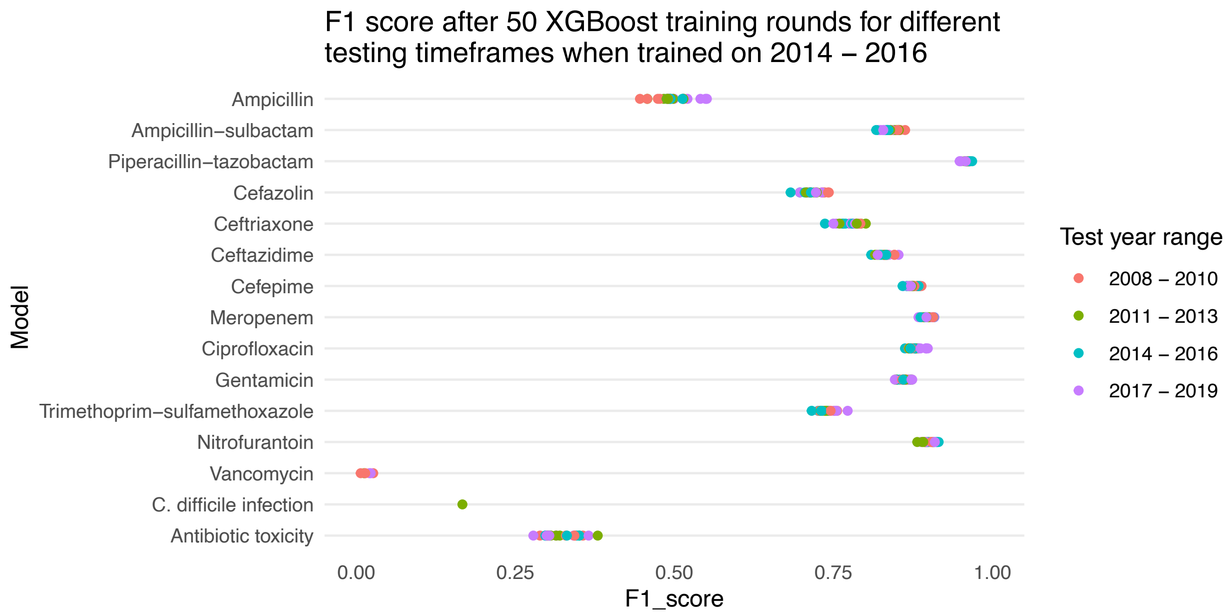
F1 score after 50 XGBoost training rounds for different testing timeframes when trained on 2014 – 2016
Ampicillin
Ampicillin–sulbactam
Piperacillin–tazobactam
Cefazolin
Ceftriaxone
Ceftazidime
Cefepime
Meropenem
Ciprofloxacin
Gentamicin
Trimethoprim–sulfamethoxazole
Nitrofurantoin
Vancomycin
C. difficile infection
Antibiotic toxicity
Model
0.00
0.25
0.50
0.75
1.00
F1_score
Test year range
2008 – 2010
2011 – 2013
2014 – 2016
2017 – 2019

108

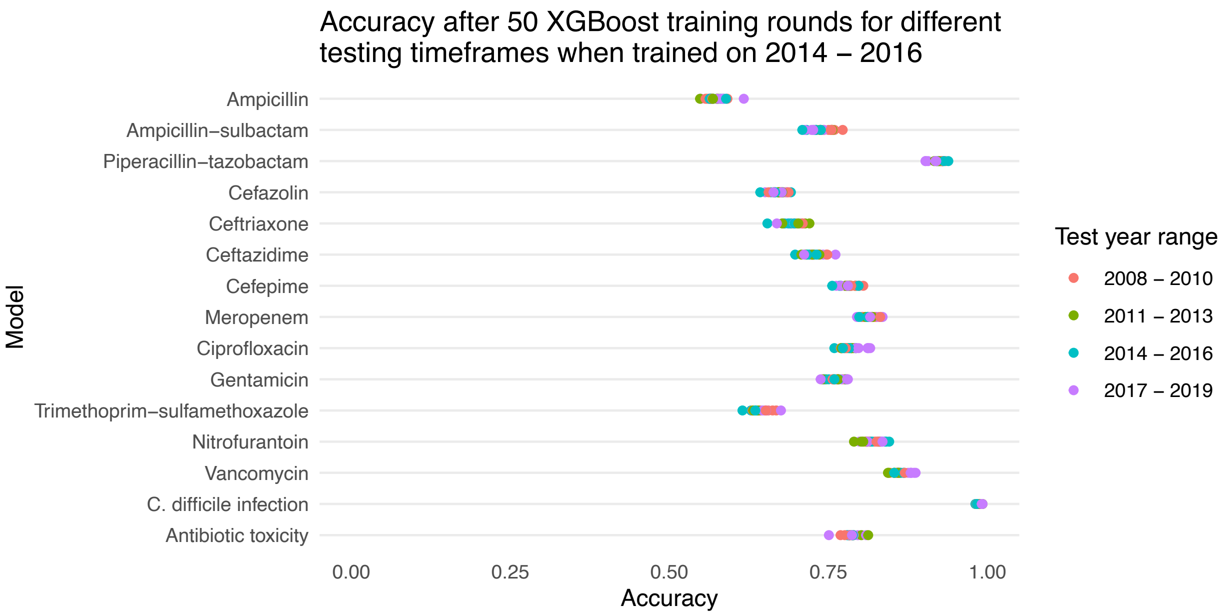
Accuracy after 50 XGBoost training rounds for different testing timeframes when trained on 2014 – 2016
Model
Ampicillin
Ampicillin–sulbactam
Piperacillin–tazobactam
Cefazolin
Ceftriaxone
Ceftazidime
Cefepime
Meropenem
Ciprofloxacin
Gentamicin
Trimethoprim–sulfamethoxazole
Nitrofurantoin
Vancomycin
C. difficile infection
Antibiotic toxicity
0.00
0.25
0.50
0.75
1.00
Accuracy
Test year range
2008 – 2010
2011 – 2013
2014 – 2016
2017 – 2019

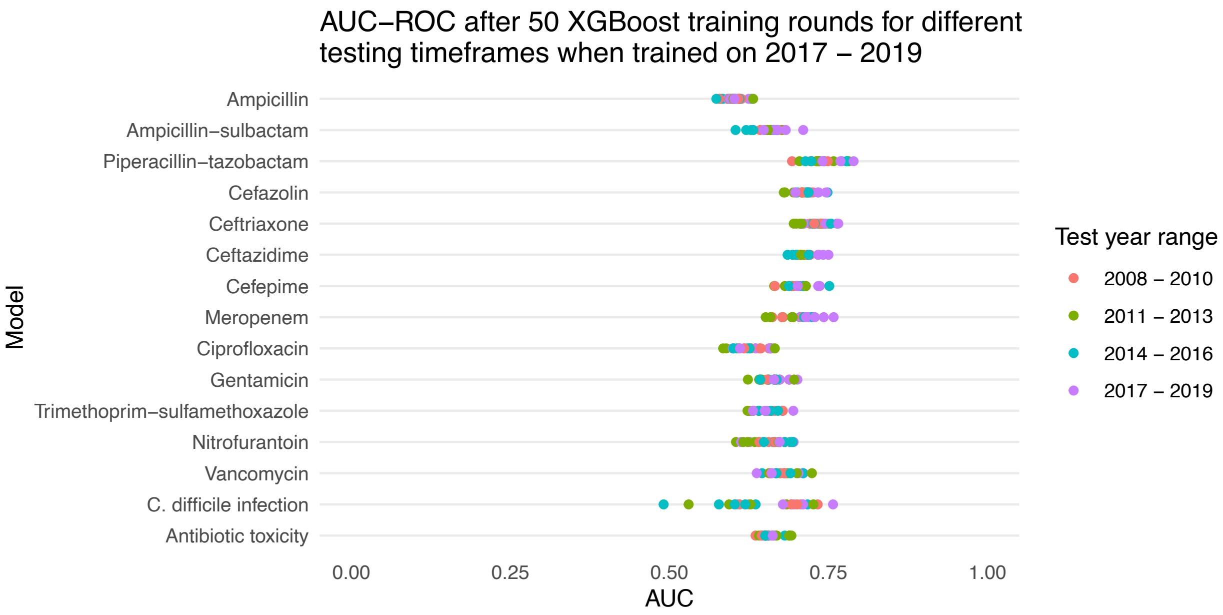
AUC–ROC after 50 XGBoost training rounds for different testing timeframes when trained on 2017 – 2019
Model
Ampicillin
Ampicillin–sulbactam
Piperacillin–tazobactam
Cefazolin
Ceftriaxone
Ceftazidime
Cefepime
Meropenem
Ciprofloxacin
Gentamicin
Trimethoprim–sulfamethoxazole
Nitrofurantoin
Vancomycin
C. difficile infection
Antibiotic toxicity
0.00
0.25
0.50
0.75
1.00
AUC
Test year range
2008 – 2010
2011 – 2013
2014 – 2016
2017 – 2019

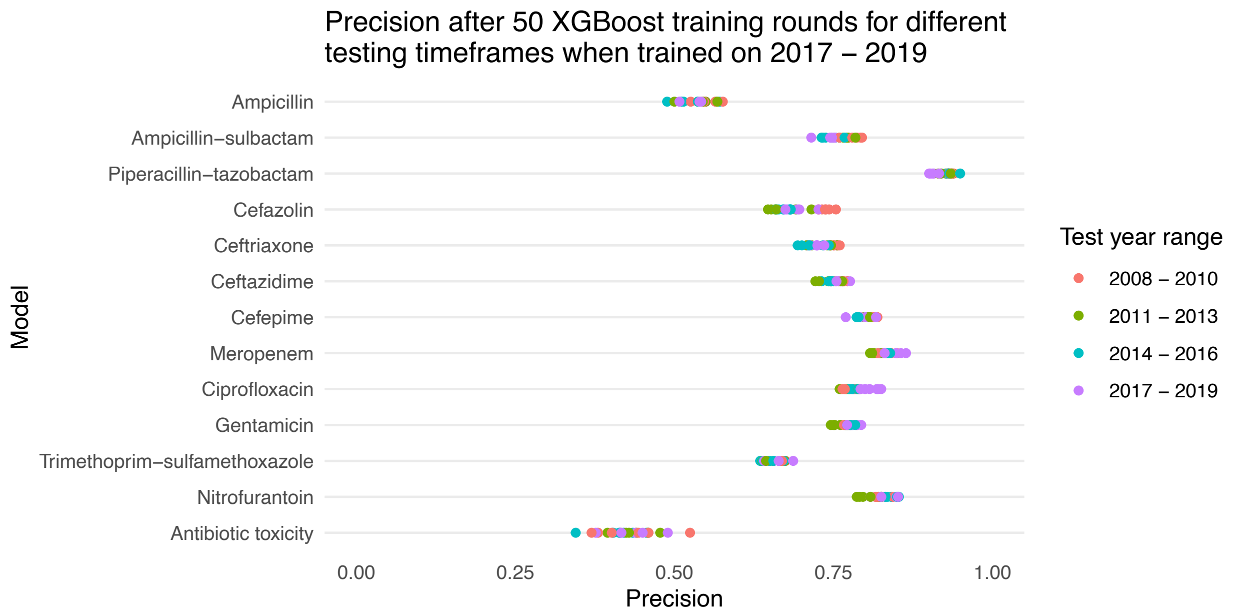
Precision after 50 XGBoost training rounds for different testing timeframes when trained on 2017 – 2019
Ampicillin
Ampicillin−sulbactam
Piperacillin−tazobactam
Cefazolin
Ceftriaxone
Ceftazidime
Cefepime
Meropenem
Ciprofloxacin
Gentamicin
Trimethoprim−sulfamethoxazole
Nitrofurantoin
Antibiotic toxicity
Model
0.00
0.25
0.50
0.75
1.00
Precision
Test year range
2008 – 2010
2011 – 2013
2014 – 2016
2017 – 2019

111

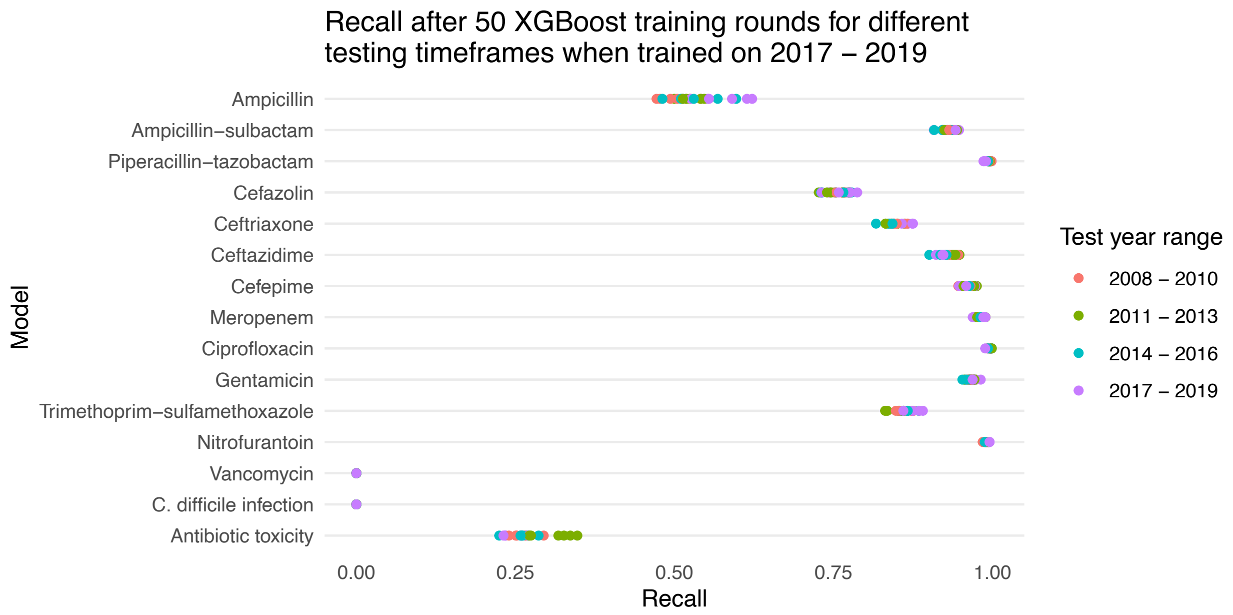
Recall after 50 XGBoost training rounds for different testing timeframes when trained on 2017 – 2019
Ampicillin
Ampicillin−sulbactam
Piperacillin−tazobactam
Cefazolin
Ceftriaxone
Ceftazidime
Cefepime
Meropenem
Ciprofloxacin
Gentamicin
Trimethoprim−sulfamethoxazole
Nitrofurantoin
Vancomycin
C. difficile infection
Antibiotic toxicity
Model
0.00
0.25
0.50
0.75
1.00
Recall
Test year range
2008 – 2010
2011 – 2013
2014 – 2016
2017 – 2019

112

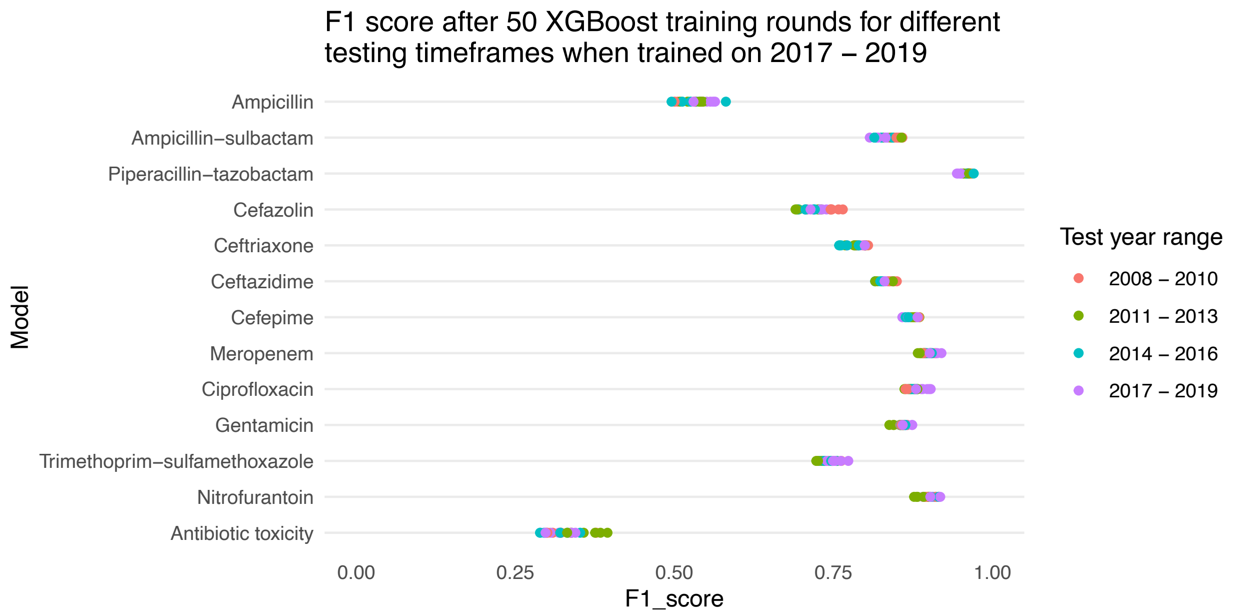

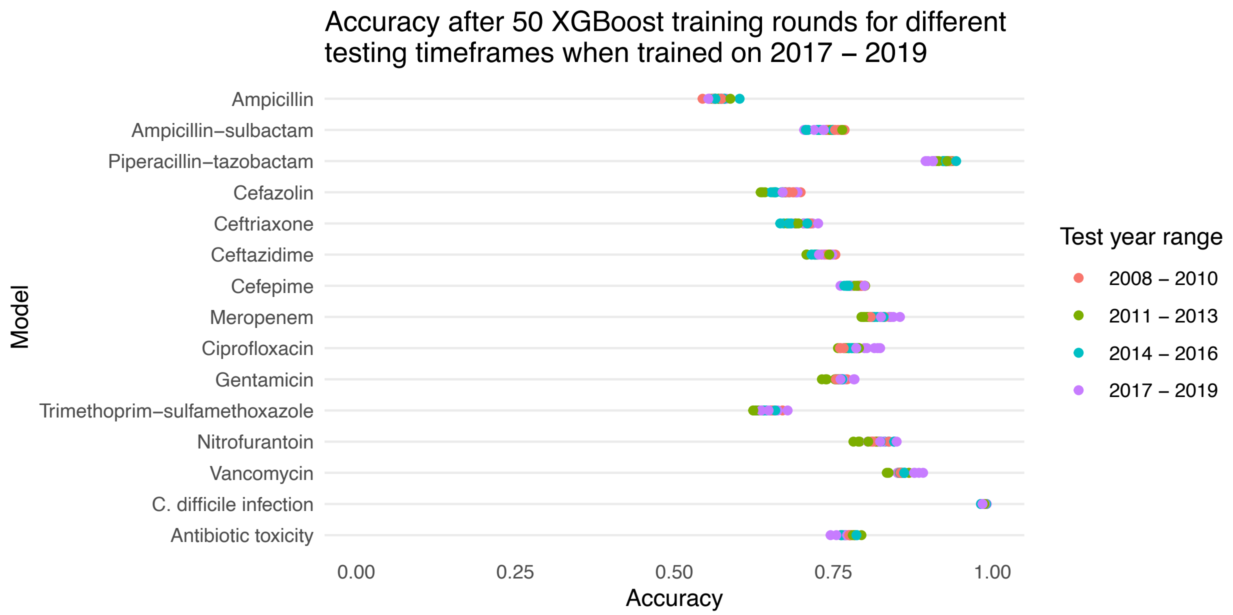

Supplementary Fig. 6: Fairness analyses

Fairness analyses of clinical prediction model performance across age, race, gender, spoken language, and marital status in six random train-test splits stratified by outcome.

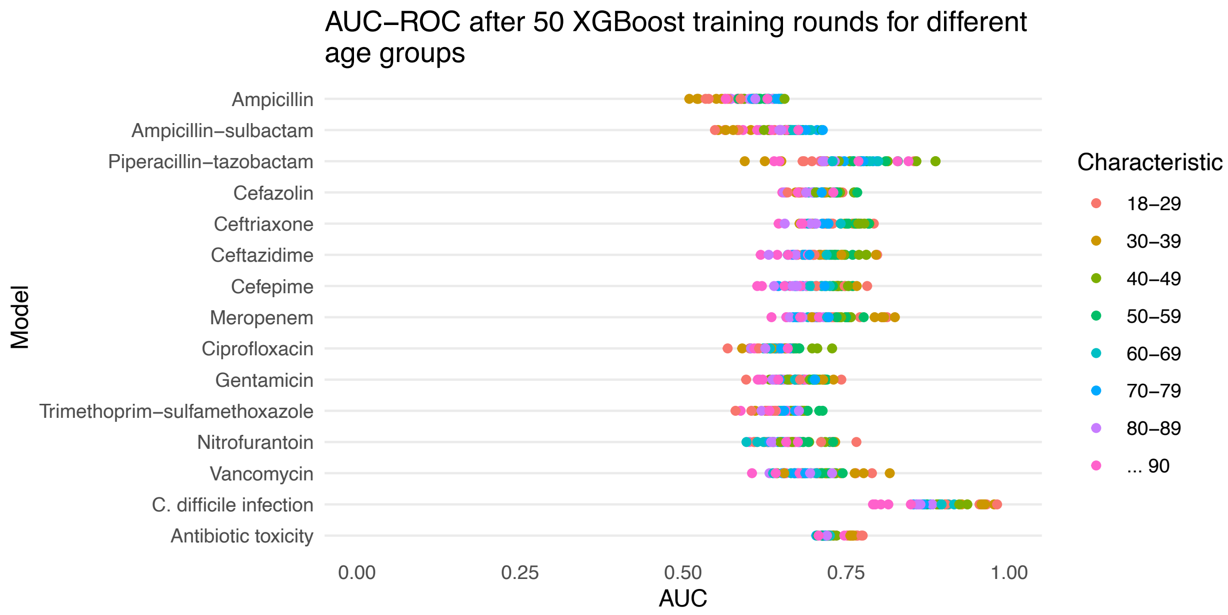
AUC−ROC after 50 XGBoost training rounds for different age groups
Ampicillin
Ampicillin−sulbactam
Piperacillin−tazobactam
Cefazolin
Ceftriaxone
Ceftazidime
Cefepime
Meropenem
Ciprofloxacin
Gentamicin
Trimethoprim−sulfamethoxazole
Nitrofurantoin
Vancomycin
C. difficile infection
Antibiotic toxicity
Model
0.00
0.25
0.50
0.75
1.00
AUC
Characteristic
18–29
30–39
40–49
50–59
60–69
70–79
80–89
... 90

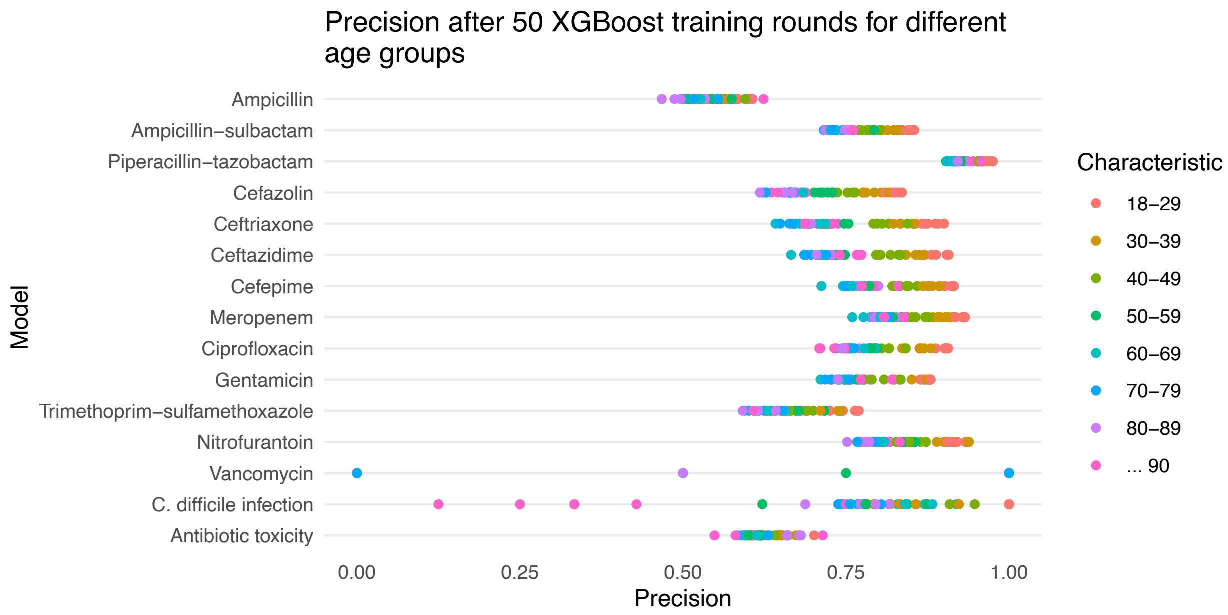
Precision after 50 XGBoost training rounds for different age groups
Ampicillin
Ampicillin−sulbactam
Piperacillin−tazobactam
Cefazolin
Ceftriaxone
Ceftazidime
Cefepime
Meropenem
Ciprofloxacin
Gentamicin
Trimethoprim−sulfamethoxazole
Nitrofurantoin
Vancomycin
C. difficile infection
Antibiotic toxicity
Model
0.00
0.25
0.50
0.75
1.00
Precision
Characteristic
18–29
30–39
40–49
50–59
60–69
70–79
80–89
... 90

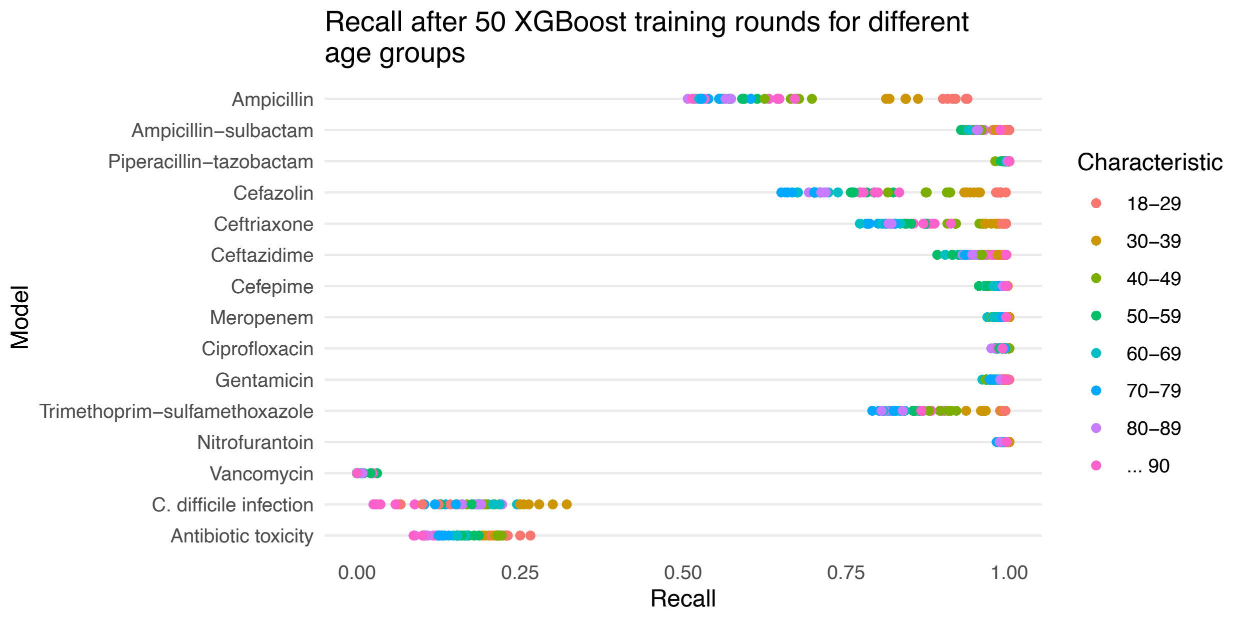
Recall after 50 XGBoost training rounds for different age groups
Ampicillin
Ampicillin−sulbactam
Piperacillin−tazobactam
Cefazolin
Ceftriaxone
Ceftazidime
Cefepime
Meropenem
Ciprofloxacin
Gentamicin
Trimethoprim−sulfamethoxazole
Nitrofurantoin
Vancomycin
C. difficile infection
Antibiotic toxicity
Model
0.00
0.25
0.50
0.75
1.00
Recall
Characteristic
18−29
30−39
40−49
50−59
60−69
70−79
80−89
... 90

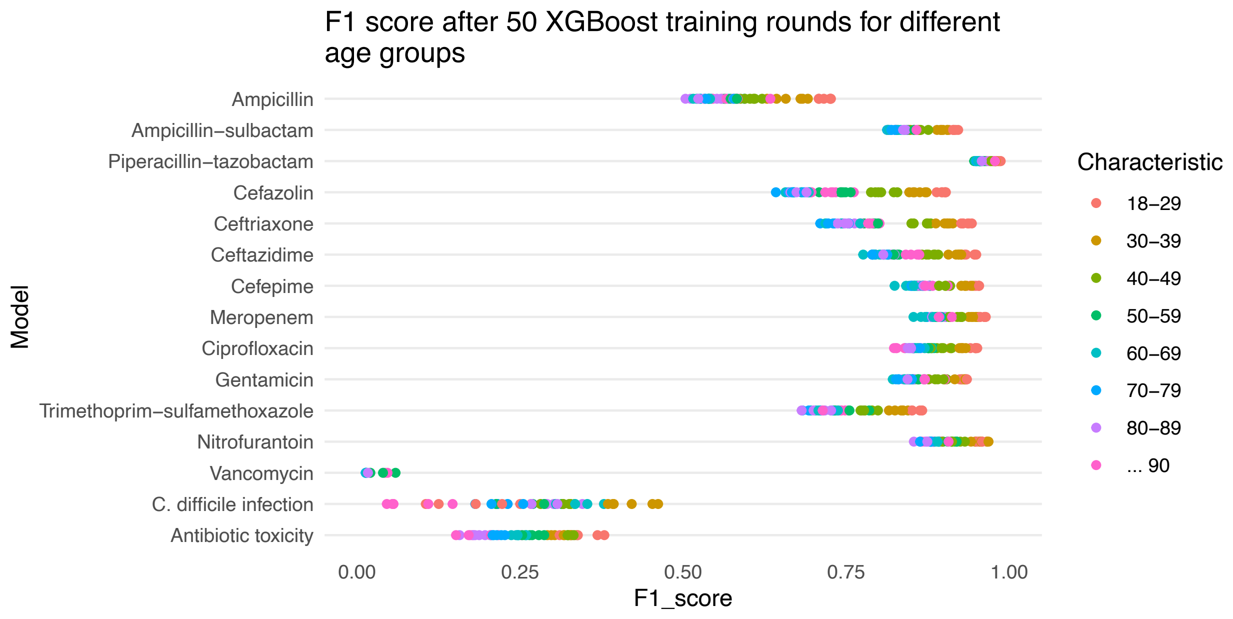
F1 score after 50 XGBoost training rounds for different age groups
Ampicillin
Ampicillin−sulbactam
Piperacillin−tazobactam
Cefazolin
Ceftriaxone
Ceftazidime
Cefepime
Meropenem
Ciprofloxacin
Gentamicin
Trimethoprim−sulfamethoxazole
Nitrofurantoin
Vancomycin
C. difficile infection
Antibiotic toxicity
Model
0.00
0.25
0.50
0.75
1.00
F1_score
Characteristic
18−29
30−39
40−49
50−59
60−69
70−79
80−89
... 90

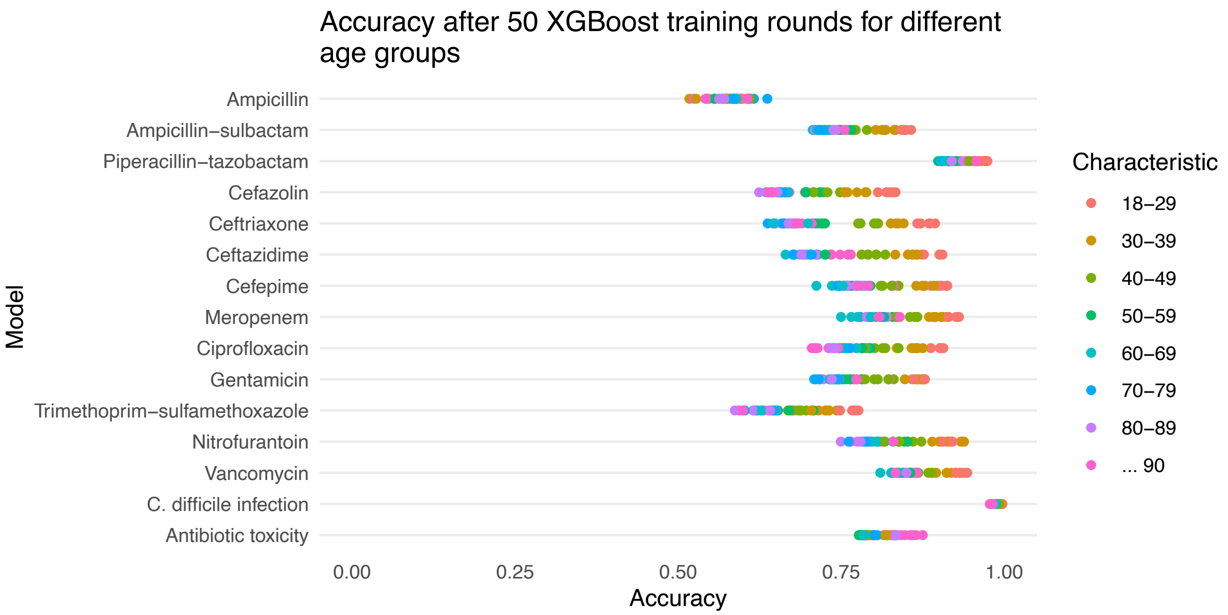
Accuracy after 50 XGBoost training rounds for different age groups
Ampicillin
Ampicillin–sulbactam
Piperacillin–tazobactam
Cefazolin
Ceftriaxone
Ceftazidime
Cefepime
Meropenem
Ciprofloxacin
Gentamicin
Trimethoprim–sulfamethoxazole
Nitrofurantoin
Vancomycin
C. difficile infection
Antibiotic toxicity
Model
0.00
0.25
0.50
0.75
1.00
Accuracy
Characteristic
18–29
30–39
40–49
50–59
60–69
70–79
80–89
... 90

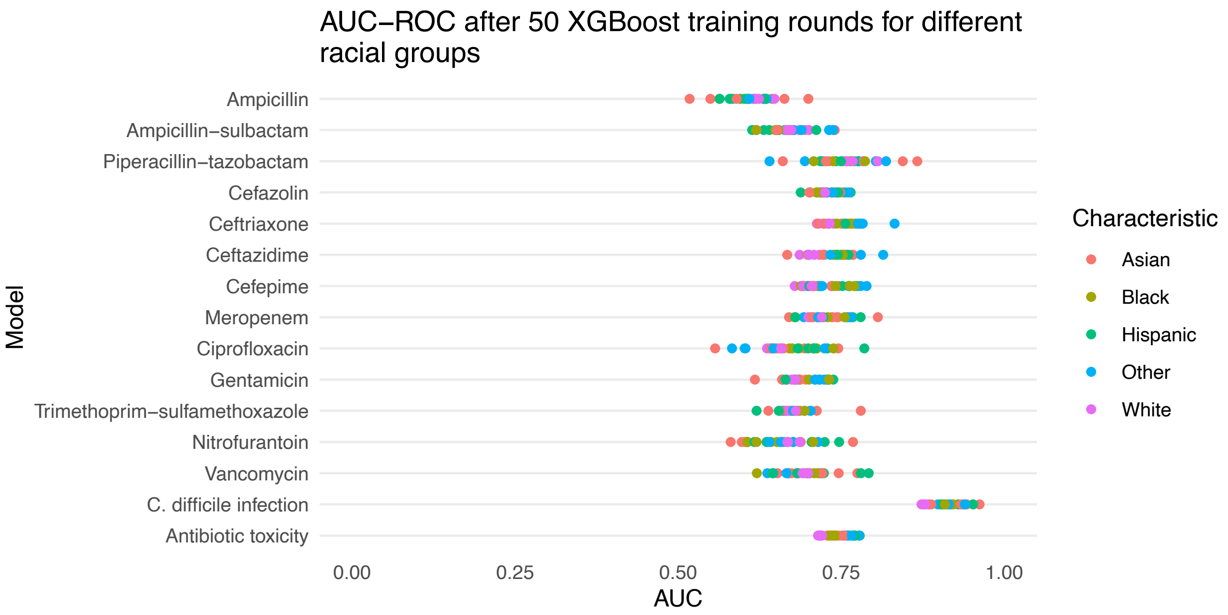
AUC–ROC after 50 XGBoost training rounds for different racial groups
Ampicillin
Ampicillin–sulbactam
Piperacillin–tazobactam
Cefazolin
Ceftriaxone
Ceftazidime
Cefepime
Meropenem
Ciprofloxacin
Gentamicin
Trimethoprim–sulfamethoxazole
Nitrofurantoin
Vancomycin
C. difficile infection
Antibiotic toxicity
Model
0.00
0.25
0.50
0.75
1.00
AUC
Characteristic
Asian
Black
Hispanic
Other
White

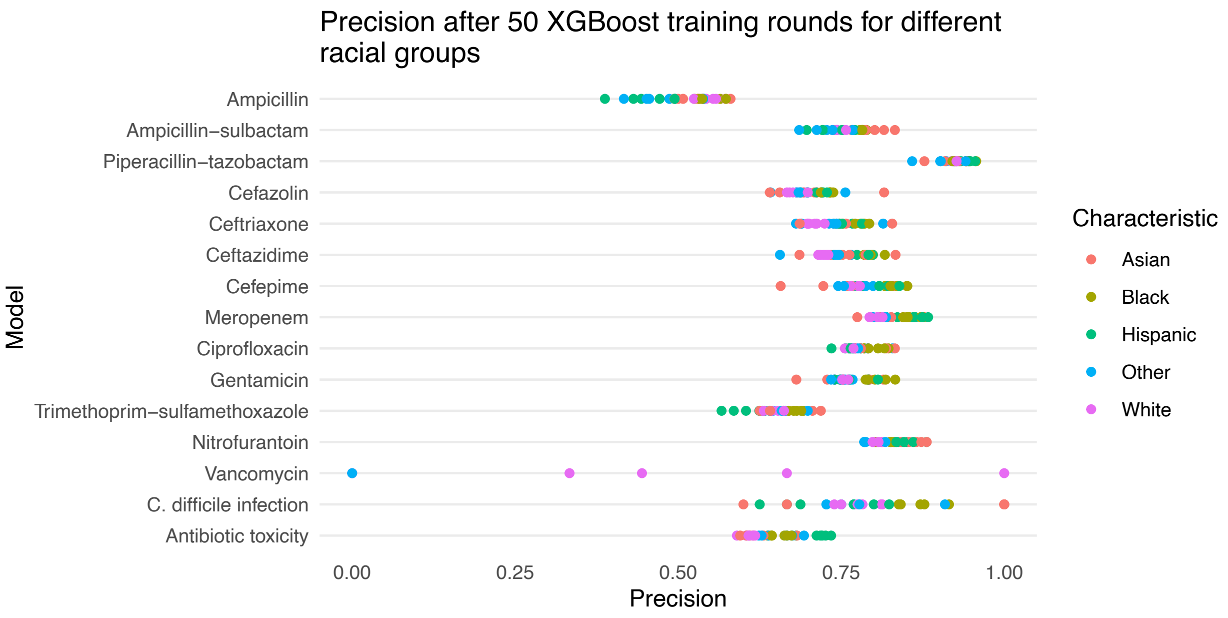
Precision after 50 XGBoost training rounds for different racial groups
Ampicillin
Ampicillin–sulbactam
Piperacillin–tazobactam
Cefazolin
Ceftriaxone
Ceftazidime
Cefepime
Meropenem
Ciprofloxacin
Gentamicin
Trimethoprim–sulfamethoxazole
Nitrofurantoin
Vancomycin
C. difficile infection
Antibiotic toxicity
Model
0.00
0.25
0.50
0.75
1.00
Precision
Characteristic
Asian
Black
Hispanic
Other
White

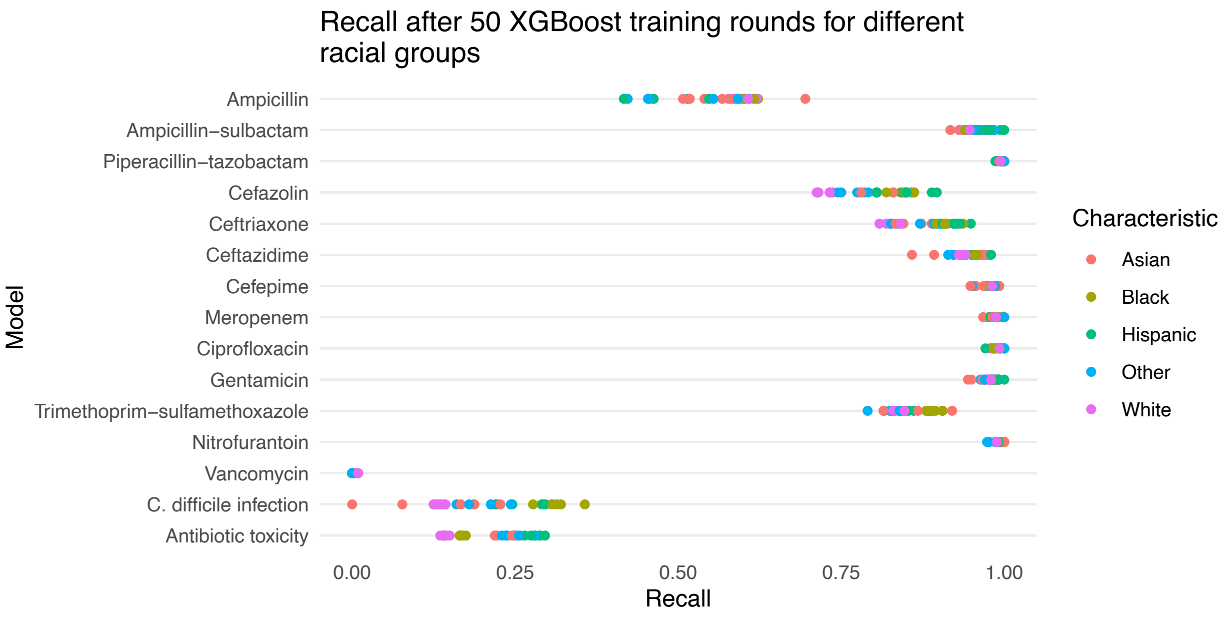
Recall after 50 XGBoost training rounds for different racial groups
Ampicillin
Ampicillin–sulbactam
Piperacillin–tazobactam
Cefazolin
Ceftriaxone
Ceftazidime
Cefepime
Meropenem
Ciprofloxacin
Gentamicin
Trimethoprim–sulfamethoxazole
Nitrofurantoin
Vancomycin
C. difficile infection
Antibiotic toxicity
Model
0.00
0.25
0.50
0.75
1.00
Recall
Characteristic
Asian
Black
Hispanic
Other
White

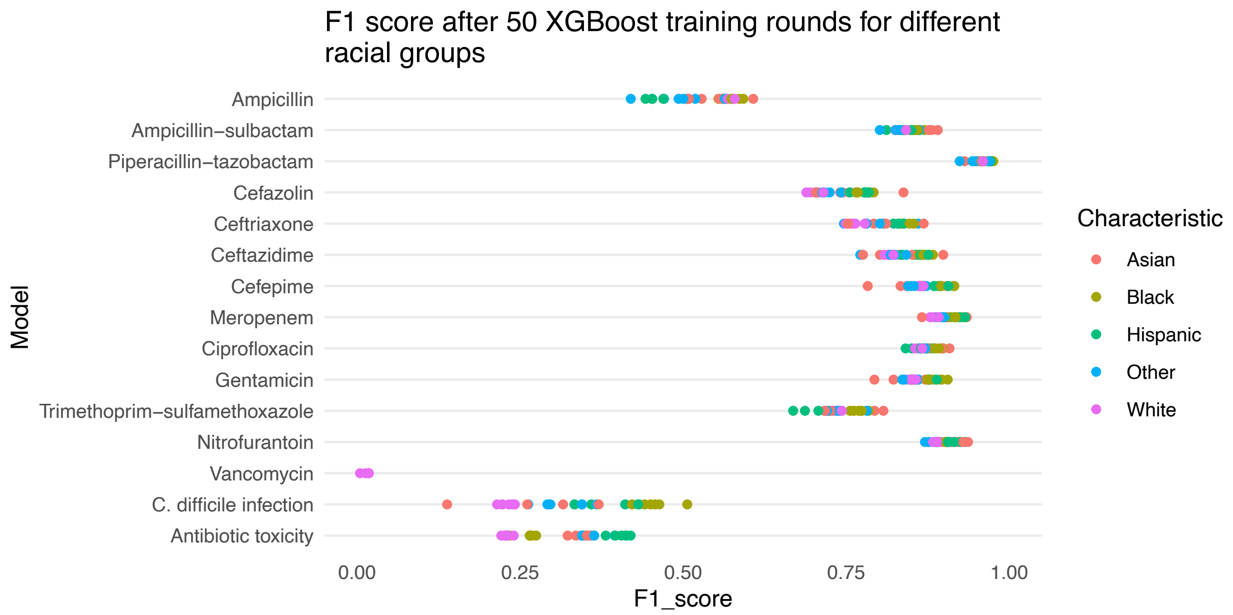

F1 score after 50 XGBoost training rounds for different racial groups
Ampicillin
Ampicillin–sulbactam
Piperacillin–tazobactam
Cefazolin
Ceftriaxone
Ceftazidime
Cefepime
Meropenem
Ciprofloxacin
Gentamicin
Trimethoprim–sulfamethoxazole
Nitrofurantoin
Vancomycin
C. difficile infection
Antibiotic toxicity
Model
0.00
0.25
0.50
0.75
1.00
F1_score
Characteristic
Asian
Black
Hispanic
Other
White

128

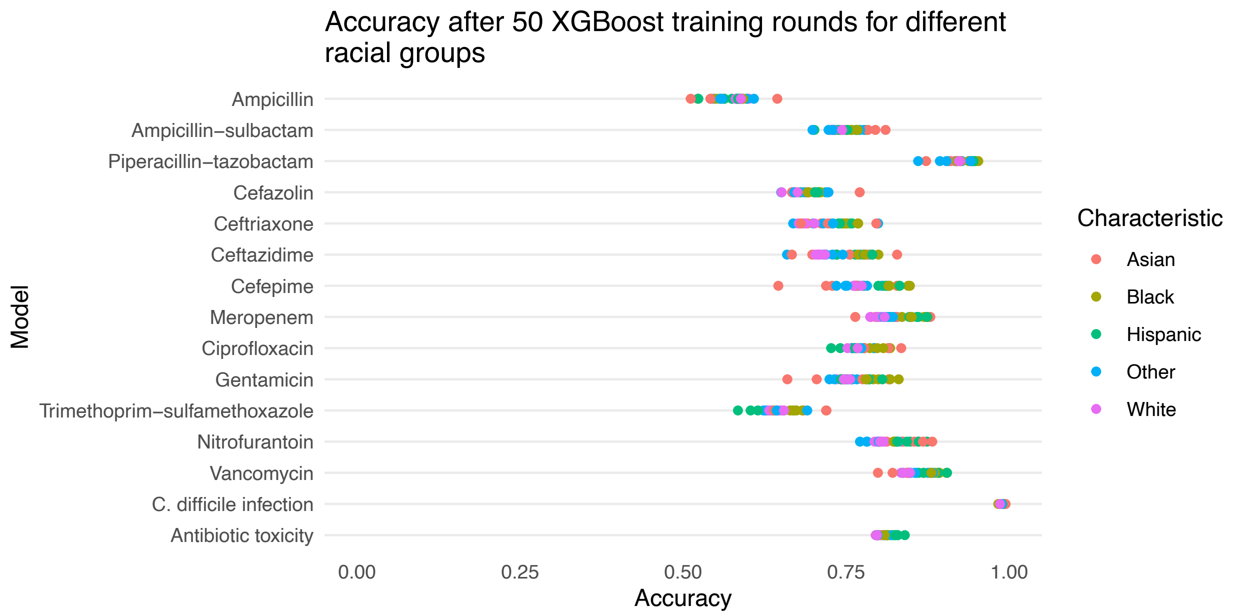

Accuracy after 50 XGBoost training rounds for different racial groups
Ampicillin
Ampicillin–sulbactam
Piperacillin–tazobactam
Cefazolin
Ceftriaxone
Ceftazidime
Cefepime
Meropenem
Ciprofloxacin
Gentamicin
Trimethoprim–sulfamethoxazole
Nitrofurantoin
Vancomycin
C. difficile infection
Antibiotic toxicity
Model
0.00
0.25
0.50
0.75
1.00
Accuracy
Characteristic
Asian
Black
Hispanic
Other
White

129

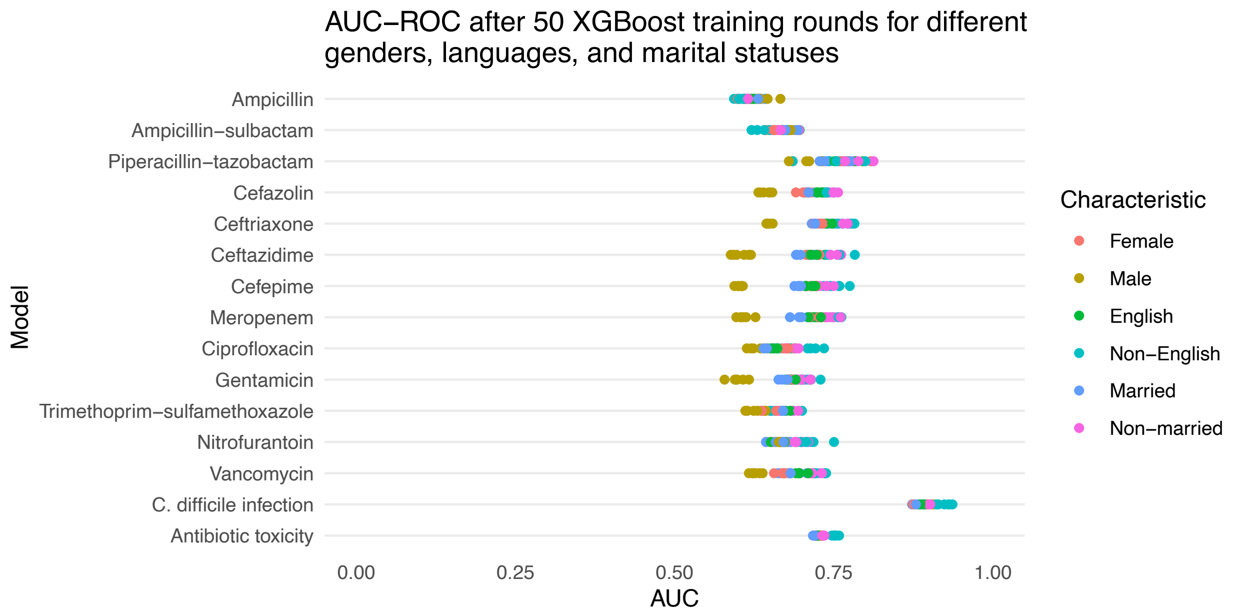
AUC−ROC after 50 XGBoost training rounds for different genders, languages, and marital statuses
Model
Ampicillin
Ampicillin−sulbactam
Piperacillin−tazobactam
Cefazolin
Ceftriaxone
Ceftazidime
Cefepime
Meropenem
Ciprofloxacin
Gentamicin
Trimethoprim−sulfamethoxazole
Nitrofurantoin
Vancomycin
C. difficile infection
Antibiotic toxicity
0.00
0.25
0.50
0.75
1.00
AUC
Characteristic
Female
Male
English
Non−English
Married
Non−married

130

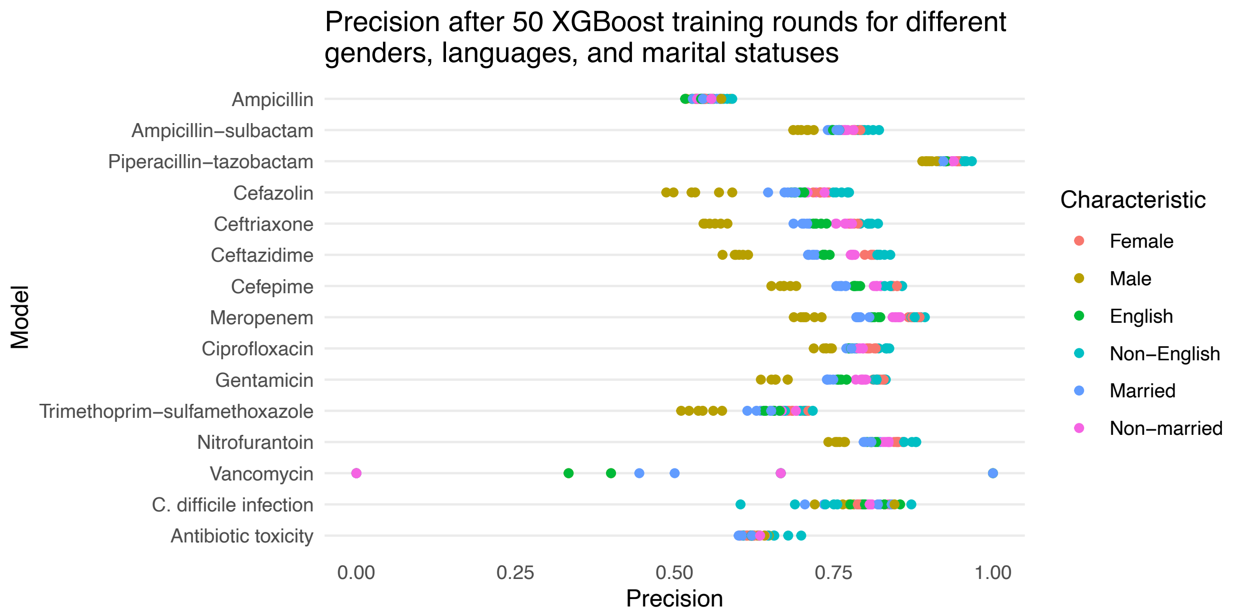
Precision after 50 XGBoost training rounds for different genders, languages, and marital statuses
Model
Ampicillin
Ampicillin−sulbactam
Piperacillin−tazobactam
Cefazolin
Ceftriaxone
Ceftazidime
Cefepime
Meropenem
Ciprofloxacin
Gentamicin
Trimethoprim−sulfamethoxazole
Nitrofurantoin
Vancomycin
C. difficile infection
Antibiotic toxicity
0.00
0.25
0.50
0.75
1.00
Precision
Characteristic
Female
Male
English
Non−English
Married
Non−married

131

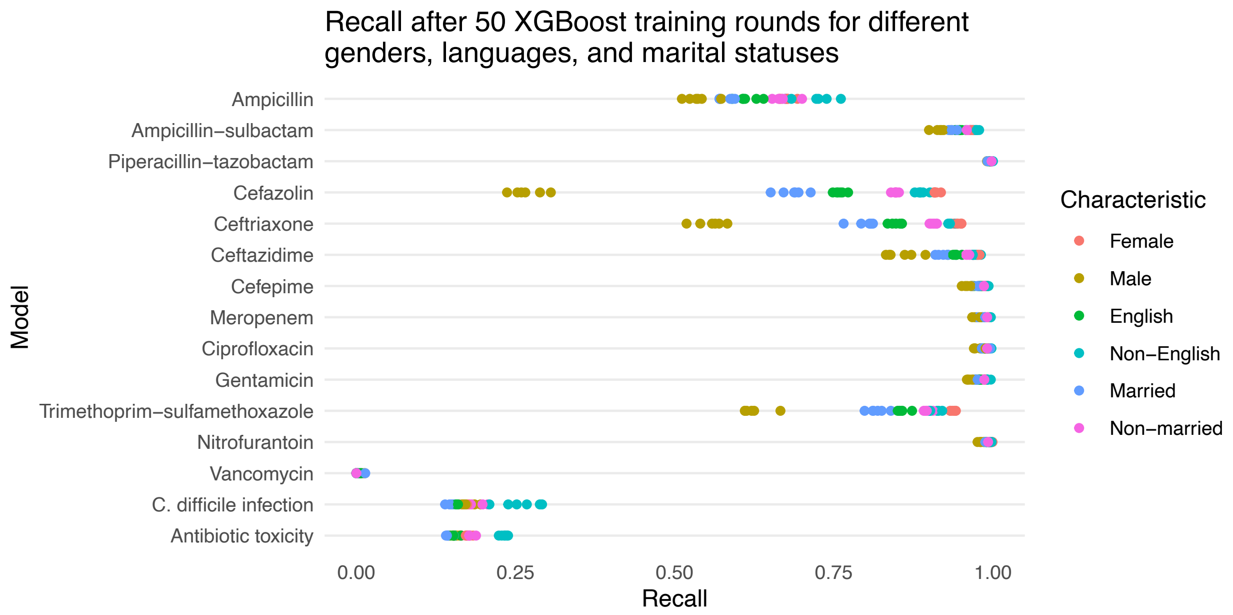
Recall after 50 XGBoost training rounds for different genders, languages, and marital statuses
Model
Ampicillin
Ampicillin−sulbactam
Piperacillin−tazobactam
Cefazolin
Ceftriaxone
Ceftazidime
Cefepime
Meropenem
Ciprofloxacin
Gentamicin
Trimethoprim−sulfamethoxazole
Nitrofurantoin
Vancomycin
C. difficile infection
Antibiotic toxicity
0.00
0.25
0.50
0.75
1.00
Recall
Characteristic
Female
Male
English
Non−English
Married
Non−married

132

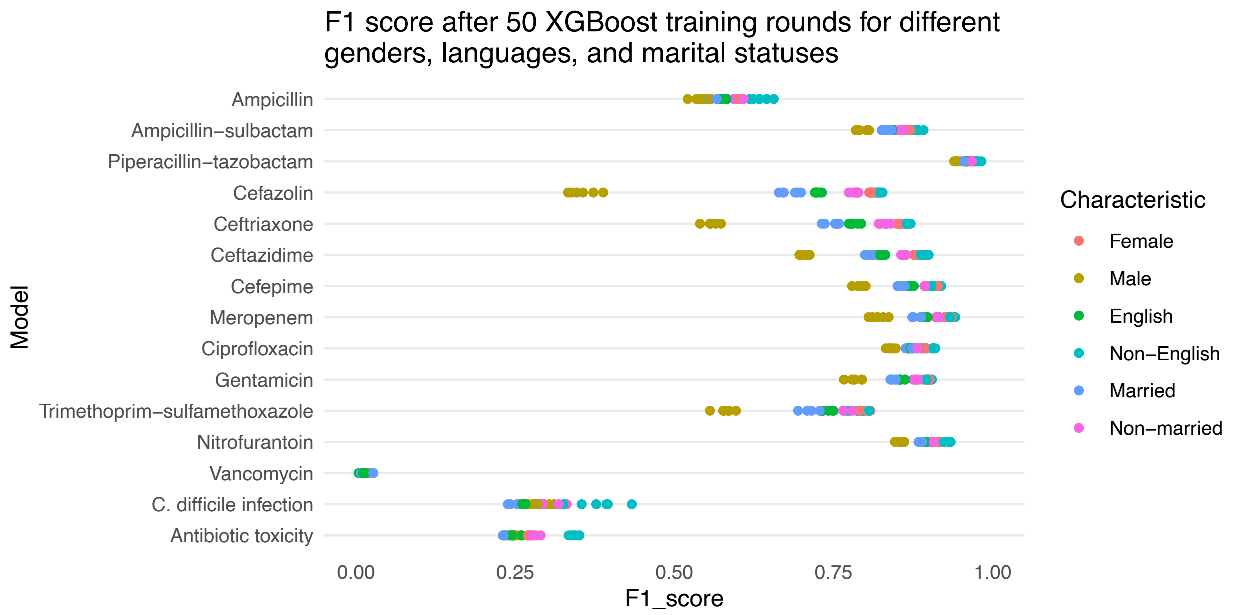
F1 score after 50 XGBoost training rounds for different genders, languages, and marital statuses
Model
Ampicillin
Ampicillin−sulbactam
Piperacillin−tazobactam
Cefazolin
Ceftriaxone
Ceftazidime
Cefepime
Meropenem
Ciprofloxacin
Gentamicin
Trimethoprim−sulfamethoxazole
Nitrofurantoin
Vancomycin
C. difficile infection
Antibiotic toxicity
0.00
0.25
0.50
0.75
1.00
F1_score
Characteristic
Female
Male
English
Non−English
Married
Non−married

133

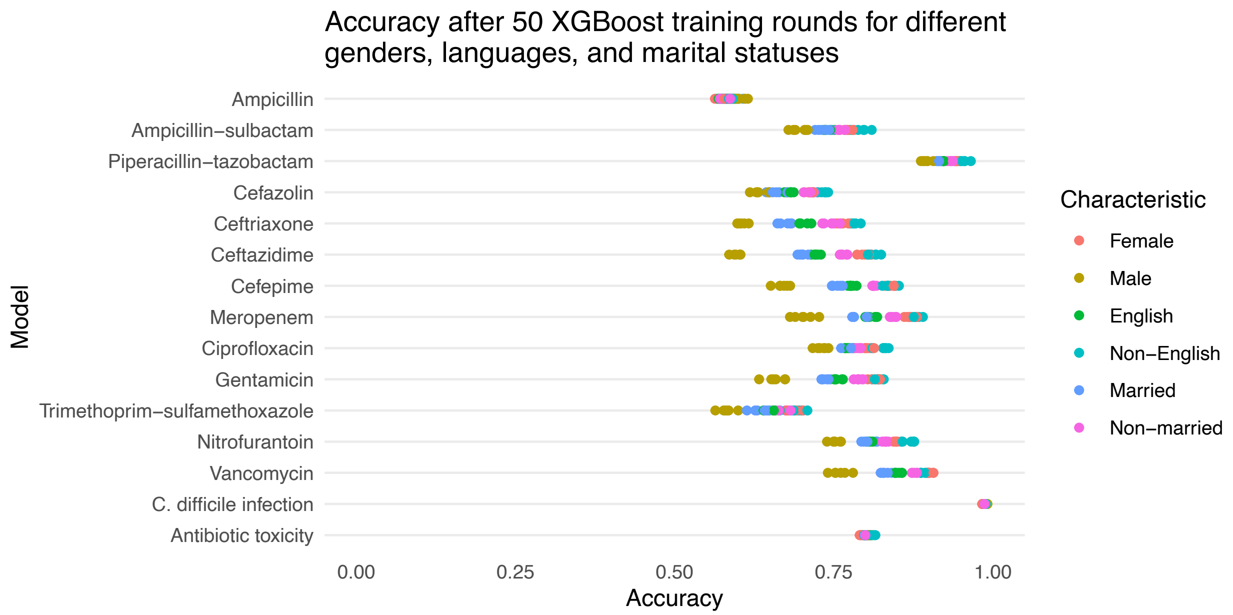

Supplementary Fig. 7: Expert weightings stratified by specialty

Expert weightings extracted by ranked logit analysis of the antibiotic choice ranking exercise, stratified by clinician participant specialties.

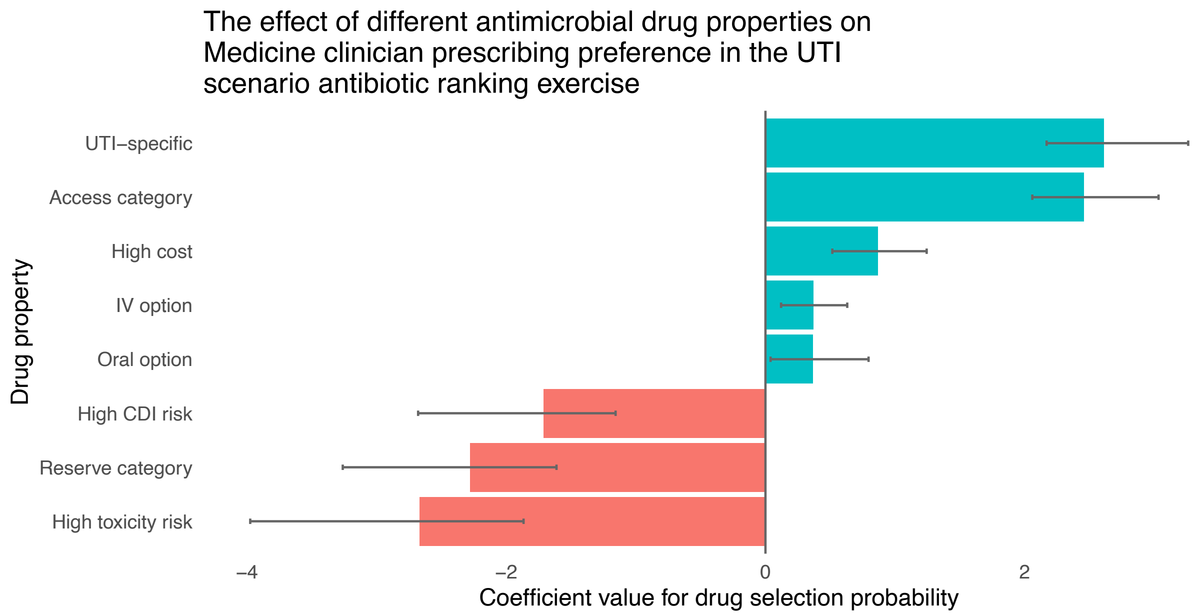

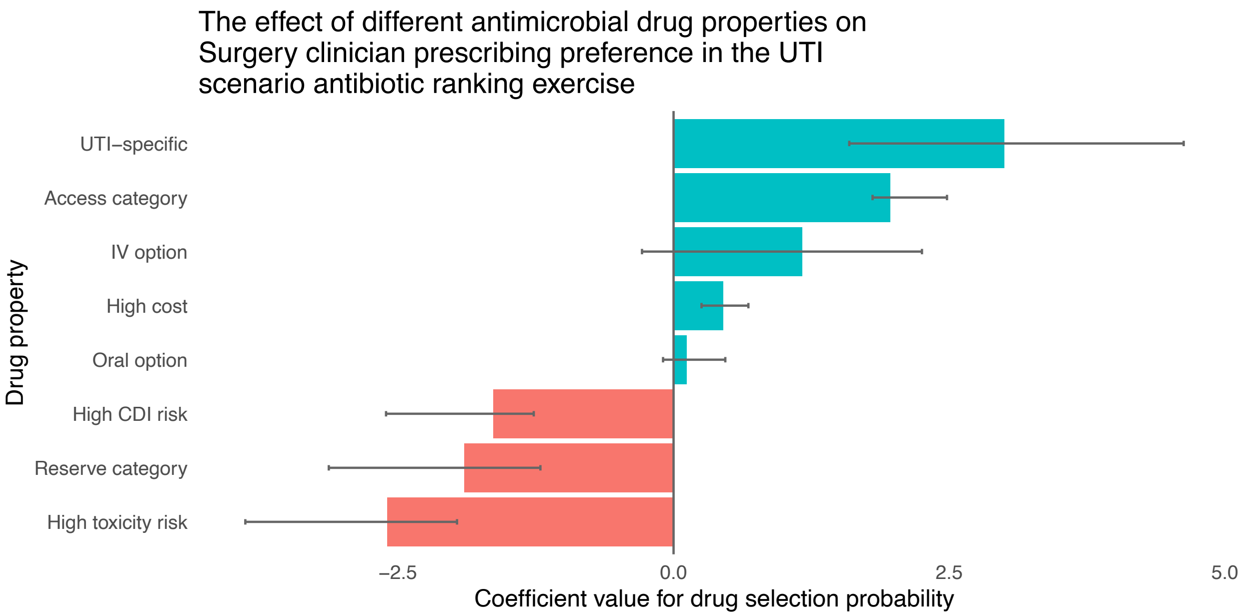
The effect of different antimicrobial drug properties on Surgery clinician prescribing preference in the UTI scenario antibiotic ranking exercise
Drug property
UTI–specific
Access category
IV option
High cost
Oral option
High CDI risk
Reserve category
High toxicity risk
−2.5
0.0
2.5
5.0
Coefficient value for drug selection probability

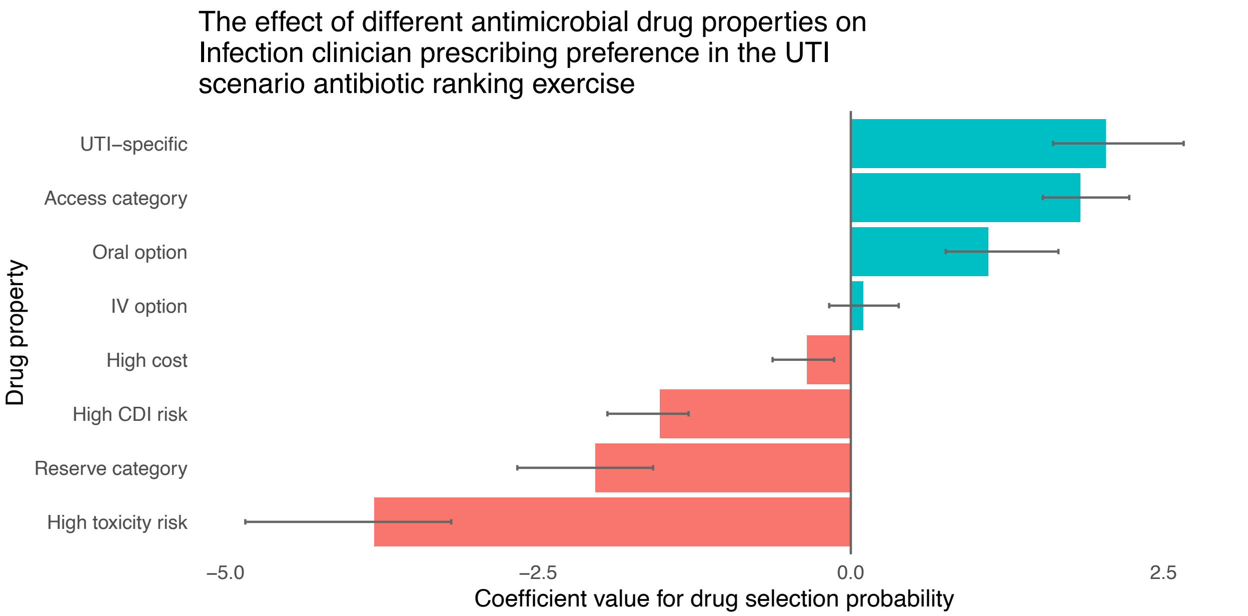
The effect of different antimicrobial drug properties on Infection clinician prescribing preference in the UTI scenario antibiotic ranking exercise
Drug property
UTI–specific
Access category
Oral option
IV option
High cost
High CDI risk
Reserve category
High toxicity risk
−5.0
−2.5
0.0
2.5
Coefficient value for drug selection probability

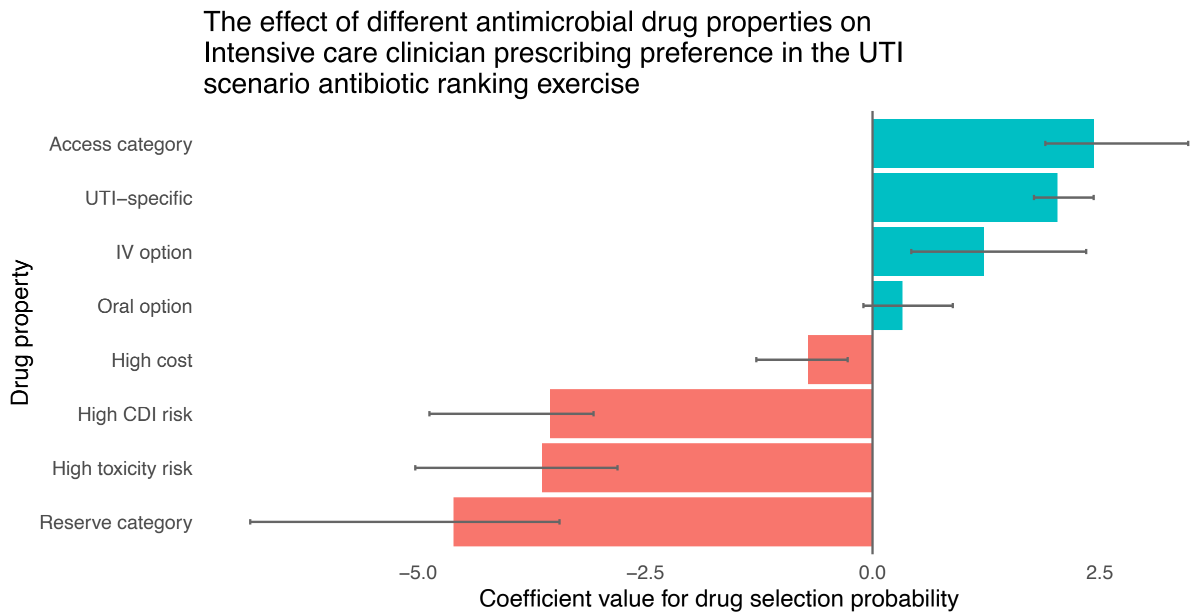

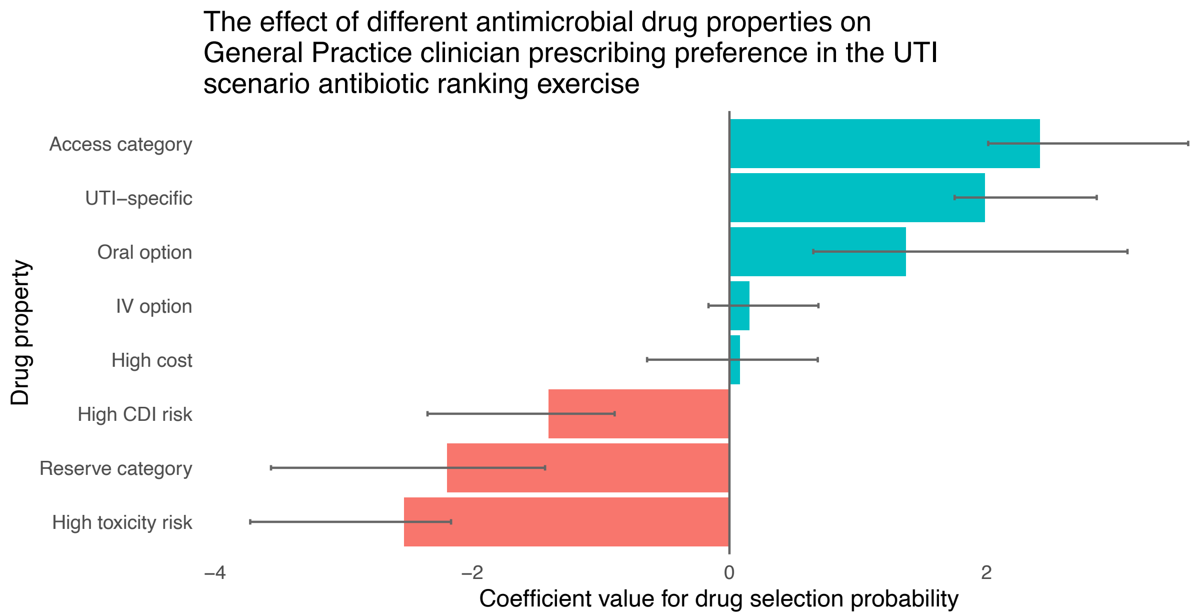

Supplementary Fig. 8: The distribution of antibiotic treatment values by specialty

The distribution of utility values across the microsimulation dataset for all single agents, stratified by the individual specialities.

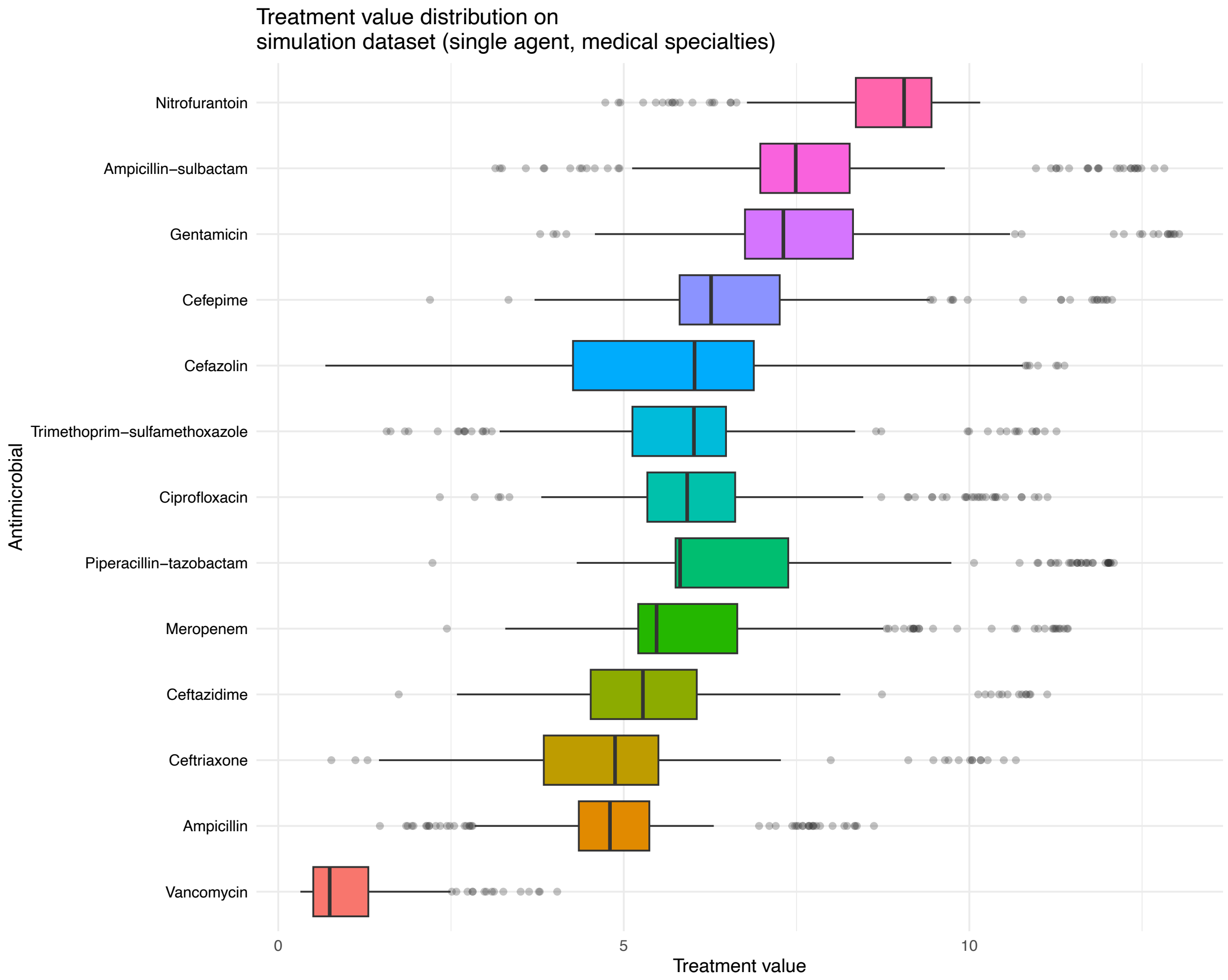
Treatment value distribution on
simulation dataset (single agent, medical specialties)
Nitrofurantoin
Ampicillin–sulbactam
Gentamicin
Cefepime
Cefazolin
Trimethoprim–sulfamethoxazole
Ciprofloxacin
Piperacillin–tazobactam
Meropenem
Ceftazidime
Ceftriaxone
Ampicillin
Vancomycin
Antimicrobial
0
5
10
Treatment value

151

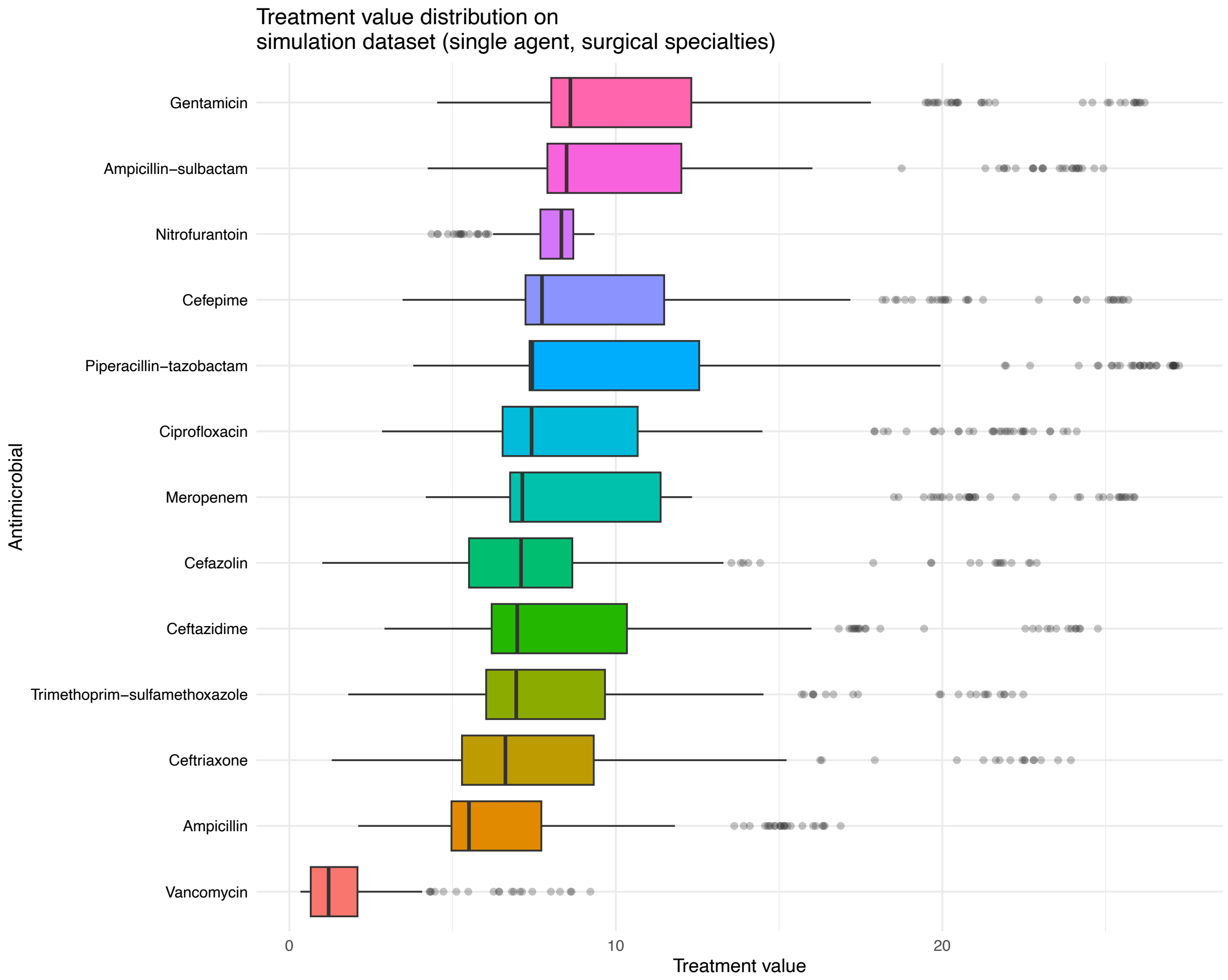
Treatment value distribution on
simulation dataset (single agent, surgical specialties)
Gentamicin
Ampicillin−sulbactam
Nitrofurantoin
Cefepime
Piperacillin−tazobactam
Ciprofloxacin
Meropenem
Cefazolin
Ceftazidime
Trimethoprim−sulfamethoxazole
Ceftriaxone
Ampicillin
Vancomycin
Antimicrobial
0
10
20
Treatment value

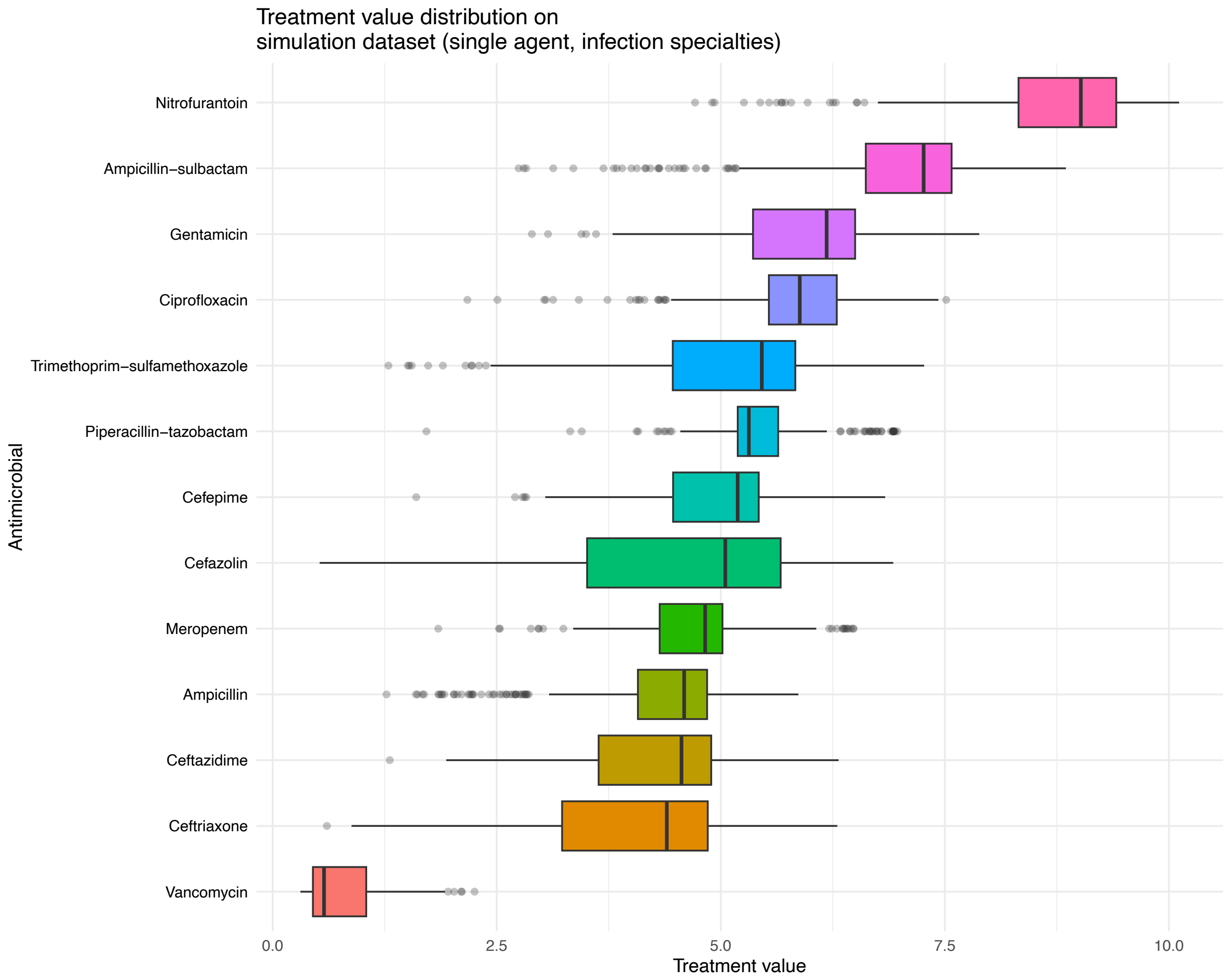

Treatment value distribution on
simulation dataset (single agent, infection specialties)
Nitrofurantoin
Ampicillin−sulbactam
Gentamicin
Ciprofloxacin
Trimethoprim−sulfamethoxazole
Piperacillin−tazobactam
Cefepime
Cefazolin
Meropenem
Ampicillin
Ceftazidime
Ceftriaxone
Vancomycin
Antimicrobial
0.0
2.5
5.0
7.5
10.0
Treatment value

153

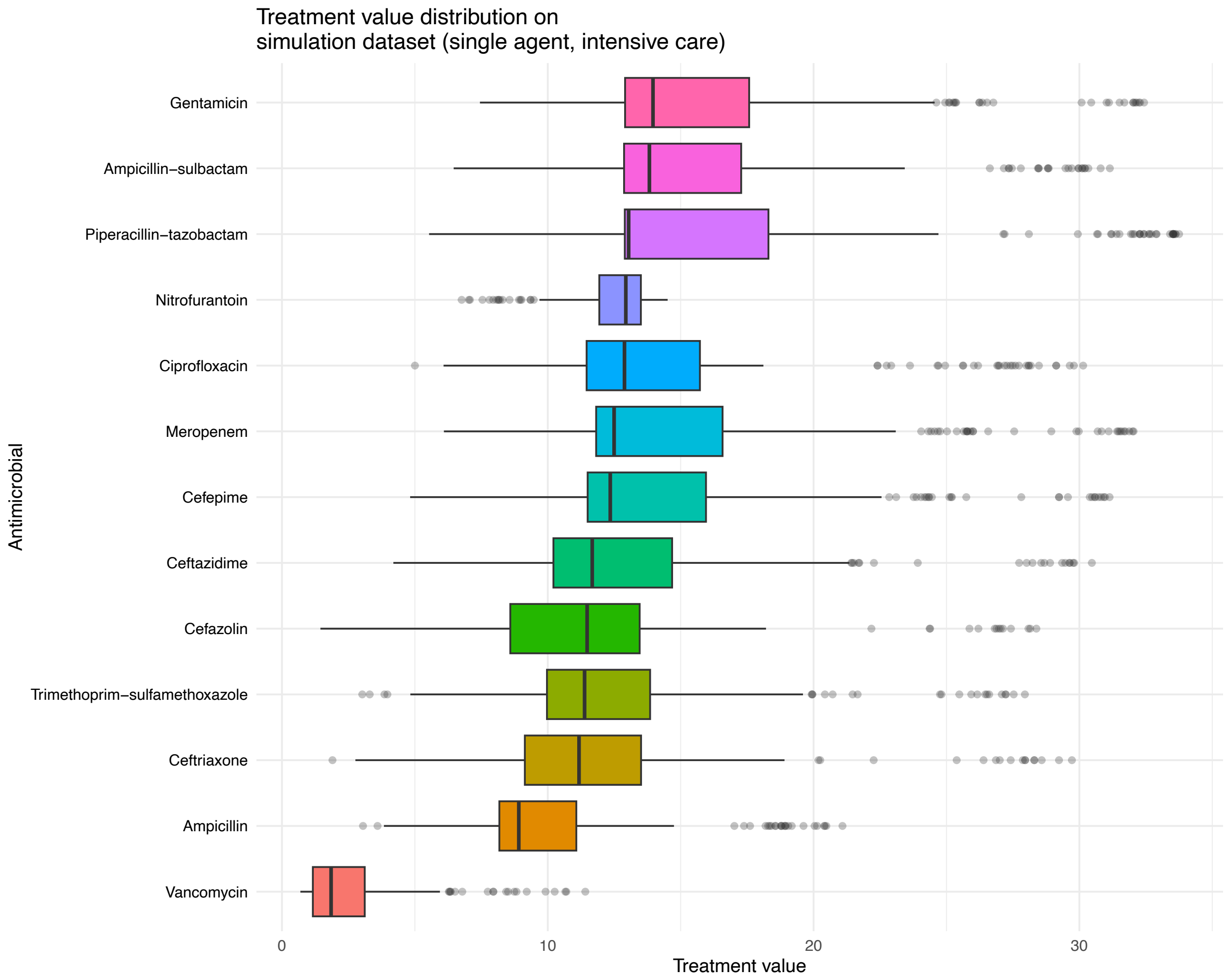
Treatment value distribution on
simulation dataset (single agent, intensive care)
Gentamicin
Ampicillin−sulbactam
Piperacillin−tazobactam
Nitrofurantoin
Ciprofloxacin
Meropenem
Cefepime
Ceftazidime
Cefazolin
Trimethoprim−sulfamethoxazole
Ceftriaxone
Ampicillin
Vancomycin
Antimicrobial
0
10
20
30
Treatment value

154

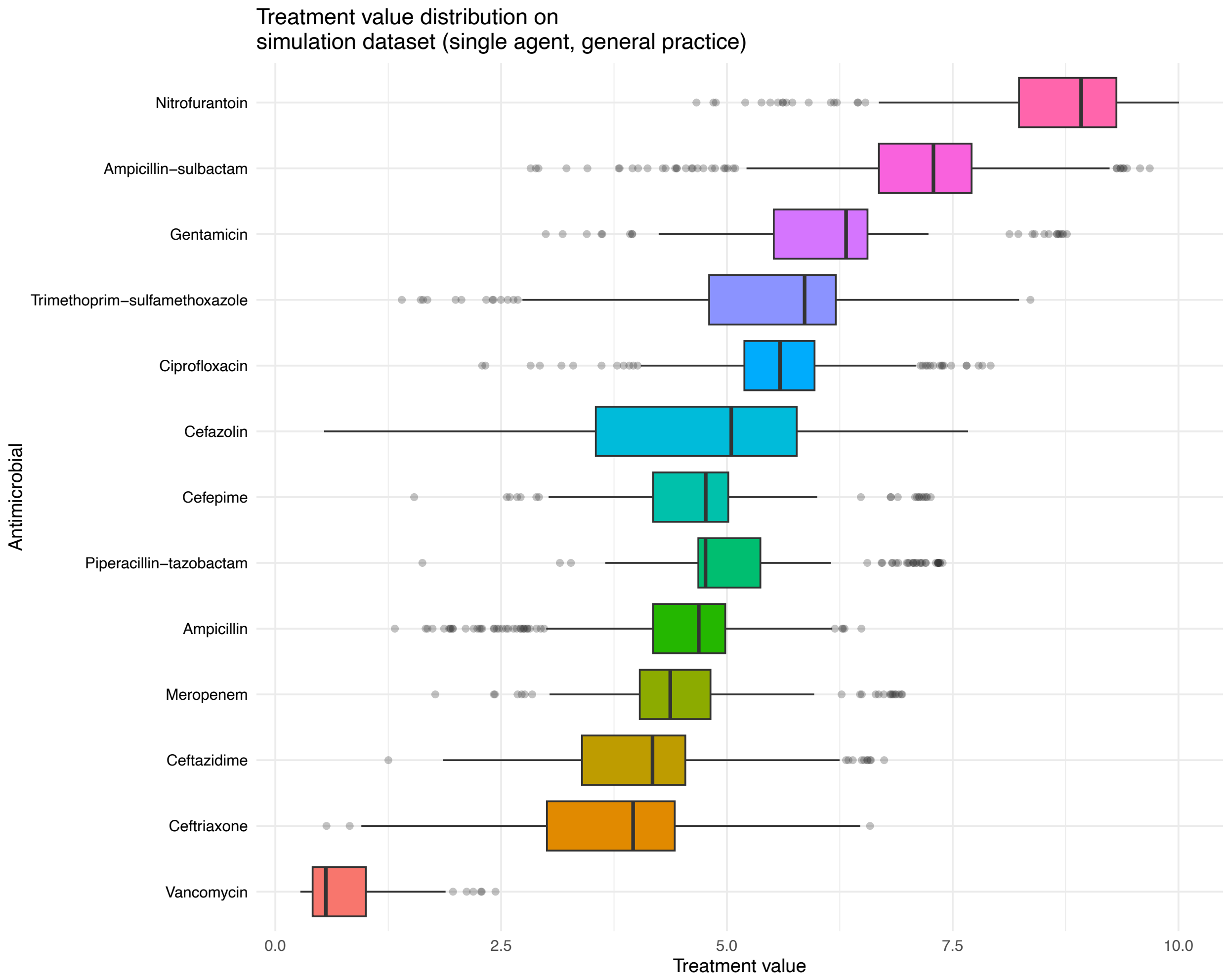

Supplementary Fig. 9: Proportion of incorrectly-targeted treatments stratified by illness severity

Plots comparing how increasing illness severity affected the failure of empirical treatment choices made by the antibiotic decision-making algorithm (ADA) and human prescribers in correctly targeting patients' urinary pathogens, by WHO Access/Watch/Reserve (AWaRe) category (top), availability of an oral option (middle), and availability of an IV option (bottom). The height of each coloured bar section represents the number of cases in which an antibiotic choice from that category (e.g., Access agent) failed to correctly target the urinary pathogen, as a proportion of the total number of choices made.

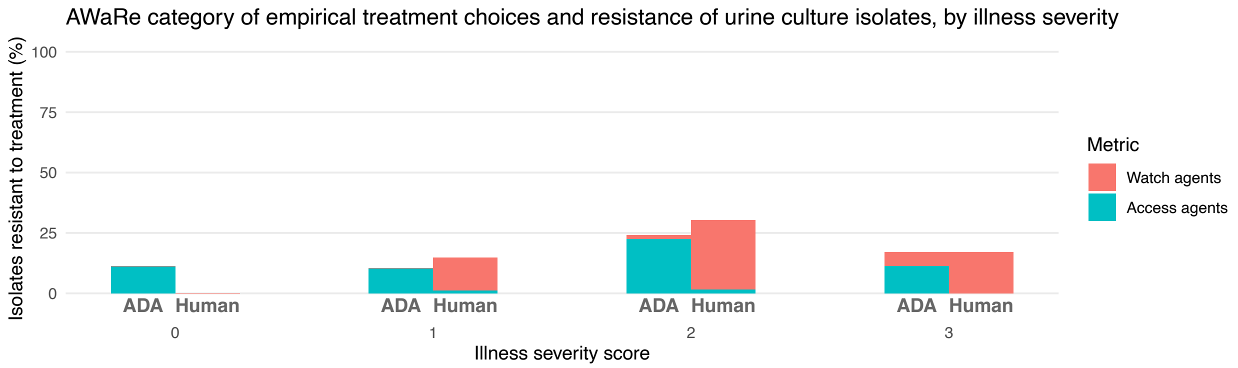

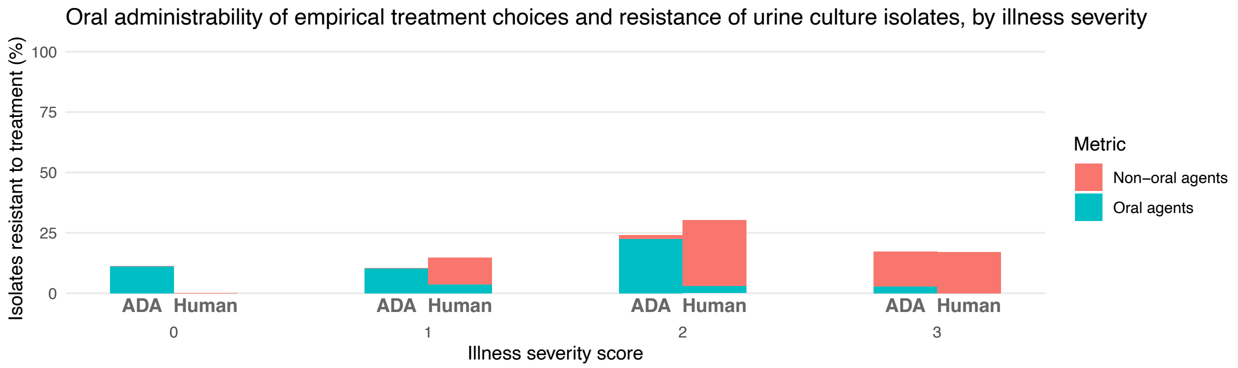

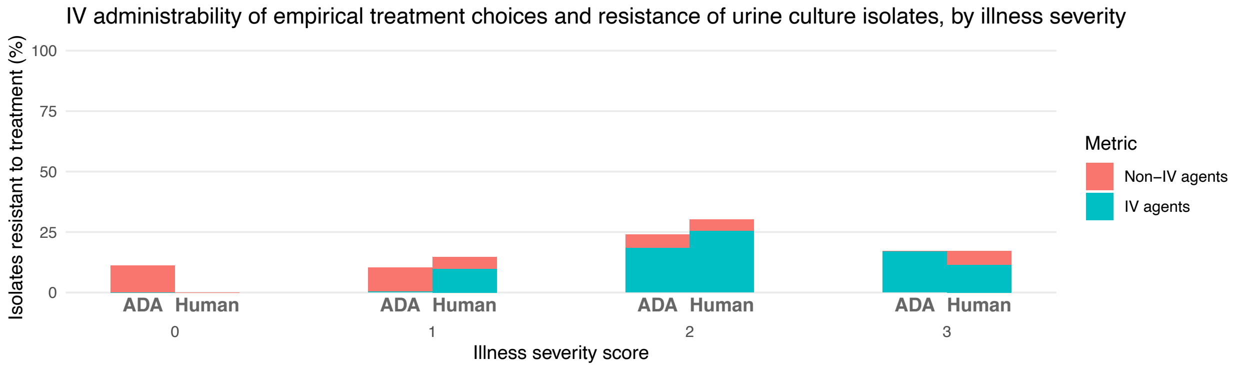

Supplementary Fig. 10: Number of susceptible results in the top six recommendations

The number of susceptible results for antibiotics in the top six antimicrobial susceptibility testing recommendations made by the antibiotic decision algorithm (ADA), overall / for WHO Access agents (top) and for oral / IV agents (bottom), compared to a standard panel of nitrofurantoin, trimethoprim-sulfamethoxazole, gentamicin, piperacillin-tazobactam, ceftriaxone, and ciprofloxacin. Median values are red/green dots, interquartile ranges are red/green lines, and all results are grey dot clusters.

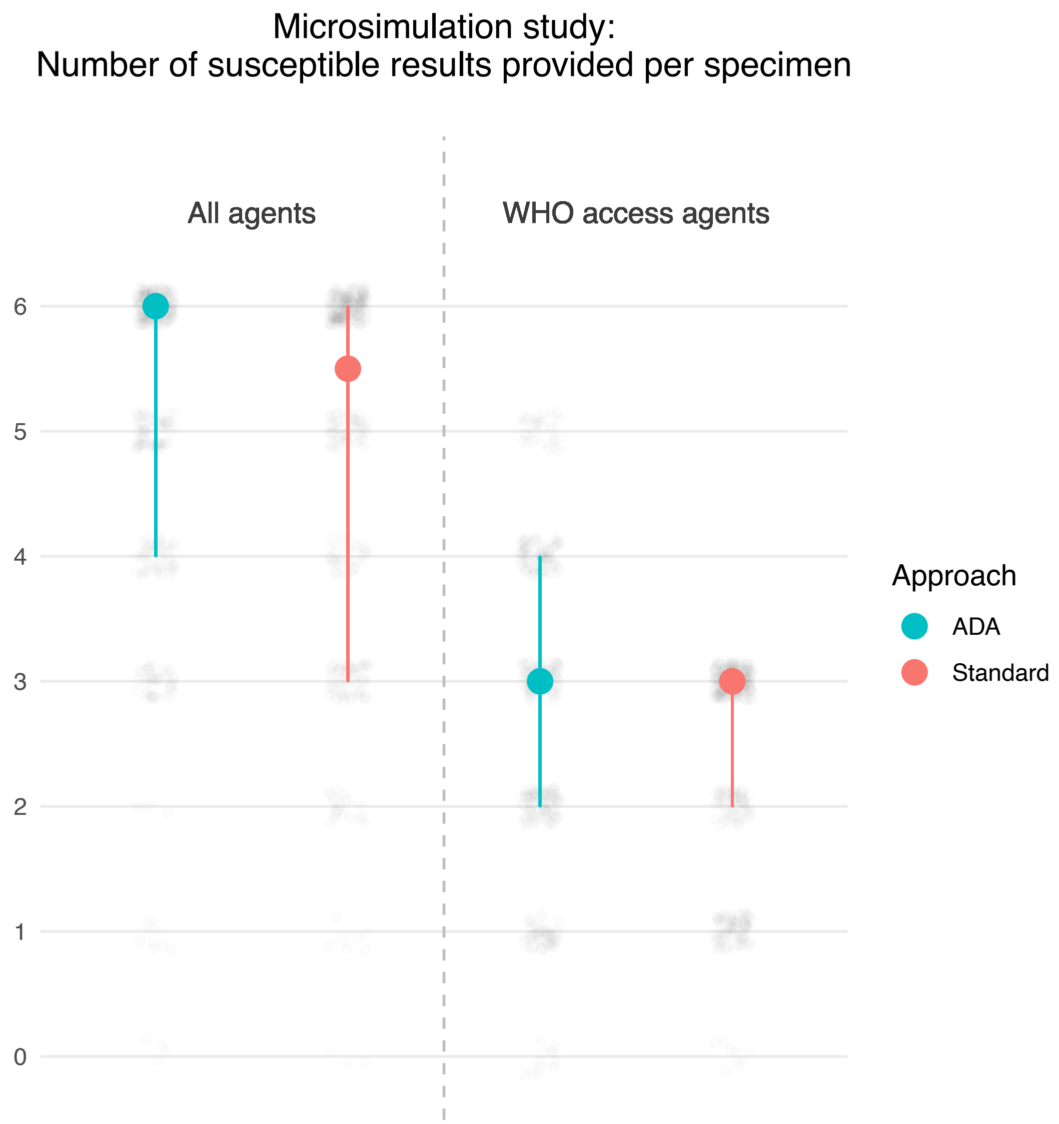
Microsimulation study:
Number of susceptible results provided per specimen
All agents
WHO access agents
6
5
4
3
2
1
0
Approach
ADA
Standard

178

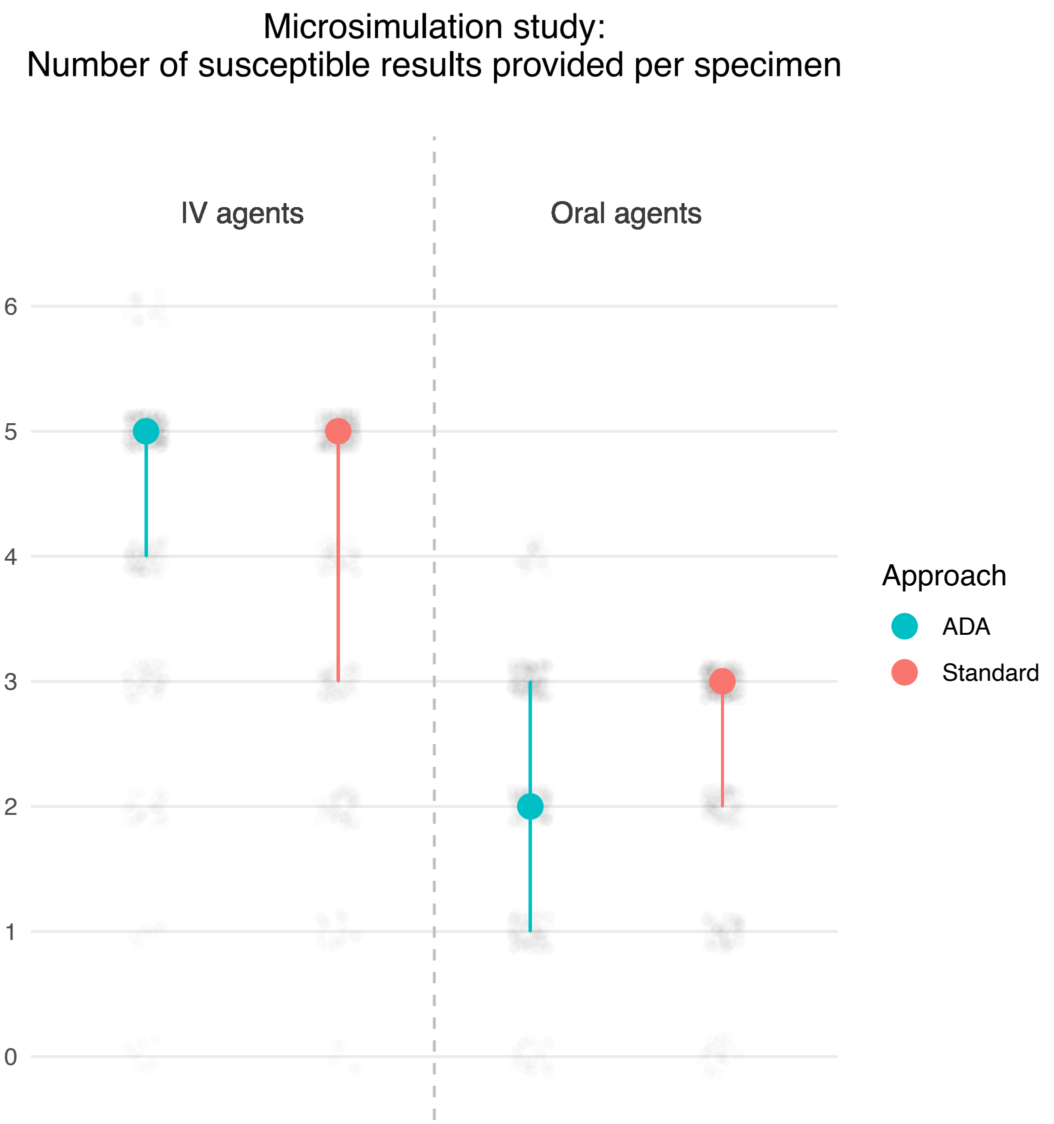

Supplementary Fig. 11: Simulation sub-analysis in patients with coded UTI diagnoses and allergy history

A subset analysis where the simulation study was repeated only on patients with a UTI diagnosis coed for the admission. Plots display the utility distribution (top), antibiotics prescribed (second from top), effect of increasing illness severity (second from bottom), and effect on adaptive antimicrobial susceptibility testing results (bottom).

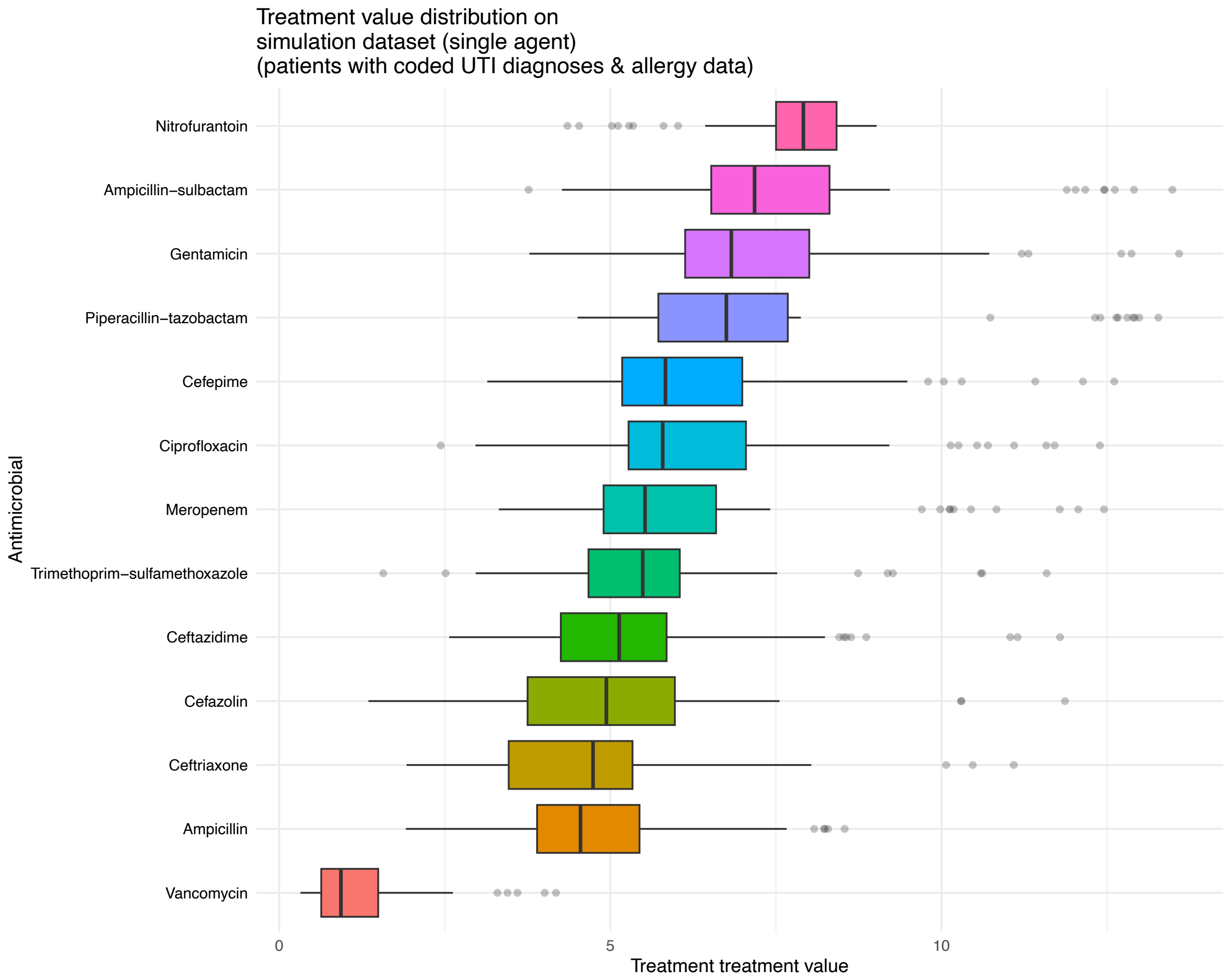
Treatment value distribution on
simulation dataset (single agent)
(patients with coded UTI diagnoses & allergy data)
Nitrofurantoin
Ampicillin−sulbactam
Gentamicin
Piperacillin−tazobactam
Cefepime
Ciprofloxacin
Meropenem
Trimethoprim−sulfamethoxazole
Ceftazidime
Cefazolin
Ceftriaxone
Ampicillin
Vancomycin
Antimicrobial
0
5
10
Treatment treatment value

188

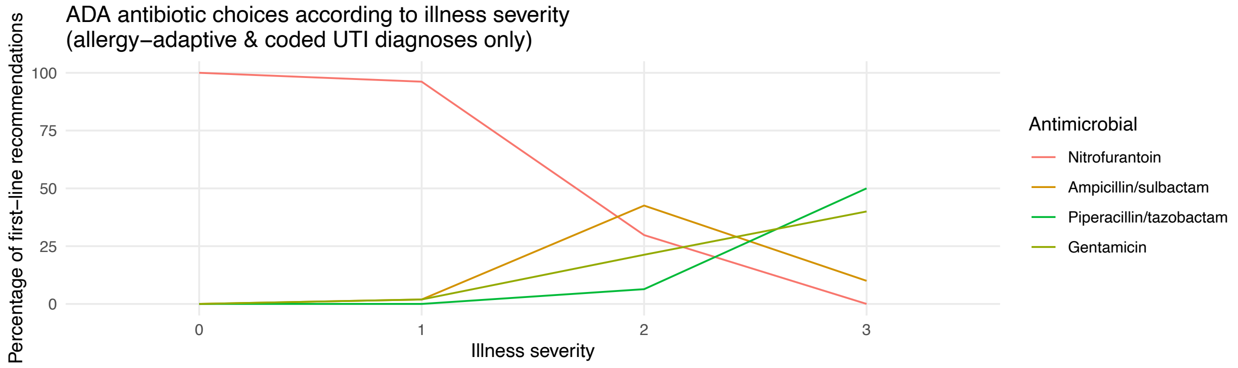
ADA antibiotic choices according to illness severity
(allergy−adaptive & coded UTI diagnoses only)
Percentage of first−line recommendations
100
75
50
25
0
0
1
2
3
Illness severity
Antimicrobial
Nitrofurantoin
Ampicillin/sulbactam
Piperacillin/tazobactam
Gentamicin

189

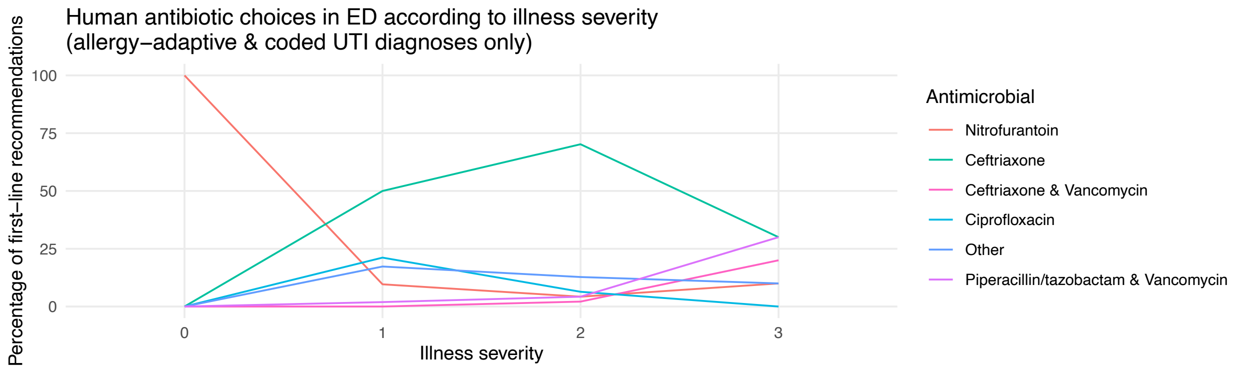
Human antibiotic choices in ED according to illness severity
(allergy−adaptive & coded UTI diagnoses only)
Percentage of first−line recommendations
100
75
50
25
0
0
1
2
3
Illness severity
Antimicrobial
Nitrofurantoin
Ceftriaxone
Ceftriaxone & Vancomycin
Ciprofloxacin
Other
Piperacillin/tazobactam & Vancomycin

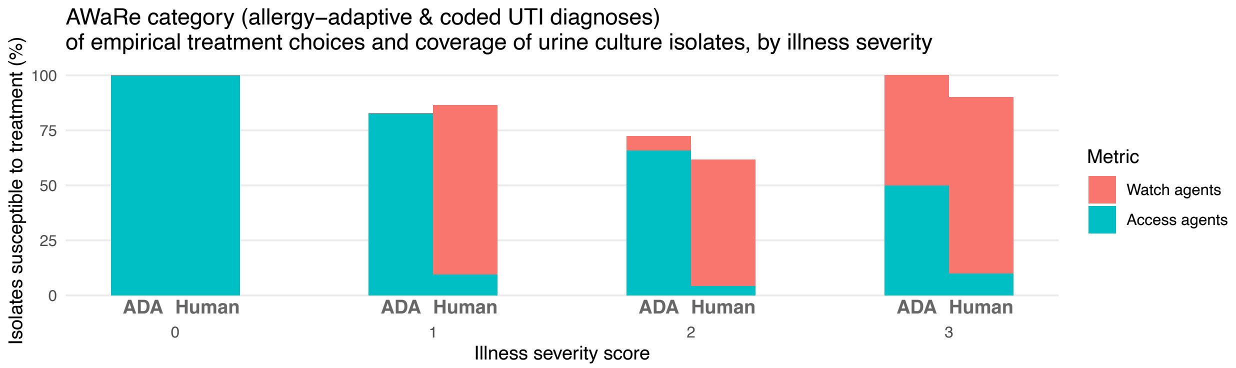
AWaRe category (allergy−adaptive & coded UTI diagnoses)
of empirical treatment choices and coverage of urine culture isolates, by illness severity
Isolates susceptible to treatment (%)
100
75
50
25
0
ADA Human
0
ADA Human
1
ADA Human
2
ADA Human
3
Illness severity score
Metric
Watch agents
Access agents

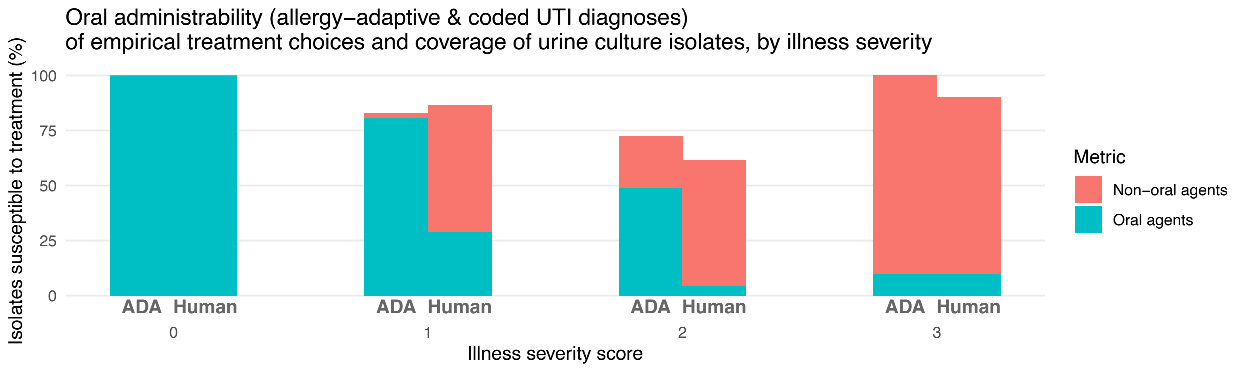
Oral administrability (allergy−adaptive & coded UTI diagnoses)
of empirical treatment choices and coverage of urine culture isolates, by illness severity
Isolates susceptible to treatment (%)
100
75
50
25
0
ADA Human
0
ADA Human
1
ADA Human
2
ADA Human
3
Illness severity score
Metric
Non−oral agents
Oral agents

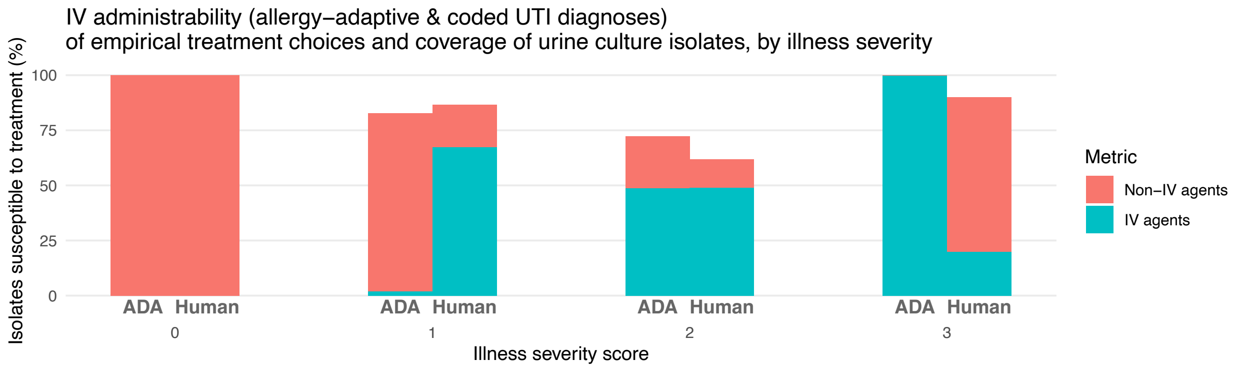
IV administrability (allergy−adaptive & coded UTI diagnoses)
of empirical treatment choices and coverage of urine culture isolates, by illness severity
Isolates susceptible to treatment (%)
100
75
50
25
0
ADA Human
0
ADA Human
1
ADA Human
2
ADA Human
3
Illness severity score
Metric
Non−IV agents
IV agents

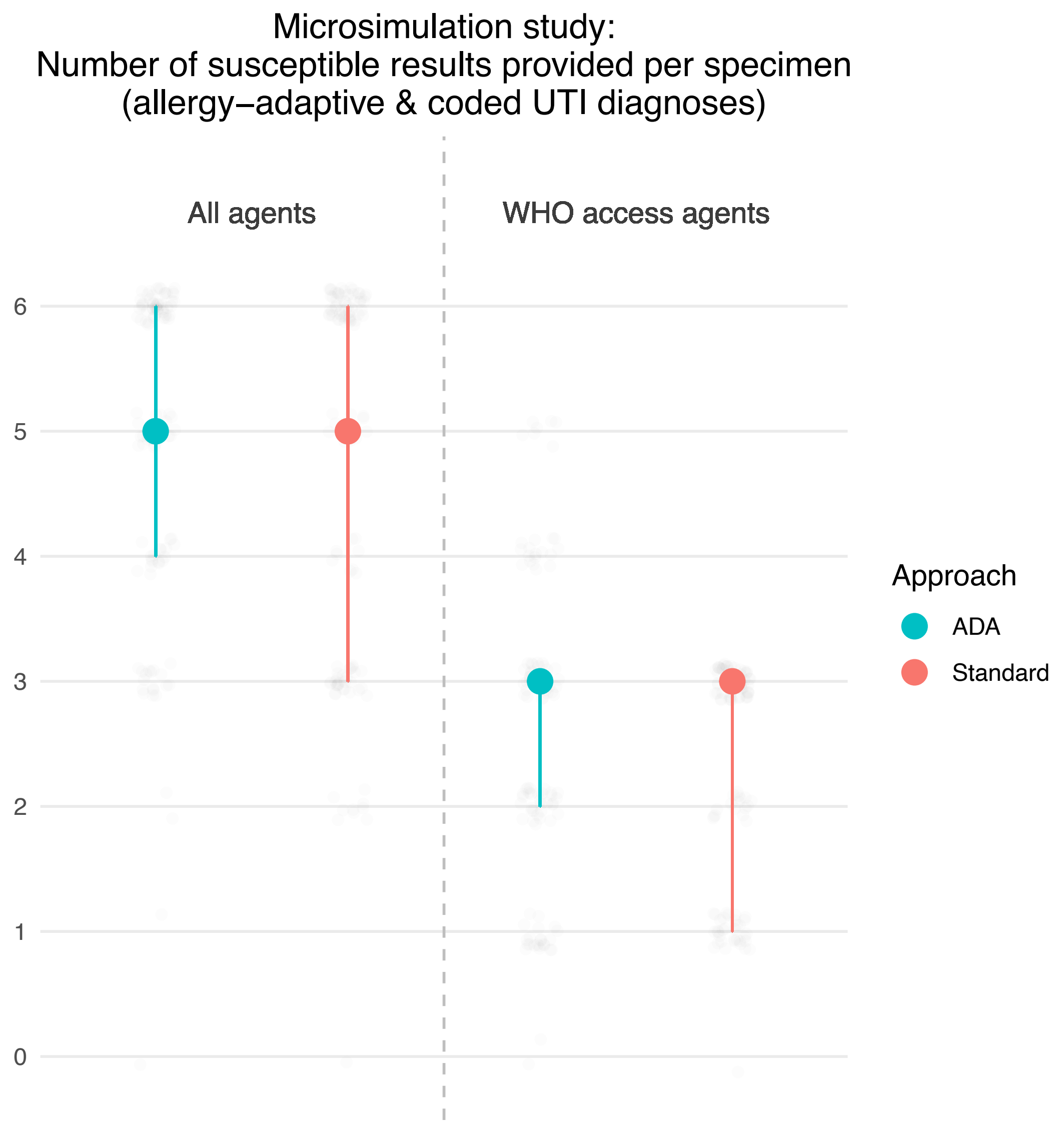
Microsimulation study:
Number of susceptible results provided per specimen
(allergy–adaptive & coded UTI diagnoses)
All agents
WHO access agents
6
5
4
3
2
1
0
Approach
ADA
Standard

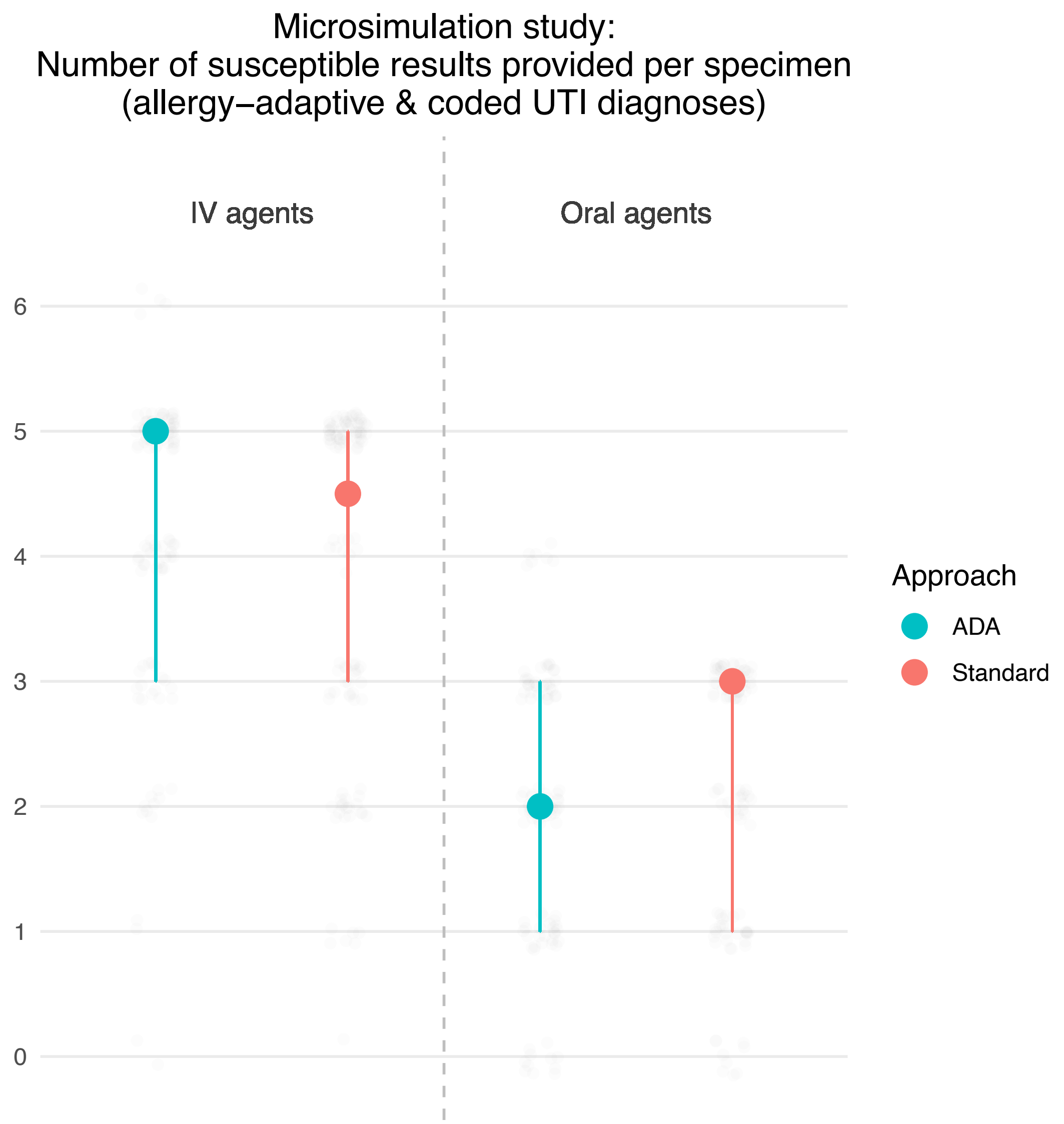

Supplementary Fig. 12: Data processing workflow

Data processing workflow for the urine dataset (top), prescription dataset (middle), and antibiotic choice ranking exercise dataset (bottom). Created in BioRender. Howard, A. (2025)

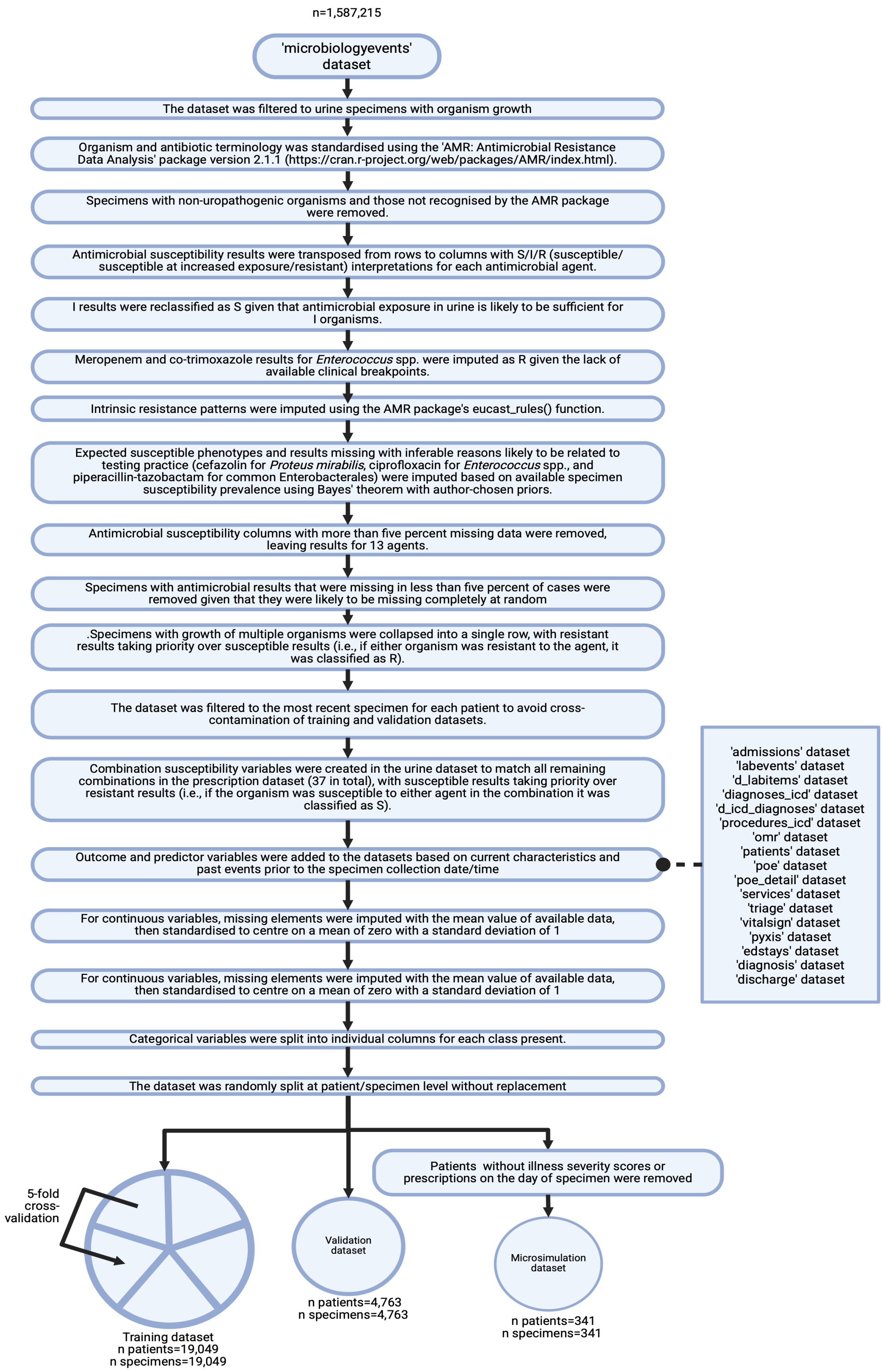
n=1,587,215
'microbiologyevents' dataset
The dataset was filtered to urine specimens with organism growth
Organism and antibiotic terminology was standardised using the 'AMR: Antimicrobial Resistance Data Analysis' package version 2.1.1 (https://cran.r-project.org/web/packages/AMR/index.html).
Specimens with non-uropathogenic organisms and those not recognised by the AMR package were removed.
Antimicrobial susceptibility results were transposed from rows to columns with S/I/R (susceptible/ susceptible at increased exposure/resistant) interpretations for each antimicrobial agent.
I results were reclassified as S given that antimicrobial exposure in urine is likely to be sufficient for I organisms.
Meropenem and co-trimoxazole results for *Enterococcus* spp. were imputed as R given the lack of available clinical breakpoints.
Intrinsic resistance patterns were imputed using the AMR package's eucast_rules() function.
Expected susceptible phenotypes and results missing with inferable reasons likely to be related to testing practice (cefazolin for *Proteus mirabilis*, ciprofloxacin for *Enterococcus* spp., and piperacillin-tazobactam for common Enterobacterales) were imputed based on available specimen susceptibility prevalence using Bayes' theorem with author-chosen priors.
Antimicrobial susceptibility columns with more than five percent missing data were removed, leaving results for 13 agents.
Specimens with antimicrobial results that were missing in less than five percent of cases were removed given that they were likely to be missing completely at random
.Specimens with growth of multiple organisms were collapsed into a single row, with resistant results taking priority over susceptible results (i.e., if either organism was resistant to the agent, it was classified as R).
The dataset was filtered to the most recent specimen for each patient to avoid cross-contamination of training and validation datasets.
Combination susceptibility variables were created in the urine dataset to match all remaining combinations in the prescription dataset (37 in total), with susceptible results taking priority over resistant results (i.e., if the organism was susceptible to either agent in the combination it was classified as S).
Outcome and predictor variables were added to the datasets based on current characteristics and past events prior to the specimen collection date/time
'admissions' dataset
'labevents' dataset
'd_labitems' dataset
'diagnoses_icd' dataset
'd_icd_diagnoses' dataset
'procedures_icd' dataset
'omr' dataset
'patients' dataset
'poe' dataset
'poe_detail' dataset
'services' dataset
'triage' dataset
'vitalsign' dataset
'pyxis' dataset
'edstays' dataset
'diagnosis' dataset
'discharge' dataset
For continuous variables, missing elements were imputed with the mean value of available data, then standardised to centre on a mean of zero with a standard deviation of 1
For continuous variables, missing elements were imputed with the mean value of available data, then standardised to centre on a mean of zero with a standard deviation of 1
Categorical variables were split into individual columns for each class present.
The dataset was randomly split at patient/specimen level without replacement
Patients without illness severity scores or prescriptions on the day of specimen were removed
5-fold cross-validation
Validation dataset
Microsimulation dataset
Training dataset
n patients=19,049
n specimens=19,049
n patients=4,763
n specimens=4,763
n patients=341
n specimens=341

202

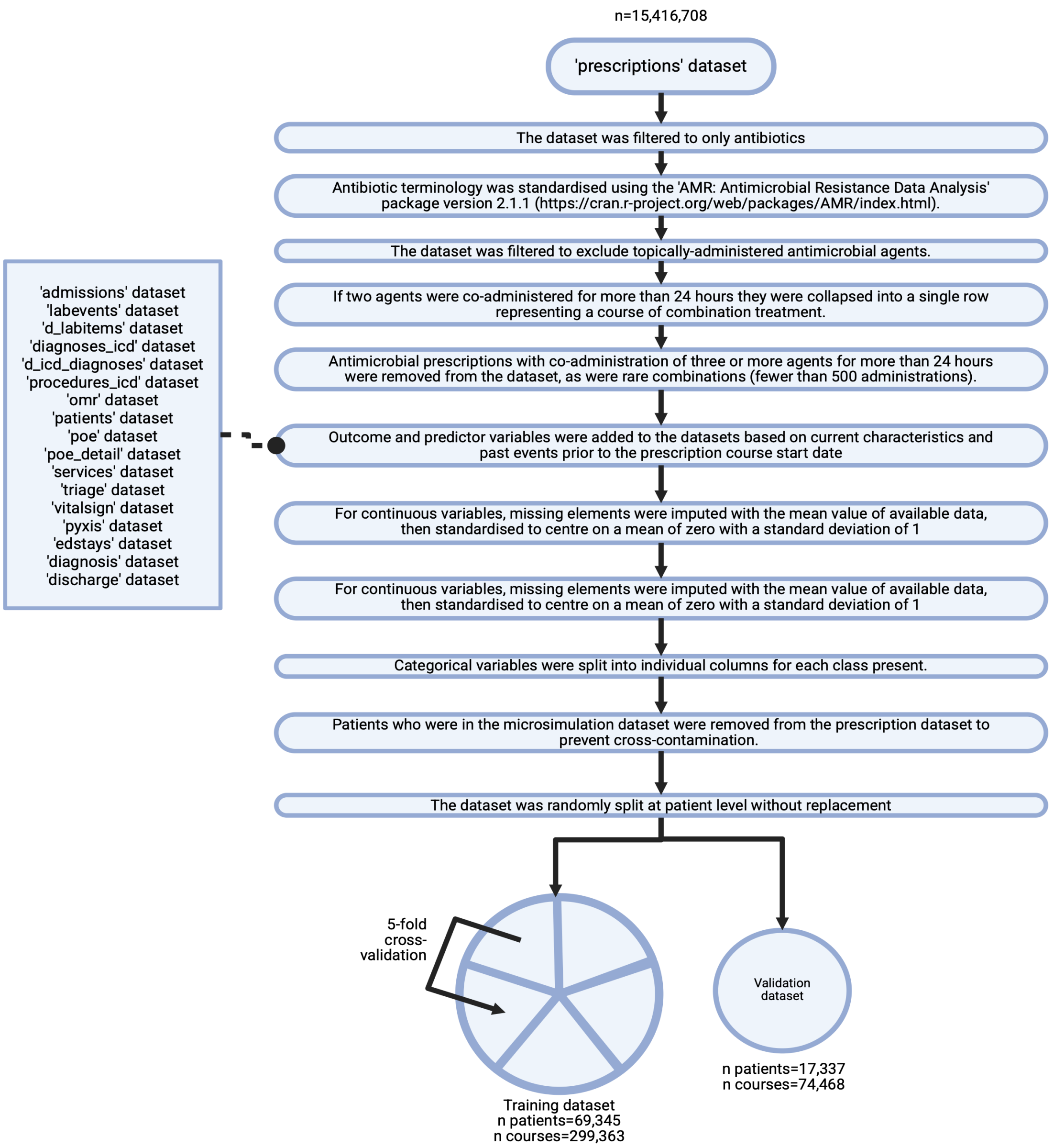

n=15,416,708
'prescriptions' dataset
The dataset was filtered to only antibiotics
Antibiotic terminology was standardised using the 'AMR: Antimicrobial Resistance Data Analysis' package version 2.1.1 (https://cran.r-project.org/web/packages/AMR/index.html).
The dataset was filtered to exclude topically-administered antimicrobial agents.
If two agents were co-administered for more than 24 hours they were collapsed into a single row representing a course of combination treatment.
Antimicrobial prescriptions with co-administration of three or more agents for more than 24 hours were removed from the dataset, as were rare combinations (fewer than 500 administrations).
'admissions' dataset
'labevents' dataset
'd_labitems' dataset
'diagnoses_icd' dataset
'd_icd_diagnoses' dataset
'procedures_icd' dataset
'omr' dataset
'patients' dataset
'poe' dataset
'poe_detail' dataset
'services' dataset
'triage' dataset
'vitalsign' dataset
'pyxis' dataset
'edstays' dataset
'diagnosis' dataset
'discharge' dataset
Outcome and predictor variables were added to the datasets based on current characteristics and past events prior to the prescription course start date
For continuous variables, missing elements were imputed with the mean value of available data, then standardised to centre on a mean of zero with a standard deviation of 1
For continuous variables, missing elements were imputed with the mean value of available data, then standardised to centre on a mean of zero with a standard deviation of 1
Categorical variables were split into individual columns for each class present.
Patients who were in the microsimulation dataset were removed from the prescription dataset to prevent cross-contamination.
The dataset was randomly split at patient level without replacement
5-fold cross-validation
Validation dataset
n patients=17,337
n courses=74,468
Training dataset
n patients=69,345
n courses=299,363

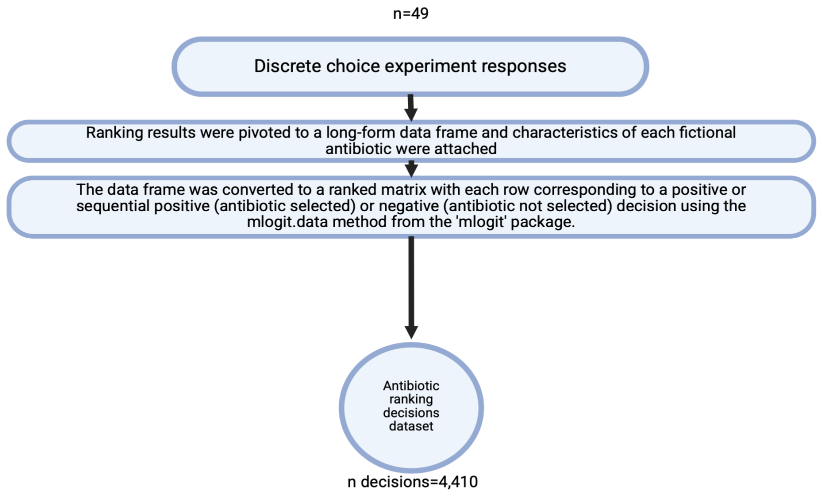

n=49
Discrete choice experiment responses
Ranking results were pivoted to a long-form data frame and characteristics of each fictional antibiotic were attached
The data frame was converted to a ranked matrix with each row corresponding to a positive or sequential positive (antibiotic selected) or negative (antibiotic not selected) decision using the mlogit.data method from the 'mlogit' package.
Antibiotic ranking decisions dataset
n decisions=4,410
